# Structure–Activity Relationship Studies of Aryl Sulfoxides as Reversible Monoacylglycerol Lipase Inhibitors

**DOI:** 10.1021/acs.jmedchem.4c01037

**Published:** 2024-07-11

**Authors:** Ming Jiang, Mirjam C. W. Huizenga, Florian Mohr, Avand Amedi, Renze Bakker, Richard J. B. H. N. van den Berg, Hui Deng, Tom van der Wel, Constant A.A. van Boeckel, Mario van der Stelt

**Affiliations:** † Department of Molecular Physiology, Leiden University and Oncode Institute, Leiden 2333 CC, Netherlands; ‡ Department of Bio-organic Synthesis, Leiden University, Leiden 2333 CC, Netherlands

## Abstract

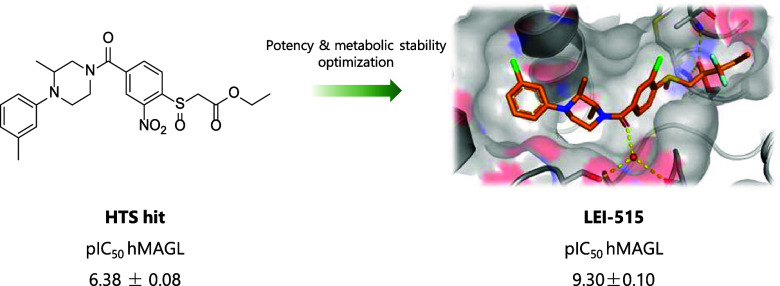

Monoacylglycerol lipase (MAGL) is the key enzyme for the hydrolysis of endocannabinoid 2-arachidonoylglycerol (2-AG). The central role of MAGL in the metabolism of 2-AG makes it an attractive therapeutic target for a variety of disorders, including inflammation-induced tissue injury, pain, multiple sclerosis, and cancer. Previously, we reported **LEI-515**, an aryl sulfoxide, as a peripherally restricted, covalent reversible MAGL inhibitor that reduced neuropathic pain and inflammation in preclinical models. Here, we describe the structure–activity relationship (SAR) of aryl sulfoxides as MAGL inhibitors that led to the identification of **LEI-515**. Optimization of the potency of high-throughput screening (HTS) hit **1** yielded compound **±43**. However, **±43** was not metabolically stable due to its ester moiety. Replacing the ester group with α-CF_2_ ketone led to the identification of compound **±73** (**LEI-515**) as a metabolically stable MAGL inhibitor with subnanomolar potency. **LEI-515** is a promising compound to harness the therapeutic potential of MAGL inhibition.

## Introduction

2-Arachidonoylglycerol (2-AG) is an endogenous agonist of the cannabinoid CB1 and CB2 receptors and serves as a precursor for a pool of arachidonic acid (AA) which may form pro-inflammatory prostaglandins in the brain, lung, and liver.^1^ The central role of monoacylglycerol lipase (MAGL) in the metabolism of 2-AG makes it, therefore, an attractive therapeutic target for a variety of disorders, including inflammation-induced tissue injury and pain, multiple sclerosis, and cancer.^2,3^ MAGL is a membrane-associated serine hydrolase and employs a serine-histidine-aspartate catalytic triad to hydrolyze the ester moiety of monoacylglycerols.^4^ Currently, the covalent, irreversible MAGL inhibitor ABX-1431 is in clinical phase 1b studies for the treatment of post-traumatic stress disorder as well as for other indications, such as neuromyelitis optica and multiple sclerosis.^5^ Irreversible inhibitors may have several advantages to act as therapeutics, for instance, increased potency, long residence time, and a less stringent pharmacokinetic profile.^6^ However, the irreversible mode of action may also have some drawbacks, such as reduced selectivity and the formation of covalent-protein adducts, which might result in idiosyncratic drug-related toxicity.^7^ In the case of MAGL inhibition, chronic exposure to the covalent inhibitor JZL184 resulted in pharmacological tolerance, development of physical dependence, impaired synaptic plasticity, and receptor desensitization in the nervous system.^8,9^ Reversible inhibitors may avoid these unfavorable side effects.^10−19^

**Figure 1:**
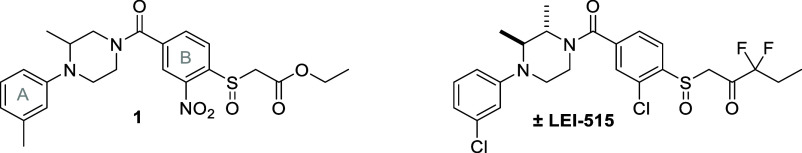
Chemical structure of HTS hit **1** and **LEI-515**.

To harness the therapeutic potential of MAGL, a high-throughput screen (HTS) was previously performed to identify novel reversible MAGL inhibitors. A natural substrate assay was employed that utilizes an enzymatic cascade to convert glycerol, a metabolite produced by MAGL, into a fluorescent signal.^20^ A compound library containing 233,820 unique structures was screened, which yielded β-sulfinyl ester **1** with a half maximal inhibitory concentration (IC50) of 630 nM (Figure 1). Here, a structure–activity relationship (SAR) of hit **1** is described, which led to the identification of **LEI-515** (Figure 1) as the most potent reversible and peripherally restricted inhibitor for MAGL up to date (pIC_50_ 9.30 ± 0.04). **LEI-515** increased 2-AG levels both in live cells as well as in peripheral mouse organs in vivo*.* Moreover, **LEI-515** reduced inflammation in an acute liver injury mouse model and was able to suppress chemotherapy-induced neuropathic pain in mice. In addition, **LEI-515** did not induce central nervous system (CNS) adverse effects or physical dependence.^21^

## Results and Discussion

### Hit Optimization of β-Sulfinyl Esters as Highly Potent MAGL Inhibitors

Hit **1** contains a chiral center in the piperazine moiety To investigate which enantiomer is the most active compound, both the (*S*)**-1** and (*R*)-**1** enantiomers were synthesized and tested. Both compounds showed similar inhibitory potencies as the initial hit (Table 1), indicating that the chirality of the methyl substituent at the 3-position of piperazine did not impact the inhibition of MAGL. On the other hand, removal of the methyl group (**2**) led to a 10-fold drop in activity. The oxidation state of the sulfur atom in compound (**1**) was important because reducing the sulfoxide to sulfur (**3**) or oxidizing it to a sulfonyl (**4**) abolished the activity. The removal of the nitro-group (**5**) also led to a significant reduction in activity.

**Table 1:**
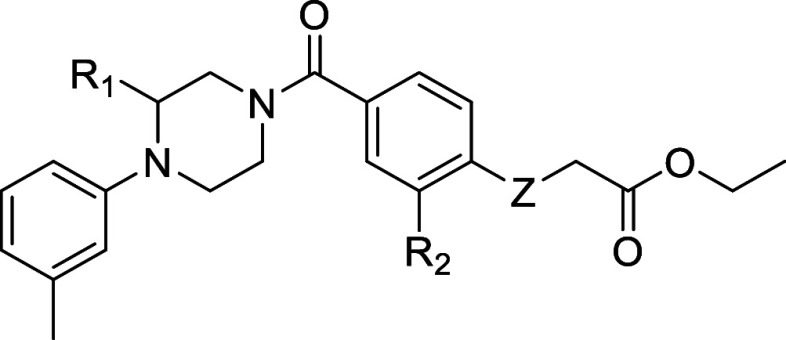
pIC_50_ Values of Resynthesized Hit **1** and Designed Derivatives **2–5**

**entry**	**R**_**1**_	**R**_**2**_	**Z**	**pIC_50_ ± SD**
(*S*)**-1**	(*S*)CH_3_	NO_2_	SO	6.57 ± 0.13
(*R*)**-1**	(*R*)CH_3_	NO_2_	SO	6.71 ± 0.05
**2**	H	NO_2_	SO	5.65 ± 0.05
**3**	(*S*)CH_3_	NO_2_	S	<5
**4**	(*S*)CH_3_	NO_2_	SO_2_	<5
**5**	(*S*)CH_3_	H	SO	5.25 ± 0.05

To analyze the effect of the substitution pattern on phenyl A, compounds **6**-**22** were evaluated (Table 2) using the scaffold of compound **2** (for ease of synthesis). Electron donating substituents on the *meta*- (methyl (**2**), methoxy (**15**)) or *para*-position (methoxy (**16**)) reduced the potency compared to compound (**6**). In contrast, electron-withdrawing groups (EWG) were preferred on the *meta*-position (F (**7**), Cl (**8**), Br (**11**), CF_3_ (**13**), but not nitro (**17**). The electron-withdrawing effect was absent or less pronounced on the *para*-position (Cl (**9**), Br (**12**), or CF_3_ (**14**). Of interest, a phenyl substitution (**18**) was tolerated at the *meta*-position, suggesting the presence of a hydrophobic pocket. Dichloro substitution did not improve the potency (**19**–**22**).

**Table 2:**
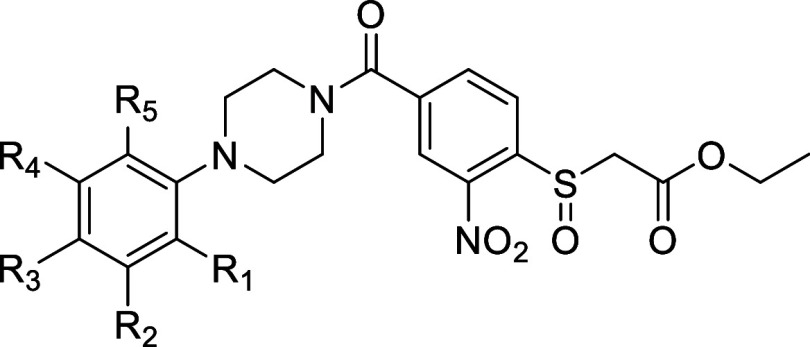
pIC_50_ Values of Designed Analogues **6–22**

**entry**	**R**_**1**_	**R**_**2**_	**R**_**3**_	**R**_**4**_	**R**_**5**_	**pIC_50_ ± SD**
**2**	H	CH_3_	H	H	H	5.65 ± 0.05
**6**	H	H	H	H	H	5.91 ± 0.11
**7**	H	F	H	H	H	6.20 ± 0.18
**8**	H	Cl	H	H	H	6.33 ± 0.08
**9**	H	H	Cl	H	H	6.00 ± 0.08
**10**	Cl	H	H	H	H	5.37 ± 0.07
**11**	H	Br	H	H	H	6.44 ± 0.06
**12**	H	H	Br	H	H	5.95 ± 0.08
**13**	H	CF_3_	H	H	H	6.42 ± 0.09
**14**	H	H	CF_3_	H	H	6.23 ± 0.13
**15**	H	OCH_3_	H	H	H	5.18 ± 0.07
**16**	H	H	OCH_3_	H	H	5.31 ± 0.04
**17**	H	NO_2_	H	H	H	5.59 ± 0.06
**18**	H	phenyl	H	H	H	5.87 ± 0.08
**19**	H	Cl	H	Cl	H	6.31 ± 0.04
**20**	H	Cl	Cl	H	H	5.59 ± 0.11
**21**	Cl	H	Cl	H	H	5.71 ± 0.13
**22**	Cl	H	H	H	Cl	5.74 ± 0.13

Next, an EWG at the *meta*-position of phenyl A was combined with the chiral substituted piperazines (**23**–**26**) (Table 3). Substitution of the *meta*-methyl of the toluyl group with a halogen on the chiral pure scaffold of compound **1** increased the inhibitory potency on both enantiomers equally well. The *meta*-chloro-substituted (*R*)**-24** and (*S*)**-24** were the most active compounds with a pIC_50_ around 7.4.

**Table 3:**
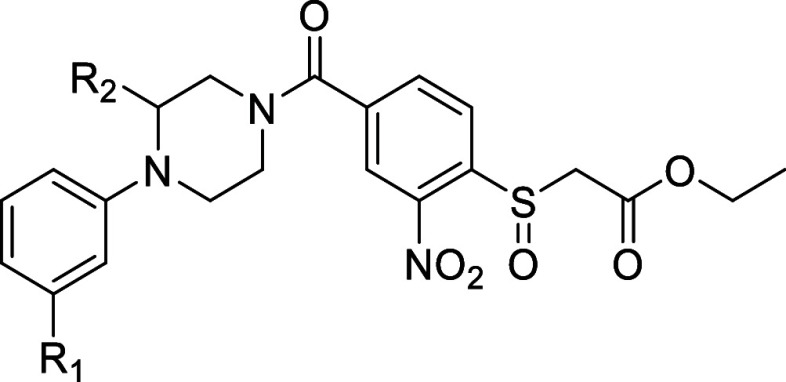
pIC_50_ Values of Designed Analogues **23–26**

**entry**	**R**_**1**_	**R**_**2**_	**pIC_50_ ± SD**
(*S*)**-1**	CH_3_	(*S*)CH_3_	6.57 ± 0.13
(*R*)**-1**	CH_3_	(*R*)CH_3_	6.71 ± 0.05
**23**	F	(*S*)CH_3_	6.81 ± 0.03
(*R*)**-24**	Cl	(*R*)CH_3_	7.40 ± 0.11
(*S*)**-24**	Cl	(*S*)CH_3_	7.36 ± 0.08
(*R*)**-25**	Br	(*R*)CH_3_	7.06 ± 0.07
(*S*)**-25**	Br	(*S*)CH_3_	7.09 ± 0.06
(*R*)**-26**	CF_3_	(R)CH_3_	6.94 ± 0.04
(*S*)**-26**	CF_3_	(*S*)CH_3_	6.70 ± 0.08

Employing the scaffold of (*R*)**-24**, which was the most active compound identified thus far, the SAR of the ester moiety was studied. To this end, compounds **27**-**35** were evaluated (Table 4). Replacement of the ethyl ester with methyl (**27**) or trifluoroethyl (**30**) esters resulted in decreased MAGL activity while elongating the alkyl chain to a propyl (**31**) or butyl (**32**) increased the potency compared to the ethyl (**24**). Of note, branching of the alkyl chain (isopropyl (**29**), *sec*-butyl (**33**) and *tert*-pentyl (**34**)) reduced the activity. The introduction of a polar group was tolerated, as witnessed by hydroxypropyl ester (**35**), which had a similar activity as (*R*)**-24**. However, changing the ester to a tertiary amide (**28**) resulted in an inactive compound.

**Table 4:**
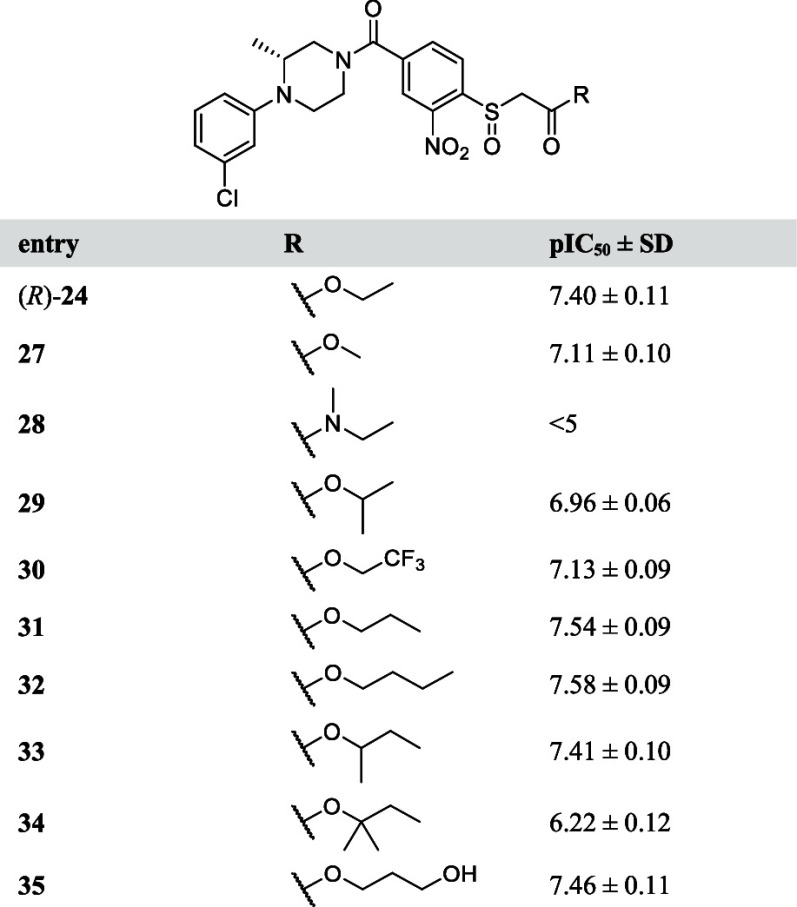
pIC_50_ Values of Ester and Amide Analogues **27–35**

Next, the SAR of the piperazine and the 2-nitrophenyl ring was revisited. Various methyl-substituted piperazines and the nitro-group bioisosteres (compounds **36**–**44**) were analyzed (Table 5). An additional methyl group at the 5-position (**36**) decreased the MAGL inhibitory activity. Replacing the nitro group with a fluorine (**37**) in the scaffold maintained the activity, and, importantly, reduced the liability for potential genotoxicity. 2-Methyl-piperazine (**38**) had similar MAGL inhibitory activity as compound **37**. 2,2-Dimethyl (**42**) or 3,3-dimethyl (**41**) substitution resulted in decreased potency as compared to 3-methyl substituted piperazines. Interestingly, trans-2,3-dimethyl substitution (**±40**) significantly improved the potency, while cis-2,3-dimethyl substitution (**±39**) slightly decreased the MAGL activity compared to compound **37**. Furthermore, changing the fluoro substituent to chloro (**±43**) and bromo (**±44**) groups further increased the potency. Compound **±43** was the most potent compound identified with a pIC_50_ of 8.50 ± 0.10.

**Table 5:**
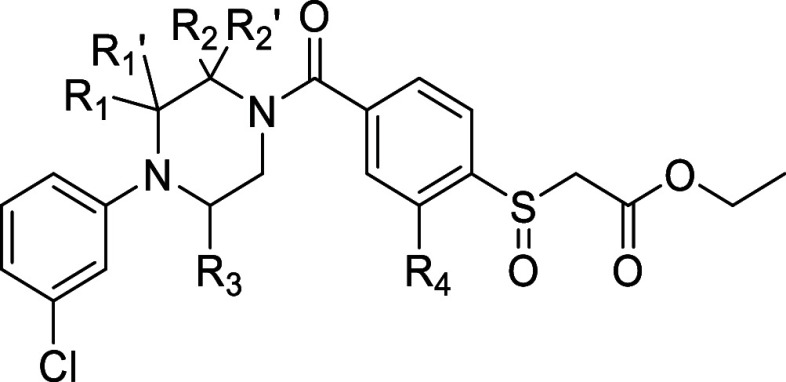
pIC_50_ Values of Designed Analogues **36–44**

**entry**	**R**_**1**_	**R**_**1**_**′**	**R**_**2**_	**R**_**2**_**′**	**R**_**3**_	**R**_**4**_	**pIC_50_ ± SD**
(*R*)**-24**	(*R*)CH_3_	H	H	H	H	NO_2_	7.40 ± 0.11
**36**	CH_3_	H	H	H	CH_3_	NO_2_	6.68 ± 0.07
**37**	(*R*)CH_3_	H	H	H	H	F	7.56 ± 0.04
**38**	H	H	CH_3_	H	H	F	7.56 ± 0.10
**±39**	cis-CH_3_	H	cis-CH_3_	H	H	F	7.29 ± 0.07
**±40**	trans-CH_3_	H	trans-CH_3_	H	H	F	8.13 ± 0.07
**41**	CH_3_	CH_3_	H	H	H	F	7.33 ± 0.07
**42**	H	H	CH_3_	CH_3_	H	F	7.24 ± 0.07
**±43**	trans-CH_3_	H	trans-CH_3_	H	H	Cl	8.50 ± 0.10
**±44**	trans-CH_3_	H	trans-CH_3_	H	H	Br	8.24 ± 0.17

Finally, based on the potency of compound **±43**, several analogues (**±45**–**±55**) were evaluated (Table 6). Hydrolysis of the ethyl ester to carboxylic acid (**±45**) resulted in >500-fold loss of MAGL inhibitory activity while replacing the linker amide to amine (**±48**) was allowed. Changing the sulfinyl group to sulfur (**±46**) abolished the inhibitory activity and replacing it with a sulfonyl (**±47**) or carbonyl (**±49**) resulted in a 1000-fold reduced inhibitory activity. Compounds in which the ethyl ester was replaced with an isopropyl (**±50**), *sec*-butyl (**±51**), cyclobutyl (**±52**), cyclopentanyl (**±53**), or cyclohexanyl (**±54**) esters displayed similar MAGL inhibitory activity but decreased lipophilic efficiency (LipE) compared to compound **±43**. The polar 1-glycerol ester (**±55**) showed similar potency compared to the other esters. Altogether, this SAR study revealed that compound **±43** showed the most promising combination of activity and physio-chemical properties (Table 6).

**Table 6:**
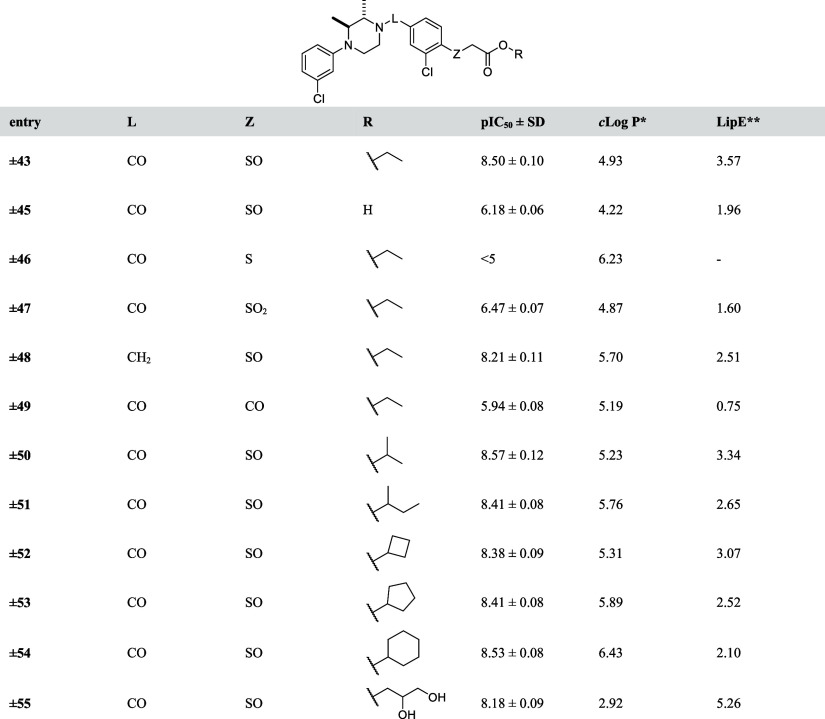
pIC_50_ Values of Designed Analogues **45–55**

* *c*Log *P* calculated with chemdraw 19.0;

** LipE = pIC_50_ – *c*Log *P*.

### Discovery of Activated Ketones as MAGL Inhibitors with Improved Metabolic Stability

The metabolic stability of compound **±43** was evaluated by using a liver S9 stability assay. The compound was incubated with liver S9 fraction and the amount of remaining unmetabolized compound was determined with liquid chromatography–mass spectrometry/mass spectrometry (LC-MS/MS) in a time-dependent manner. The results are presented as intrinsic clearance (Cl_int_), which is calculated as *V* × 0.693/*t*_1/2_ (μL/min/mg) in which V is the volume of incubation in μL per mg protein and *t*_1/2_ is the measured half-life in min. Compound **±43** showed a high Cl_int_ (>346 μL/min/mg, Table 7), revealing that compound **±43** is rapidly metabolized. This is not surprising, because the compound has high lipophilicity (calculated octanol–water partition coefficient (*c*Log *P*) of 4.9) and contains a potential metabolically labile ester functionality. Since it is well-known that reducing lipophilicity may increase metabolic stability, several analogues (compound **±56**–**±60**) were synthesized in which phenyl ring A was replaced with different pyridyls (compound **±56**–**±59**) or in which the ethyl ester was substituted with a more polar group (compound **±60**). Of note, compounds (**±56**–**±60**) showed high MAGL inhibitory activities (Table 7) compared to compound **±43**. The metabolic stability of these compounds was, however, not improved (Table 7). This indicated that Cl_int_ of compound **±43** cannot be improved by only reducing the lipophilicity and suggested that the ester was the main metabolic hot spot. To test this hypothesis, compound **±61** was synthesized in which the ester moiety was replaced by a metabolically stable ether. Indeed, the intrinsic clearance dropped significantly from >346 (μL/min/mg) to <4 (μL/min/mg), indicating that the ester group was the primary site of metabolism. Of note, as expected compound **±61** displayed no MAGL inhibitory activity anymore.

**Table 7:**
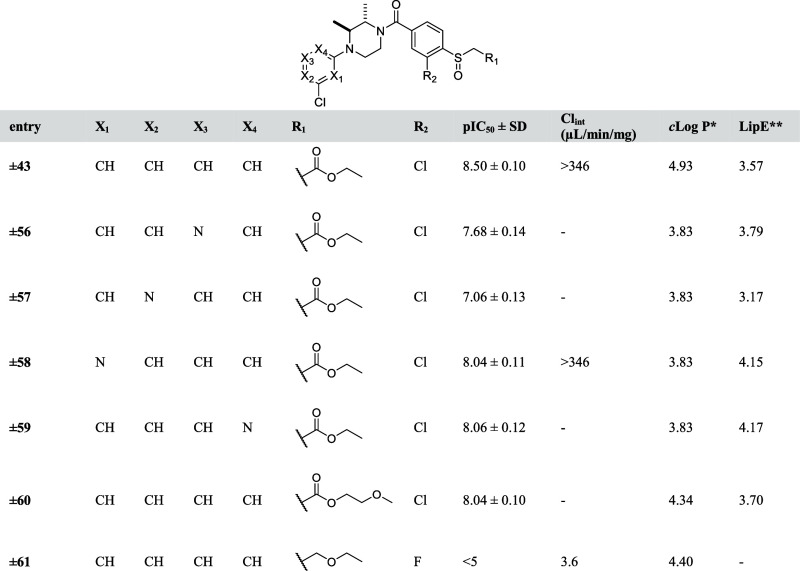
pIC_50_ Values and Intrinsic Clearance (Cl_int_) of Designed Analogues **56–61**

* *c*Log *P* calculated with chemdraw 19.0;

** LipE = pIC_50_ – *c*Log *P*.

Introducing steric hindrance has been previously successfully applied as a strategy to stabilize ester functionalities by preventing the attack of a catalytic serine of carboxylesterases on the carbonyl.^22^ Here, this strategy was employed by introducing a methyl group on the alpha-carbon (compounds **±62** and **±63**) or next to the oxygen (compound **±64**) (Table 8). While compounds (**±62** and **±64**) showed slightly decreased MAGL inhibitory activity, they were still rapidly metabolized with a Cl_int_ > 346 μL/min/mg. Introducing a bulkier group, such as 3,4-methylenedioxybenzyl group in compound (**±65**), which was previously used in an in vivo active MAGL inhibitor^10^ also did not improve the metabolic stability (Table 8).

**Table 8:**
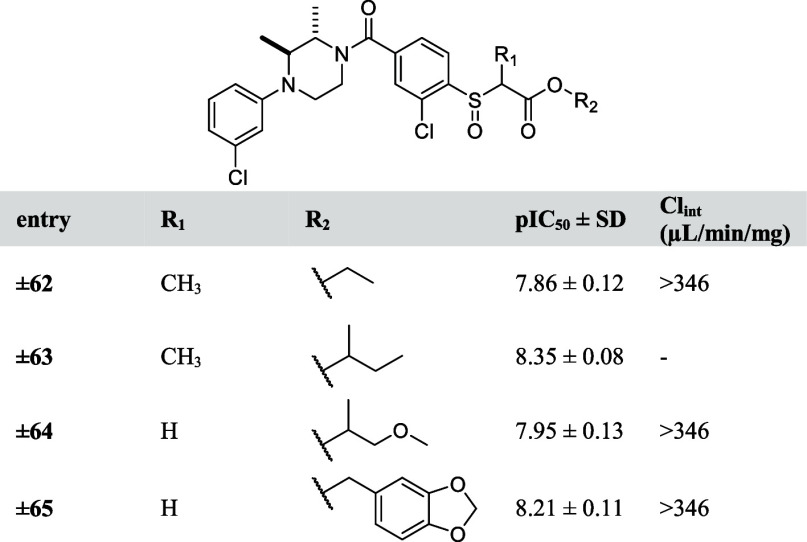
pIC_50_ Values and Intrinsic Clearance (Cl_int_) of Designed Analogues **62–65**

Because introducing steric hindrance did not improve metabolic stability, another strategy was employed in which the metabolically liable ester group was replaced with a bioisoster, such as an amide (**±66**) or an oxadiazole (**±67**). However, compounds **±66** and **±67** are not active MAGL inhibitors (pIC_50_ < 5) and therefore not tested in the metabolic stability assay. Previously, activated ketones were described as inhibitors for serine hydrolases such as fatty acid amide hydrolase (FAAH) and diacylglycerol lipase (DAGL).^23,24^ Inspired by these studies, we explored if activated ketones would yield potent MAGL inhibitors. Compound **±69** with a trifluoromethylketone showed high MAGL inhibitory activity (pIC_50_ = 8.1) (Table 9) and was the most stable compound with a Cl_int_ of 19 μL/min/mg. Of note, if the carbonyl was reduced to the corresponding hydroxyl (**±70**), the compound was inactive (pIC_50_ < 5). This is in line with the hypothesis that the carbonyl acts as an electrophilic warhead for the nucleophilic serine of MAGL. Moreover, the sulfinyl group was not essential for the MAGL inhibitory activity, as compound **±68** still showed reasonable inhibitory activity with a pIC_50_ = 7.8. Replacing the trifluoromethyl group with difluoromethyl (**±71**) increased the MAGL inhibitory activity by 3-fold, but slightly decreased the metabolic stability (Cl_int_ = 35 μL/min/mg). Substituting fluorine with a phenyl group resulted in compound **±74**, which decreased the potency 50-fold. Changing the trifluoromethyl group to difluoroethyl (**±72**) or difluoropropyl (**±73**), however, significantly improved the potency. Compound **±73** (**LEI-515**) is the most potent compound identified in this study with subnanomolar potency (pIC_50_ = 9.3). Importantly, both compounds **±72** and **±73** displayed good metabolic stability (Cl_int_ = 27 and 30 μL/min/mg, respectively). Finally, compound **±75**, in which phenyl ring A was replaced with a pyridyl to reduce the lipophilicity, was synthesized. Compound **±75** showed high MAGL inhibitory activity and enhanced lipophilic efficiency, however, its metabolic stability was significantly decreased. This might be possibly attributed to the potential reactivity of the chloropyridine moiety.^25,26^

**Table 9:**
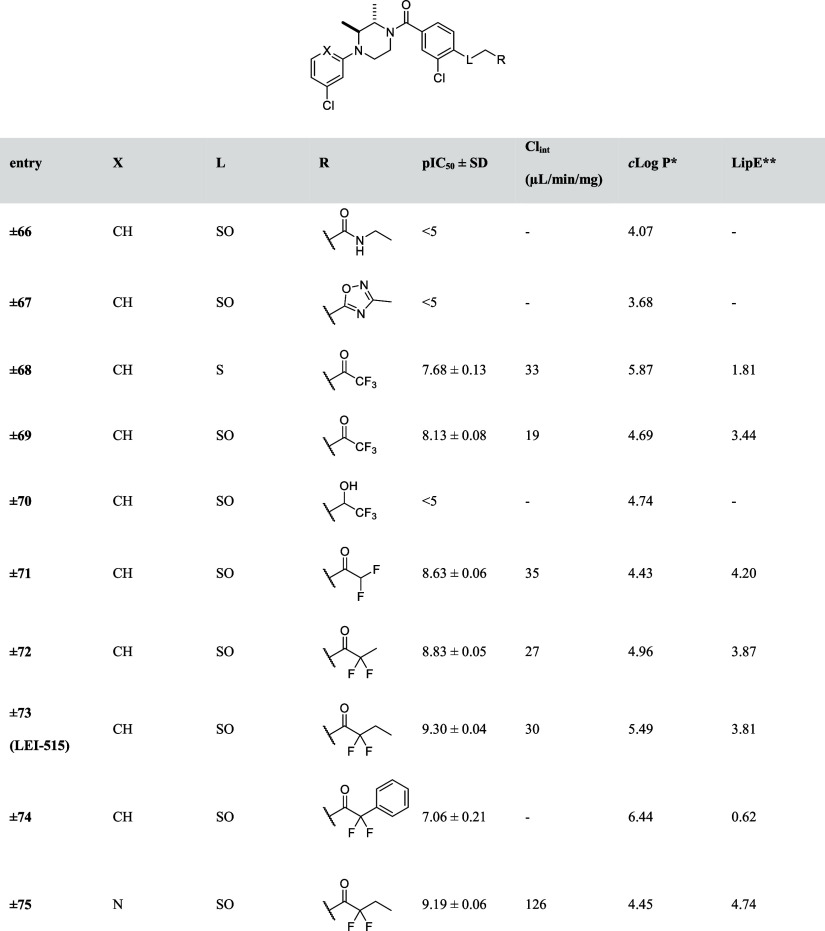
pIC_50_ Values of Designed Analogues **66–75**

* *c*Log *P* calculated with chemdraw 19.0;

** LipE = pIC_50_ – *c*Log *P*.

To assess the influence of the sulfoxide chirality in **±73 (LEI-515)** on the potency, both sulfoxide enantiomers were synthesized using asymmetric thioether oxidation^27^ (Scheme S1) and tested in the natural substrate assay. **(+)-73** was 5-fold more active than the other enantiomer **((−)-73**) (Table 10).

**Table 10:**
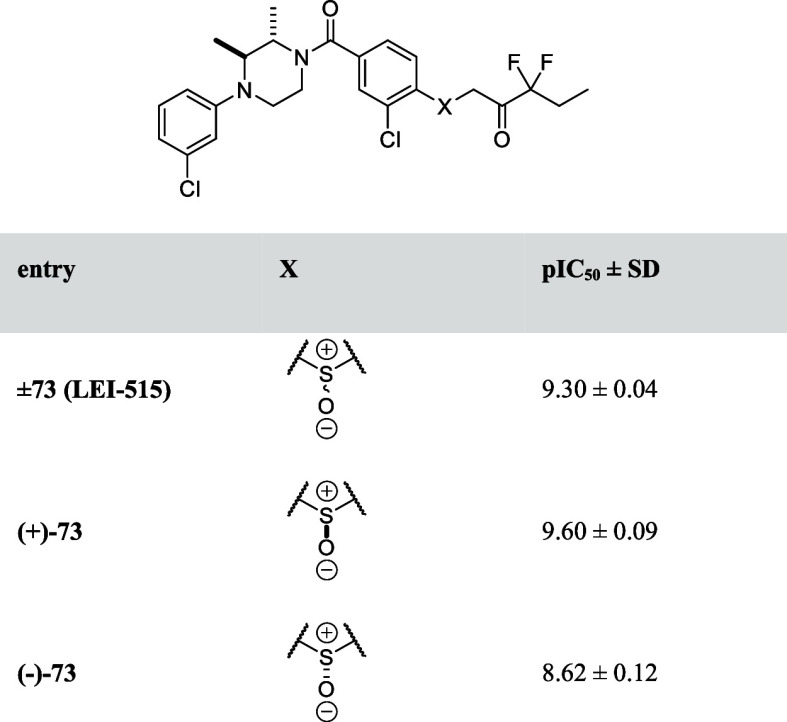
pIC_50_ Values of Chiral Sulfoxides of **73** (**LEI-515**)

### Co-Crystal Structure of MAGL with **LEI-515**

**Figure 2:**
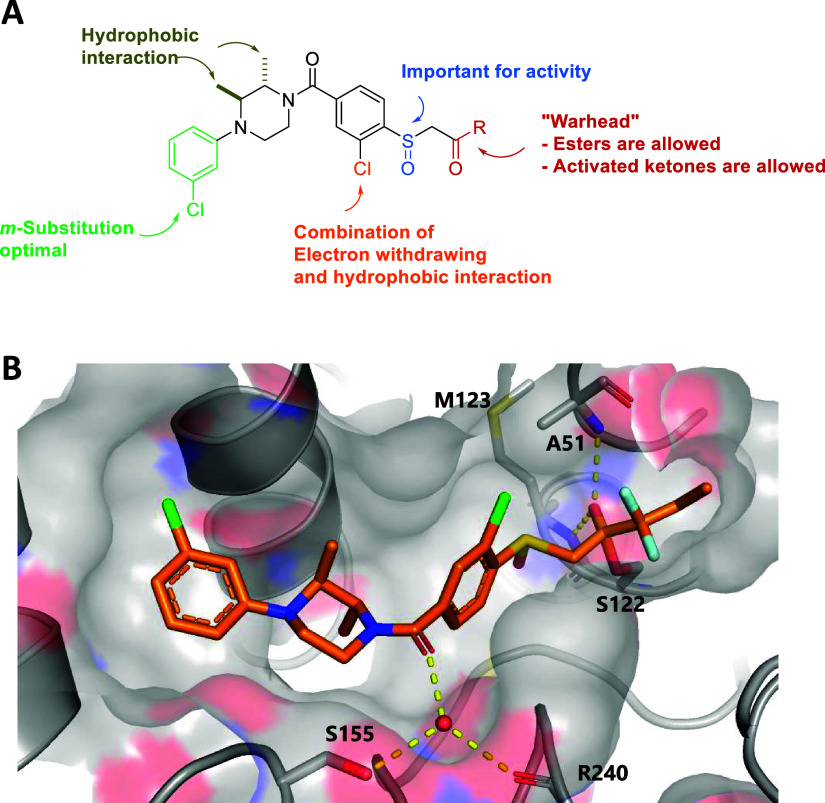
(A) Overall structure–activity relationship. (B) X-ray structure of (2*S*,3*S*)-isomer of **LEI-515** bound to hMAGL (1.55 Å resolution, PDB: 8AQF).^28^**LEI-515** binds to MAGL through covalently reversible mechanism and the deprotonated hemiketal forms two hydrogen bounds (yellow dotted line) with A51 and M123.

A summary of the SAR is shown in Figure 2A. To explain the SAR, we inspected the cocrystal structure of MAGL with **LEI-515** (Figure 2B). The cocrystal structure revealed that the difluorocarbonyl of **LEI-515** binds covalently to the catalytic S122 of MAGL and forms a hemiketal, which is stabilized through hydrogen bonds with the backbone of A51 and M123 as part of the oxyanion hole (PBD: 8AQF).^21^ This indicates that the ester analogues (Tables 1–8) may function as substrate-mimics that form a covalent-reversible bond, which is slowly hydrolyzed. The chiral sulfoxide was important for the potency as the thioether analogues were either inactive or exhibited reduced potencies (Tables 1, 6, and 9), which may result from increased electrophilicity of the carbonyl. The absolute stereochemistry of the enantiomers was not determined, but **±73 (LEI-515)** adopts an (*R*)-sulfoxide configuration in the cocrystal structure. The carbonyl of the amide forms a water-mediated hydrogen bond with S155 and R240. Although the methylene analogue (**±48**) had only slightly lower potency compared to its carbonyl counterpart (**±45**), the carbonyl was important for selectivity (data not shown). A significant positive correlation was found between the pIC_50_ values and the *c*Log *P*, highlighting the influence of lipophilicity on the potency for MAGL inhibitors (Figure S1). Specifically, the *trans*-dimethyl groups on the piperazine fitted into hydrophobic pockets and adopted a diaxial conformation, thereby orientating the chlorophenyl substituent into a hydrophobic pocket.

### Chemistry

**Scheme 1:**
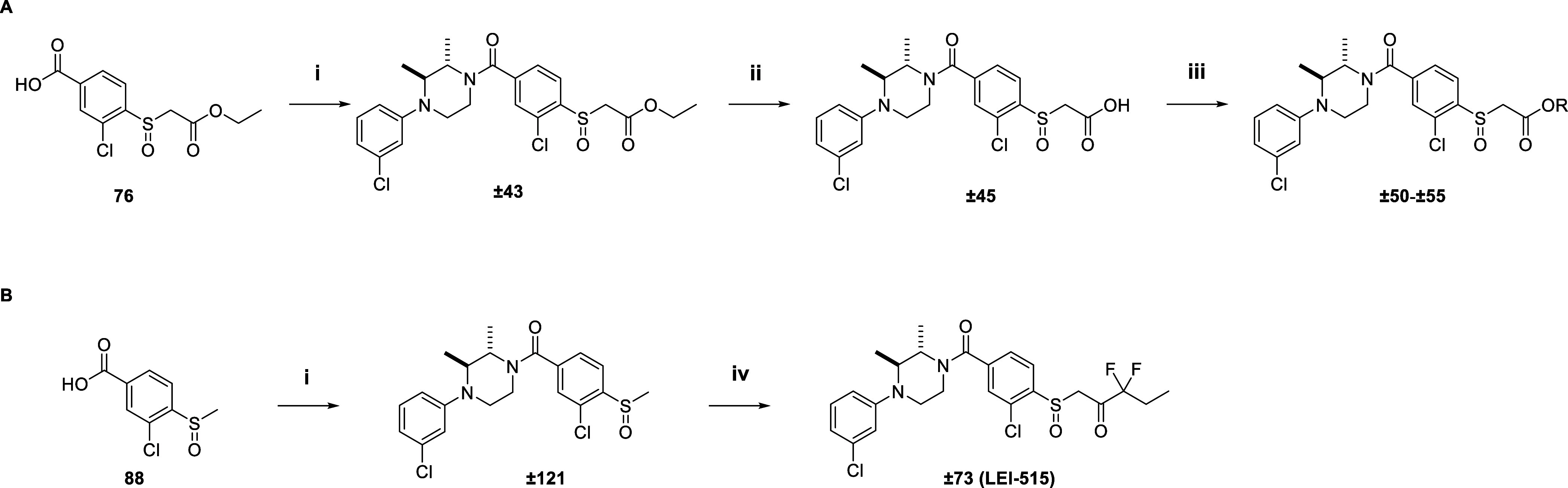
Synthesis Route of Compounds **±43**, **±45**, **±50–±55 (A),** and **±73 (B)** Reagents and conditions: (i) HATU, DiPEA, **±114**, DCM, rt; (ii) TEA, MeOH, H_2_O, rt; (iii) oxalyl chloride, DiPEA, DCM, 0 °C, then appropriate alcohol, 0 °C – rt; (iv) LDA, ethyl 2,2-difluorobutanoate, THF, −78 °C – rt.

The synthesis for all final compounds in Tables 1–10 followed a generic synthetic route (Scheme 1). Scheme 1 depicts the synthesis of compounds **±43**, **±45**, **±50–±55,** and **±73**. Compound **±43** was synthesized using an amide coupling between benzoic acid **77**, HATU, and amine **±144**. Subsequent saponification of **±43** yielded compound **±45**, which was transformed in an acyl chloride and coupled with the appropriate alcohols to yield compounds **±50–±55** (Scheme 1A). The activated ketone **±73 (LEI-515)** was synthesized according to Scheme 1B. Benzoic acid **88** was subjected to an amide coupling using HATU and amine **±114**. The resulting methyl sulfoxides (**±121**) were deprotonated using LDA and reacted with ethyl 2,2-difluorobutanoate. The synthetic routes and procedures for the synthesis of the intermediates are shown in the Supporting Information (Schemes S2–S7).

## Conclusions

Here, a ligand-based optimization of a novel chemotype for MAGL inhibitors was described. Compared with the original hit **1**, the MAGL inhibitory activity of **±43** increased around 100-fold. Importantly, by replacing the nitro group with chloro, the potential genotoxicity liability was removed. To optimize the metabolic stability of **±43,** three different strategies were employed: (1) reducing the lipophilicity; (2) applying steric hindrance; and (3) replacing the ester group with bioisosteres. The latter strategy provided compound **±73** (**LEI-515**), in which the ethyl ester group of compound **±43** was replaced by a difluoropropyl. Importantly, **LEI-515** has improved metabolic stability (Cl_int_ = 30 μL/min/mg) compared to compound **±43** (Cl_int_ > 346 μL/min/mg). Moreover, **LEI-515** had improved potency compared (pIC_50_ 9.30 ± 0.04) to compound **±43** (pIC_50_ 8.50 ± 0.10). Recently, we have described the selectivity profile and activity in live cells of **LEI-515**.^21^ Furthermore, **LEI-515** increased 2-AG levels in mouse colon and lung, but not in the brain (oral administration). Interestingly, **LEI-515** was able to reduce inflammation in an acute liver injury mouse model and suppressed chemotherapy-induced neuropathic pain in mice without inducing central nervous system adverse effects or physical dependence.^21^ We anticipate that **LEI-515** is a suitable tool compound to study the pathophysiological role of MAGL outside of the central nervous system. Moreover, **LEI-515** provides a starting point for future development of a novel class of anti-inflammatory analgesics with reduced side effects.

## Experimental Section

### Biological Procedures

#### MAGL Natural Substrate Assay

The MAGL activity assay is based on the production of glycerol from 2-arachidonoylglycerol (2-AG) hydrolysis by MAGL-overexpressing membrane preparations from transiently transfected HEK293T cells, as previously reported.^20^ The produced glycerol is coupled to the oxidation of commercially available AmplifuRed via a multienzyme cascade, resulting in a fluorescent signal from the dye resorufin. Standard assays were performed in HEMNB buffer (50 mM HEPES pH 7.4, 1 mM EDTA, 5 mM MgCl_2_, 100 mM NaCl, 0.5% (w/v) BSA) in black, flat bottom 96-wells plates. The final protein concentration of membrane preparations from overexpressing hMAGL HEK293T cells was 1.5 μg/mL (0.3 μg per well). Inhibitors were added from 40x concentrated DMSO stocks. After 20 min incubation, 100 μL assay mix containing glycerol kinase (GK), glycerol-3-phosphate oxidase (GPO), horse radish peroxidase (HRP), adenosine triphosphate (ATP), AmplifuRed, and 2-arachidonoylglycerol (2-AG) was added and fluorescence was measured in 5 min intervals for 60 min on a plate reader. Final assay concentrations were 0.2 U/mL GK, GPO, and HRP, 0.125 mM ATP, 10 μM AmplifuRed, 25 μM 2-AG, 5% DMSO, 0.5% ACN in a total volume of 200 μL. All measurements were performed in *N* = 2, *n* = 2 or *N* = 2, *n* = 4 for controls, with *Z*′ ≥ 0.6. For IC_50_ determination, the MAGL-overexpressing membranes were incubated with different inhibitor concentrations. Slopes of corrected fluorescence in time were determined in the linear interval of *t* = 10 to *t* = 35 min and then scaled to the corrected positive control of hMAGL-overexpressing membranes treated with vehicle (DMSO) as a 100% activity reference point. The data were exported to GraphPad Prism 5.0 and analyzed in a nonlinear dose–response analysis with variable slope.

#### Liver S9 Stability Assay

The rate of metabolism was assessed by incubation at 37 °C, pH 7.4 with mouse liver S9 fraction (1 mg protein/mL) supplemented with 5 mM NADP, 25 mM G6P, and 25 U/ml G6PD. The concentration of the initial substrate is 1 μM. Substrate depletion over a time course was measured by LC-MS/MS following protein precipitation. Amitryptilin (2.5 μM in ACN) was used as an internal standard. The ln peak area ratio (compound peak area/internal standard peak area) is plotted against time and the gradient of the line is determined. The following equations were used to calculate the intrinsic clearance:

Elimination rate constant (*k*) = (-gradient); half-life (*t*_1/2_, min) = 0.693/k; *V* (μL/mg) = volume of incubation (μL)/protein in the incubation (mg); intrinsic clearance (Cl_int_, μL/min/mg) = *V* × 0.693/*t*_1/2_.

### Chemistry Procedures

#### General

All reactions were performed using oven or flame-dried glassware and analytical-grade solvents that were dried with molecular sieves. Reagents were purchased from Sigma-Aldrich, Acros, and Merck and used without further purification unless noted otherwise. All moisture-sensitive reactions were performed under an argon or nitrogen atmosphere. Traces of water were removed from starting compounds by co-evaporation with toluene. Reactions were followed by thin-layer chromatography and were performed using TLC Silica gel 60 F_245_ on aluminum sheets. Compounds were visualized using a KMnO_4_ stain K_2_CO_3_ (66 mg/mL) and KMnO_4_ (10 mg/mL) in 0.1% NaOH. Amines were visualized using ninhydrin (0.75 g/L) and acetic acid (12.5 mL/L) in ethanol. ^1^H- and ^13^C NMR spectra were recorded on a Bruker AV-400, 500, 600, or 850 using CDCl_3_ or CD_3_OD as the solvent, unless stated otherwise. Chemical shift values are reported in ppm with tetramethylsilane or solvent resonance as the internal standard (CDCl_3_: δ 7.26 for ^1^H, δ 77.16 for ^13^C, CD_3_OD: δ 3.31 for ^1^H, δ 49.00 for ^13^C). Data are reported as follows: chemical shifts (δ) in ppm, multiplicity (s = singlet, d = doublet, dd = double doublet, td = triple doublet, t = triplet, q = quartet, quinted = quint, br = broad, m = multiplet), coupling constants *J* (Hz), and integration. LC-MS measurements were performed on a Thermo Finnigan LCQ Advantage Max ion-trap mass spectrometer (ESI^+^) coupled to a Surveyor HPLC system (Thermo Finnigan) equipped with a standard C18 (Gemini, 4.6 mmD × 50 mmL, 5 μm particle size, Phenomenex) analytical column and buffers A: H_2_O, B: ACN, C: 0.1% aq. TFA. Preparative HPLC purification was performed on a Waters Acquity Ultra Performance LC with a C18 column (Gemini, 150 × 21.2 mm, Phenomenex). Diode detection was done between 210 and 600 nm. Gradient: ACN in (H_2_O + 0.2% TFA). High-resolution mass spectra (HRMS) were recorded on a Thermo Scientific LTQ Orbitrap XL. All final compounds were determined to be >95% pure by integrating UV intensity recorded via HPLC. The synthetic procedures for all intermediates are provided in the Supporting Information.

#### General Procedure A

To a cooled solution of the appropriate carboxylic acid (1 equiv) in dried DCM (0.1 M) was subsequently added 2 drops of DMF and oxalyl chloride (1.2 equiv). Then, the mixture was allowed to warm to room temperature and continuously stirred for 2 h. The reaction progress was monitored by TLC analysis. Upon full conversion of the starting materials, the mixture was dropwise added to a cooled (0 °C) solution of the appropriate alcohol (3 equiv) or amine (3 equiv) and DiPEA (3 equiv) in DCM (0.1 M). Then the reaction mixture was stirred at room temperature overnight. The reaction progress was monitored by TLC analysis. Upon full conversion of the starting materials, the mixture was diluted with DCM and washed with water, dried (MgSO_4_), filtered, and concentrated under reduced pressure. The residue was purified by HPLC-MS.

#### General Procedure B

To a suspension or solution of the appropriate benzoic acid (1 equiv) in DCM (0.4 M) was added HATU (1.5 equiv) and DiPEA (3 equiv) and then the mixture was stirred at room temperature for 1 h. The appropriate phenylpiperazine (1 equiv) was added and the mixture was stirred overnight. The reaction progress was monitored by TLC analysis. Upon full conversion of the starting materials, the mixture was diluted with DCM and washed with water, dried (MgSO_4_), filtered, and concentrated under reduced pressure. The residue was purified by silica gel column chromatography (pentane/EtOAc) or HPLC-MS.

#### General Procedure C

To a solution of appropriate methyl sulfoxide (1 equiv) in anhydrous THF (10 mM) was added LDA (2 equiv) at −78 °C and the reaction mixture was stirred for 30 min before the appropriate ester (10 equiv) was added. The reaction progress was monitored by TLC analysis. Once completed, the mixture was quenched with NH_4_Cl solution, extracted with DCM, and dried over anhydrous MgSO_4_. After filtration, the filtrate was concentrated under reduced pressure. The residue was purified by silica gel column chromatography or prep-HPLC.

#### Ethyl 2-((4-((*R*)-3-Methyl-4-(*m*-tolyl)piperazine-1-carbonyl)-2-nitrophenyl)sulfinyl)acetate ((*R*)**-1**)

The title compound was synthesized using (*R*)-2-methyl-1-(*m*-tolyl)piperazine (57.4 mg, 0.30 mmol, 1 equiv), 4-((2-ethoxy-2-oxoethyl)sulfinyl)-3-nitrobenzoic acid (100 mg, 0.33 mmol, 1.1 equiv), HATU (140 mg, 0.45 mmol, 1.5 equiv), and DiPEA (117 mg, 0.91 mmol, 3 equiv) according to general procedure B in a yield of 98 mg (0.21 mmol, 69%). ^1^H NMR (850 MHz, CDCl_3_) δ 8.47–8.34 (m, 2H), 8.07–7.97 (m, 1H), 7.18 (t, *J* = 7.8 Hz, 1H), 6.83–6.68 (m, 3H), 4.47–4.08 (m, 4H), 3.78 (d, *J* = 13.8 Hz, 2H), 3.72–3.06 (m, 5H), 2.33 (s, 3H), 1.28 (td, *J* = 7.1, 1.7 Hz, 3H), 1.12–0.92 (m, 3H). ^13^C NMR (214 MHz, CDCl_3_) δ 166.88, 164.60, 149.51, 144.89, 143.86, 139.88, 139.17, 133.69, 129.20, 127.89, 124.24, 121.93, 119.36, 115.60, 62.44, 60.04, 52.25, 47.72, 45.62, 42.59, 21.79, 14.18, 12.71.

#### Ethyl 2-((4-((*S*)-3-Methyl-4-(*m*-tolyl)piperazine-1-carbonyl)-2-nitrophenyl)sulfinyl)acetate ((*S*)**-1**)

The title compound was synthesized using (*S*)-2-methyl-1-(*m*-tolyl)piperazine (57.4 mg, 0.30 mmol, 1 equiv), 4-((2-ethoxy-2-oxoethyl)sulfinyl)-3-nitrobenzoic acid (100 mg, 0.33 mmol, 1.1 equiv), HATU (140 mg, 0.45 mmol, 1.5 equiv), and DiPEA (117 mg, 0.91 mmol, 3 equiv) according to general procedure B in a yield of 140 mg (0.30 mmol, 98%).

#### Ethyl 2-((2-Nitro-4-(4-(*m*-tolyl)piperazine-1-carbonyl)phenyl)sulfinyl)acetate (**2**)

The title compound was synthesized using 1-(*m*-tolyl)piperazine (29.3 mg, 0.17 mmol, 1 equiv), 4-((2-ethoxy-2-oxoethyl)sulfinyl)-3-nitrobenzoic acid (50 mg, 0.17 mmol, 1 equiv), HATU (95 mg, 0.25 mmol, 1.5 equiv), and DiPEA (64.4 mg, 0.50 mmol, 3 equiv) according to general procedure B in a yield of 53.4 mg (0.12 mmol, 70%). ^1^H NMR (850 MHz, CDCl_3_) δ 8.44 (d, *J* = 1.7 Hz, 1H), 8.37 (d, *J* = 8.1 Hz, 1H), 8.07 (dd, *J* = 8.0, 1.6 Hz, 1H), 7.35 (t, *J* = 7.9 Hz, 1H), 7.24–7.17 (m, 2H), 7.15 (dd, *J* = 7.5, 1.5 Hz, 1H), 4.32–4.08 (m, 5H), 3.90 (m, 2H), 3.77 (d, *J* = 14.2 Hz, 1H), 3.54 (m, 4H), 2.39 (s, 3H), 1.26 (t, *J* = 7.7 Hz, 3H). ^13^C NMR (214 MHz, CDCl_3_) δ 166.90, 164.84, 144.96, 144.18, 143.94, 140.85, 138.59, 133.88, 130.21, 128.65, 128.02, 124.49, 120.28, 116.68, 62.62, 59.96, 53.48, 53.30, 45.78, 40.63, 21.56, 14.08.

#### Ethyl (*S*)-2-((4-(3-Methyl-4-(*m*-tolyl)piperazine-1-carbonyl)-2-nitrophenyl)thio)acetate (**3**)

The title compound was synthesized using (*R*)-2-methyl-1-(*m*-tolyl)piperazine (60.6 mg, 0.32 mmol, 1 equiv), 4-((2-ethoxy-2-oxoethyl)thio)-3-nitrobenzoic acid (100 mg, 0.35 mmol, 1.1 equiv), HATU (147 mg, 0.48 mmol, 1.5 equiv), and DiPEA (124 mg, 0.96 mmol, 3 equiv) according to general procedure B. This yielded the product (108 mg, 0.24 mmol, 74%). ^1^H NMR (400 MHz, CDCl_3_) δ 8.34 (d, *J* = 1.9 Hz, 1H), 7.70 (dd, *J* = 8.4, 1.9 Hz, 1H), 7.60 (d, *J* = 8.4 Hz, 1H), 7.17 (t, *J* = 7.9 Hz, 1H), 6.75 (d, *J* = 5.8 Hz, 3H), 4.49–3.01 (m, 7H), 4.24 (q, *J* = 7.1 Hz, 2H), 3.79 (s, 2H), 2.32 (s, 3H), 1.29 (t, *J* = 7.1 Hz, 3H), 1.01 (m, 3H). ^13^C NMR (101 MHz, CDCl_3_) δ 168.26, 167.70, 149.38, 144.90, 138.78, 138.48, 132.42, 132.36, 128.85, 126.69, 124.90, 121.48, 118.91, 115.14, 61.98, 52.72, 51.87, 47.65, 45.06, 34.72, 21.49, 13.86, 12.47. HRMS: Calcd for [C_23_H_27_N_3_O_5_S + H]^+^ = 458.1744, found = 458.1743.

#### Ethyl (*S*)-2-((4-(3-Methyl-4-(*m*-tolyl)piperazine-1-carbonyl)-2-nitrophenyl)sulfonyl)acetate (**4**)

The title compound was synthesized using (*S*)-2-methyl-1-(*m*-tolyl)piperazine (12 mg, 0.06 mmol, 1 equiv), 4-((2-ethoxy-2-oxoethyl)sulfonyl)-3-nitrobenzoic acid (20 mg, 0.06 mmol, 1 equiv), HATU (36 mg, 0.10 mmol, 1.5 equiv), and DiPEA (24 mg, 0.19 mmol, 3 equiv) according to general procedure B in a yield of 2 mg (0.004 mmol, 6%). ^1^H NMR (400 MHz, CDCl_3_) δ 8.30 (d, *J* = 8.0 Hz, 1H), 7.94 (d, *J* = 3.9 Hz, 1H), 7.83 (d, *J* = 8.0 Hz, 1H), 7.18 (t, *J* = 7.6 Hz, 1H), 6.76 (s, 3H), 4.68 (s, 2H), 4.45–3.05 (m, 7H), 4.22 (q, *J* = 7.1 Hz, 2H), 2.33 (s, 3H), 1.27 (t, *J* = 7.0 Hz, 4H), 1.02 (m, 3H).

#### Ethyl 2-((4-((*S*)-3-Methyl-4-(*m*-tolyl)piperazine-1-carbonyl)phenyl)sulfinyl)acetate (**5**)

The title compound was synthesized using (*S*)-2-methyl-1-(*m*-tolyl)piperazine (22 mg, 0.12 mmol, 1 equiv), 4-((2-ethoxy-2-oxoethyl)sulfinyl)benzoic acid (30 mg, 0.12 mmol, 1 equiv), oxalyl chloride (16 mg, 0.13 mmol, 1.1 equiv), and DiPEA (45 mg, 0.35 mmol, 3 equiv) according to general procedure A in a yield of 21 mg (0.05 mmol, 42%). ^1^H NMR (400 MHz, CDCl_3_) δ 8.24 (d, *J* = 8.5 Hz, 2H), 7.79 (d, *J* = 8.5 Hz, 2H), 7.18 (t, *J* = 7.8 Hz, 1H), 6.82–6.67 (m, 3H), 4.46–4.05 (m, 4H), 3.78 (d, *J* = 13.8 Hz, 2H), 3.70–3.04 (m, 5H), 2.31 (s, 3H), 1.24 (td, *J* = 7.1, 1.7 Hz, 3H), 1.11–0.92 (m, 3H).

#### Ethyl 2-((2-Nitro-4-(4-phenylpiperazine-1-carbonyl)phenyl)sulfinyl)acetate (**6**)

The title compound was synthesized using 1-phenylpiperazine (25 mg, 0.16 mmol, 1 equiv), 4-((2-ethoxy-2-oxoethyl)sulfinyl)-3-nitrobenzoic acid (70 mg, 0.23 mmol, 1.5 equiv), HATU (140 mg, 0.37 mmol, 2.3 equiv), and DiPEA (65 μL, 0.37 mmol, 2.3 equiv) according to general procedure B in a yield of 64 mg (0.14 mmol, 89%). ^1^H NMR (400 MHz, CDCl_3_) δ 8.43–8.37 (m, 2H), 8.01 (dd, *J* = 8.0, 1.6 Hz, 1H), 7.34–7.25 (m, 2H), 6.98–6.91 (m, 3H), 4.28–4.17 (m, 2H), 4.14 (d, *J* = 13.7 Hz, 1H), 3.98 (s, 2H), 3.77 (d, *J* = 13.7 Hz, 1H), 3.59 (s, 2H), 3.31 (s, 2H), 3.18 (s, 2H), 1.28 (t, *J* = 7.1 Hz, 3H). ^13^C NMR (101 MHz, CDCl_3_) δ 166.54, 164.58, 150.65, 144.91, 143.98, 139.79, 133.72, 129.41, 127.93, 124.22, 121.13, 117.00, 62.44, 60.08, 50.12, 49.62, 47.82, 42.51, 38.68, 14.18. HRMS: Calcd for [C_21_H_23_N_3_O_6_S+H]^+^ = 446.1380, found = 446.1379.

#### Ethyl 2-((4-(4-(3-Fluorophenyl)piperazine-1-carbonyl)-2-nitrophenyl)sulfinyl)acetate (**7**)

The title compound was synthesized using 1-(3-fluorophenyl)piperazine (18 mg, 0.10 mmol, 1 equiv), 4-((2-ethoxy-2-oxoethyl)sulfinyl)-3-nitrobenzoic acid (30 mg, 0.12 mmol, 1 equiv), oxalyl chloride (13.90 mg, 0.11 mmol, 1.1 equiv), and DiPEA (39 mg, 0.30 mmol, 3 equiv) according to general procedure A in a yield of 30 mg (0.07 mmol, 42%). ^1^H NMR (400 MHz, CDCl_3_) δ 8.41 (dd, *J* = 4.8, 3.2 Hz, 2H), 8.02 (dd, *J* = 8.1, 1.6 Hz, 1H), 7.23 (ddd, *J* = 8.3, 6.6, 1.4 Hz, 1H), 6.77–6.66 (m, 1H), 6.65–6.56 (m, 2H), 4.26–4.18 (m, 2H), 4.14 (d, *J* = 13.7 Hz, 1H), 3.98 (s, 2H), 3.78 (d, *J* = 13.6 Hz, 1H), 3.59 (s, 2H), 3.32–3.20 (m, 4H), 1.29 (t, *J* = 7.1 Hz, 3H). ^13^C NMR (101 MHz, CDCl_3_) δ 166.63, 165.09, 163.63 (d, *J* = 196.4 Hz), 152.26 (d, *J* = 9.6 Hz), 144.98, 144.18, 139.69, 133.76, 130.58 (d, *J* = 9.8 Hz), 128.06, 124.28, 112.09 (d, *J* = 2.4 Hz), 107.46 (d, *J* = 21.3 Hz), 103.81 (d, *J* = 24.9 Hz), 62.53, 60.12, 49.55, 42.32, 14.24.

#### Ethyl 2-((4-(4-(3-Chlorophenyl)piperazine-1-carbonyl)-2-nitrophenyl)sulfinyl)acetate (**8**)

The title compound was synthesized using 1-(3-chlorophenyl)piperazine (20 mg, 0.10 mmol, 1 equiv), 4-((2-ethoxy-2-oxoethyl)sulfinyl)-3-nitrobenzoic acid (30 mg, 0.12 mmol, 1 equiv), oxalyl chloride (13.90 mg, 0.11 mmol, 1.1 equiv), and DiPEA (39 mg, 0.30 mmol, 3 equiv) according to general procedure A in a yield of 28 mg (0.06 mmol, 57%). ^1^H NMR (400 MHz, CDCl_3_) δ 8.51–8.31 (m, 2H), 8.01 (dd, *J* = 8.0, 1.6 Hz, 1H), 7.21 (t, *J* = 8.4 Hz, 1H), 6.92–6.86 (m, 2H), 6.80 (ddd, *J* = 8.3, 2.3, 1.0 Hz, 1H), 4.30–4.16 (m, 2H), 4.13 (d, *J* = 13.7 Hz, 1H), 3.97 (s, 2H), 3.78 (d, *J* = 13.7 Hz, 1H), 3.59 (s, 2H), 3.31–3.19 (m, 4H), 1.33–1.19 (t, *J* = 7.2, 3H). ^13^C NMR (101 MHz, CDCl_3_) δ 166.62, 164.58, 151.75, 144.98, 144.18, 139.69, 135.24, 133.74, 130.40, 128.04, 124.25, 120.83, 116.86, 114.87, 62.51, 60.13, 49.58, 49.18, 47.62, 42.38, 14.24.

#### Ethyl 2-((4-(4-(4-Chlorophenyl)piperazine-1-carbonyl)-2-nitrophenyl)sulfinyl)acetate (**9**)

The title compound was synthesized using 1-(4-chlorophenyl)piperazine (19 mg, 0.10 mmol, 1 equiv), 4-((2-ethoxy-2-oxoethyl)sulfinyl)-3-nitrobenzoic acid (30 mg, 0.12 mmol, 1 equiv), oxalyl chloride (13.90 mg, 0.11 mmol, 1.1 equiv), and DiPEA (39 mg, 0.30 mmol, 3 equiv) according to general procedure A in a yield of 8 mg (0.02 mmol, 18%). ^1^H NMR (850 MHz, CDCl_3_) δ 8.47–8.36 (m, 2H), 8.02 (dd, *J* = 8.0, 1.6 Hz, 1H), 7.30–7.27 (m, 2H), 6.97 (m, 2H), 4.26–4.18 (m, 2H), 4.14 (d, *J* = 13.8 Hz, 1H), 4.04 (m, 2H), 3.79 (d, *J* = 13.8 Hz, 1H), 3.65 (m, 2H), 3.38–3.11 (m, 4H), 1.29 (t, *J* = 7.2 Hz, 3H). ^13^C NMR (214 MHz, CDCl_3_) δ 166.66, 164.61, 148.42, 145.00, 144.25, 139.55, 133.78, 129.55, 128.12, 127.21, 124.30, 118.79, 62.57, 60.09, 50.68, 50.15, 47.41, 42.17, 14.26.

#### Ethyl 2-((4-(4-(2-Chlorophenyl)piperazine-1-carbonyl)-2-nitrophenyl)sulfinyl)acetate (**10**)

The title compound was synthesized using 1-(2-chlorophenyl)piperazine (26 mg, 0.13 mmol, 1 equiv), 4-((2-ethoxy-2-oxoethyl)sulfinyl)-3-nitrobenzoic acid (40 mg, 0.13 mmol, 1 equiv), oxalyl chloride (19 mg, 0.15 mmol, 1.1 equiv), and DiPEA (52 mg, 0.40 mmol, 3 equiv) according to general procedure A in a yield of 41 mg (0.09 mmol, 64%). ^1^H NMR (400 MHz, CDCl_3_) δ 8.54–8.26 (m, 2H), 8.03 (dd, *J* = 8.0, 1.6 Hz, 1H), 7.39 (dd, *J* = 8.2, 1.5 Hz, 1H), 7.29–7.22 (m, 1H), 7.11–6.94 (m, 2H), 4.22 (qd, *J* = 7.2, 3.9 Hz, 2H), 4.14 (d, *J* = 13.7 Hz, 1H), 4.02 (s, 2H), 3.78 (d, *J* = 13.7 Hz, 1H), 3.62 (s, 2H), 3.17–3.05 (m, 4H), 1.28 (t, *J* = 7.1 Hz, 3H). ^13^C NMR (101 MHz, CDCl_3_) δ 166.68, 164.61, 148.28, 144.93, 143.95, 139.99, 133.76, 130.91, 129.08, 127.97, 127.90, 124.77, 124.24, 120.73, 62.49, 60.12, 51.74, 50.99, 48.22, 42.87, 14.22. HRMS: Calcd for [C_21_H_22_ClN_3_O_6_S + H]^+^ = 480.0991, found = 480.0991.

#### Ethyl 2-((4-(4-(3-Bromophenyl)piperazine-1-carbonyl)-2-nitrophenyl)sulfinyl)acetate (**11**)

The title compound was synthesized using 1-(3-bromophenyl)piperazine (23 mg, 0.10 mmol, 1 equiv), 4-((2-ethoxy-2-oxoethyl)sulfinyl)-3-nitrobenzoic acid (30 mg, 0.10 mmol, 1 equiv), oxalyl chloride (13.90 mg, 0.11 mmol, 1.1 equiv), and DiPEA (39 mg, 0.30 mmol, 3 equiv) according to general procedure A in a yield of 26 mg (0.05 mmol, 51%). ^1^H NMR (500 MHz, CDCl_3_) δ 8.48–8.34 (m, 2H), 8.01 (dd, *J* = 8.1, 1.6 Hz, 1H), 7.15 (dd, *J* = 8.6, 7.4 Hz, 1H), 7.05 (dd, *J* = 8.0, 1.2 Hz, 2H), 6.90–6.78 (m, 1H), 4.28–4.18 (m, 2H), 4.14 (d, *J* = 13.7 Hz, 1H), 3.97 (s, 2H), 3.79 (d, *J* = 13.7 Hz, 1H), 3.70–3.02 (m, 6H), 1.29 (t, *J* = 7.1 Hz, 3H). ^13^C NMR (126 MHz, CDCl_3_) δ 166.66, 164.60, 151.93, 145.01, 144.21, 139.70, 133.76, 130.71, 128.09, 124.28, 123.85, 123.47, 119.85, 115.43, 62.55, 60.13, 49.65, 49.20, 47.65, 42.42, 14.27.

#### Ethyl 2-((4-(4-(4-Bromophenyl)piperazine-1-carbonyl)-2-nitrophenyl)sulfinyl)acetate (**12**)

The title compound was synthesized using 1-(4-bromophenyl)piperazine (23 mg, 0.10 mmol, 1 equiv), 4-((2-ethoxy-2-oxoethyl)sulfinyl)-3-nitrobenzoic acid (30 mg, 0.10 mmol, 1 equiv), oxalyl chloride (13.90 mg, 0.11 mmol, 1.1 equiv), and DiPEA (39 mg, 0.30 mmol, 3 equiv) according to general procedure A in a yield of 31 mg (0.06 mmol, 61%). ^1^H NMR (850 MHz, CDCl_3_) δ 8.42 (dd, *J* = 4.8, 3.1 Hz, 2H), 8.02 (dd, *J* = 8.0, 1.6 Hz, 1H), 7.41–7.39 (m, 2H), 6.85 (d, *J* = 8.4 Hz, 2H), 4.27–4.17 (m, 2H), 4.14 (d, *J* = 13.7 Hz, 1H), 4.00 (m, 2H), 3.79 (d, *J* = 13.8 Hz, 1H), 3.61 (m, 2H), 3.34–3.09 (m, 4H), 1.29 (t, *J* = 7.2 Hz, 3H). ^13^C NMR (214 MHz, CDCl_3_) δ 166.65, 164.61, 149.31, 145.00, 144.22, 139.63, 133.78, 132.40, 128.11, 124.30, 118.91, 62.57, 60.10, 50.31, 49.75, 47.57, 42.25, 14.26.

#### Ethyl 2-((2-Nitro-4-(4-(3-(trifluoromethyl)phenyl)piperazine-1-carbonyl)phenyl)sulfinyl)acetate (**13**)

The title compound was synthesized using 1-(3-(trifluoromethyl)phenyl)piperazine (31 mg, 0.13 mmol, 1 equiv), 4-((2-ethoxy-2-oxoethyl)sulfinyl)-3-nitrobenzoic acid (40 mg, 0.13 mmol, 1 equiv), oxalyl chloride (19 mg, 0.15 mmol, 1.1 equiv), and DiPEA (52 mg, 0.40 mmol, 3 equiv) according to general procedure A in a yield of 42 mg (0.08 mmol, 62%). ^1^H NMR (400 MHz, CDCl_3_) δ 8.55–8.29 (m, 2H), 8.02 (dd, *J* = 8.1, 1.6 Hz, 1H), 7.40 (t, *J* = 8.0 Hz, 1H), 7.21–7.07 (m, 3H), 4.26–4.18 (m, 2H), 4.14 (d, *J* = 13.7 Hz, 1H), 4.00 (br, 2H), 3.79 (d, *J* = 13.7 Hz, 1H), 3.62 (br, 2H), 3.30 (br, 4H), 1.29 (t, *J* = 7.1 Hz, 3H). ^13^C NMR (101 MHz, CDCl_3_) δ 166.67, 164.60, 150.87, 145.00, 144.23, 139.65, 133.76, 131.81 (q, *J* = 32.3 Hz), 129.99, 128.09, 124.28, 122.85(q, *J* = 273.71 Hz) 119.87, 117.48 (q, *J* = 4.04 Hz), 113.28 (q, *J* = 3.03 Hz), 62.54, 60.11, 49.57, 47.57, 14.25. HRMS: Calcd for [C_22_H_22_F_3_N_3_O_6_S + H]^+^ = 514.1254, found = 514.1252.

#### Ethyl 2-((2-Nitro-4-(4-(4-(trifluoromethyl)phenyl)piperazine-1-carbonyl)phenyl)sulfinyl)acetate (**14**)

The title compound was synthesized using 1-(4-(trifluoromethyl)phenyl)piperazine (31 mg, 0.13 mmol, 1 equiv), 4-((2-ethoxy-2-oxoethyl)sulfinyl)-3-nitrobenzoic acid (40 mg, 0.13 mmol, 1 equiv), oxalyl chloride (19 mg, 0.15 mmol, 1.1 equiv), and DiPEA (52 mg, 0.40 mmol, 3 equiv) according to general procedure A in a yield of 39 mg (0.08 mmol, 57%). ^1^H NMR (400 MHz, CDCl_3_) δ 8.53–8.29 (m, 2H), 8.02 (dd, *J* = 8.1, 1.6 Hz, 1H), 7.62–7.45 (m, 2H), 6.96 (d, *J* = 8.5 Hz, 2H), 4.22 (m, 2H), 4.14 (d, *J* = 13.7 Hz, 1H), 3.99 (br, 2H), 3.78 (d, *J* = 13.7 Hz, 1H), 3.62 (br, 2H), 3.35 (br, 4H), 1.28 (t, *J* = 7.2 Hz, 3H). ^13^C NMR (101 MHz, CDCl_3_) δ 166.57, 164.52, 152.66, 144.88, 144.14, 139.49, 133.67, 127.97, 126.62 (q, *J* = 3.75), 124.48(q, *J* = 271.94 Hz), 122.32 (q, *J* = 32.32 Hz) 124.19, 115.49, 62.44, 60.01, 48.84, 48.31, 47.51, 42.15, 14.14. HRMS: Calcd for [C_22_H_22_F_3_N_3_O_6_S + H]^+^ = 514.1254, found = 514.1253.

#### Ethyl 2-((4-(4-(3-Methoxyphenyl)piperazine-1-carbonyl)-2-nitrophenyl)sulfinyl)acetate (**15**)

The title compound was synthesized using 1-(3-methoxyphenyl)piperazine (19 mg, 0.10 mmol, 1 equiv), 4-((2-ethoxy-2-oxoethyl)sulfinyl)-3-nitrobenzoic acid (30 mg, 0.10 mmol, 1 equiv), oxalyl chloride (14 mg, 0.11 mmol, 1.1 equiv), and DiPEA (39 mg, 0.30 mmol, 3 equiv) according to general procedure A in a yield of 29 mg (0.06 mmol, 55%). ^1^H NMR (850 MHz, CDCl_3_) δ 8.43–8.39 (m, 2H), 8.02 (dd, *J* = 8.0, 1.7 Hz, 1H), 7.23 (t, *J* = 8.2 Hz, 1H), 6.68–6.45 (m, 3H), 4.26–4.17 (m, 2H), 4.14 (d, *J* = 13.8 Hz, 1H), 4.01 (m, 2H), 3.81 (s, 3H), 3.78 (d, *J* = 13.8 Hz, 1H), 3.60 (m, 2H), 3.39–3.15 (m, 4H), 1.28 (t, *J* = 7.2 Hz, 3H). ^13^C NMR (214 MHz, CDCl_3_) δ 166.51, 164.53, 160.67, 151.51, 144.87, 143.99, 139.62, 133.69, 130.19, 127.94, 124.21, 109.75, 106.14, 103.75, 62.44, 60.01, 55.32, 50.26, 49.76, 47.49, 42.20, 14.14.

#### Ethyl 2-((4-(4-(4-Methoxyphenyl)piperazine-1-carbonyl)-2-nitrophenyl)sulfinyl)acetate (**16**)

The title compound was synthesized using 1-(4-methoxyphenyl)piperazine (32 mg, 0.17 mmol, 1 equiv), 4-((2-ethoxy-2-oxoethyl)sulfonyl)-3-nitrobenzoic acid (50 mg, 0.17 mmol, 1 equiv), HATU (95 mg, 0.25 mmol, 1.5 equiv), and DiPEA (64 mg, 0.50 mmol, 3 equiv) according to general procedure B in a yield of 71 mg (0.15 mmol, 90%). ^1^H NMR (850 MHz, CDCl_3_) δ 8.41 (d, *J* = 1.6 Hz, 1H), 8.40 (d, *J* = 8.1 Hz, 1H), 8.02 (dd, *J* = 8.0, 1.6 Hz, 1H), 6.94 (d, *J* = 8.7 Hz, 2H), 6.89–6.82 (m, 2H), 4.26–4.16 (m, 2H), 4.14 (d, *J* = 13.9 Hz, 1H), 3.99 (m, 2H), 3.81–3.74 (m, 4H), 3.59 (m, 2H), 3.23–2.98 (m, 4H), 1.28 (t, *J* = 7.6 Hz, 3H). ^13^C NMR (214 MHz, CDCl_3_) δ 166.50, 164.61, 154.84, 144.87, 144.65, 143.88, 139.82, 133.73, 127.89, 124.22, 119.31, 114.63, 62.45, 60.06, 55.60, 51.59, 51.04, 47.89, 42.56, 14.17.

#### Ethyl 2-((2-Nitro-4-(4-(3-nitrophenyl)piperazine-1-carbonyl)phenyl)sulfinyl)acetate (**17**)

The title compound was synthesized using 1-(3-nitrophenyl)piperazine (21 mg, 0.10 mmol, 1 equiv), 4-((2-ethoxy-2-oxoethyl)sulfinyl)-3-nitrobenzoic acid (30 mg, 0.10 mmol, 1 equiv), oxalyl chloride (14 mg, 0.11 mmol, 1.1 equiv), and DiPEA (39 mg, 0.30 mmol, 3 equiv) according to general procedure A in a yield of 29 mg (0.05 mmol, 45%). ^1^H NMR (850 MHz, CDCl_3_) δ 8.44–8.40 (m, 2H), 8.04 (dd, *J* = 8.0, 1.6 Hz, 1H), 7.76–7.73 (m, 2H), 7.46–7.42 (m, 1H), 7.24 (ddd, *J* = 8.3, 2.5, 1.0 Hz, 1H), 4.26–4.18 (m, 2H), 4.15 (d, *J* = 13.8 Hz, 1H), 4.02 (M, 2H), 3.79 (d, *J* = 13.8 Hz, 1H), 3.71–3.60 (m, 2H), 3.50–3.25 (m, 4H), 1.29 (t, *J* = 7.2 Hz, 3H). ^13^C NMR (214 MHz, CDCl_3_) δ 166.69, 164.63, 151.23, 149.29, 144.95, 144.20, 139.47, 133.76, 130.15, 128.05, 124.28, 122.14, 115.19, 110.69, 62.52, 60.08, 49.17, 48.70, 47.39, 42.20, 14.21.

#### Ethyl 2-((4-(4-([1,1′-Biphenyl]-3-yl)piperazine-1-carbonyl)-2-nitrophenyl)sulfinyl)acetate (**18**)

The title compound was synthesized using 1-([1,1′-biphenyl]-3-yl)piperazine (32 mg, 0.13 mmol, 1 equiv), 4-((2-ethoxy-2-oxoethyl)sulfinyl)-3-nitrobenzoic acid (40 mg, 0.13 mmol, 1 equiv), oxalyl chloride (19 mg, 0.15 mmol, 1.1 equiv), and DiPEA (52 mg, 0.4 mmol, 3 equiv) according to general procedure A in a yield of 40 mg (0.08 mmol, 58%). ^1^H NMR (400 MHz, CDCl_3_) δ 8.56–8.26 (m, 2H), 8.02 (dd, *J* = 8.0, 1.6 Hz, 1H), 7.59–7.54 (m, 2H), 7.48–7.41 (m, 2H), 7.40–7.33 (m, 2H), 7.21–7.13 (m, 2H), 7.01–6.86 (m, 1H), 4.22 (qq, J = 7.4, 3.6 Hz, 2H), 4.14 (d, J = 13.7 Hz, 1H), 4.01 (br, 2H), 3.78 (d, *J* = 13.7 Hz, 1H), 3.62 (br, 2H), 3.31 (br, 4H), 1.28 (t, *J* = 7.2 Hz, 3H). ^13^C NMR (101 MHz, CDCl_3_) δ 166.62, 164.61, 151.12, 144.98, 144.09, 142.73, 141.42, 139.83, 133.77, 129.84, 128.88, 128.03, 127.60, 127.33, 124.29, 120.38, 116.16, 116.03, 62.52, 60.13, 50.26, 49.85, 47.82, 42.54, 14.25. HRMS: Calcd for [C_27_H_27_N_3_O_6_S + H]^+^ = 522.1693, found = 522.1690.

#### Ethyl 2-((4-(4-(3,5-Dichlorophenyl)piperazine-1-carbonyl)-2-nitrophenyl)sulfinyl)acetate (**19**)

The title compound was synthesized using 1-(3,5-dichlorophenyl)piperazine (23 mg, 0.10 mmol, 1 equiv), 4-((2-ethoxy-2-oxoethyl)sulfinyl)-3-nitrobenzoic acid (30 mg, 0.10 mmol, 1 equiv), oxalyl chloride (14 mg, 0.11 mmol, 1.1 equiv), and DiPEA (39 mg, 0.3 mmol, 3 equiv) according to general procedure A in a yield of 12 mg (0.02 mmol, 23%). ^1^H NMR (850 MHz, CDCl_3_) δ 8.43–8.40 (m, 2H), 8.02 (dd, *J* = 8.0, 1.6 Hz, 1H), 6.90 (t, *J* = 1.7 Hz, 1H), 6.79 (d, *J* = 1.7 Hz, 2H), 4.26–4.18 (m, 2H), 4.14 (d, *J* = 13.8 Hz, 1H), 3.97 (m, 2H), 3.79 (d, *J* = 13.8 Hz, 1H), 3.60 (m, 2H), 3.27 (m, 4H), 1.29 (t, *J* = 7.2 Hz, 3H). ^13^C NMR (214 MHz, CDCl_3_) δ 166.67, 164.61, 151.92, 144.97, 144.23, 139.47, 135.80, 133.75, 128.08, 124.28, 120.56, 114.91, 62.54, 60.08, 49.16, 48.70, 47.34, 42.16, 14.23.

#### Ethyl 2-((4-(4-(3,4-Dichlorophenyl)piperazine-1-carbonyl)-2-nitrophenyl)sulfinyl)acetate (**20**)

The title compound was synthesized using 1-(3,4-dichlorophenyl)piperazine (23 mg, 0.10 mmol, 1 equiv), 4-((2-ethoxy-2-oxoethyl)sulfinyl)-3-nitrobenzoic acid (30 mg, 0.10 mmol, 1 equiv), oxalyl chloride (14 mg, 0.11 mmol, 1.1 equiv), and DiPEA (39 mg, 0.3 mmol, 3 equiv) according to general procedure A in a yield of 36 mg (0.07 mmol, 72%). ^1^H NMR (500 MHz, CDCl_3_) δ 8.50–8.33 (m, 2H), 8.02 (dd, *J* = 8.1, 1.6 Hz, 1H), 7.41 (d, *J* = 2.4 Hz, 1H), 7.23 (dd, *J* = 8.6, 2.4 Hz, 1H), 6.96 (d, *J* = 8.6 Hz, 1H), 4.28–4.16 (m, 2H), 4.14 (d, *J* = 13.6 Hz, 1H), 4.01 (s, 2H), 3.78 (d, *J* = 13.7 Hz, 1H), 3.61 (s, 2H), 3.08 (br, 4H), 1.28 (t, *J* = 7.1 Hz, 3H). 13C NMR (126 MHz, CDCl_3_) δ 166.70, 164.56, 147.05, 144.95, 144.06, 139.86, 133.73, 130.63, 129.85, 129.45, 128.03, 127.95, 124.21, 121.49, 62.50, 60.09, 51.70, 50.99, 48.13, 42.74, 14.21.

#### Ethyl 2-((4-(4-(2,4-Dichlorophenyl)piperazine-1-carbonyl)-2-nitrophenyl)sulfinyl)acetate (**21**)

The title compound was synthesized using 1-(2,4-dichlorophenyl)piperazine (23 mg, 0.10 mmol, 1 equiv), 4-((2-ethoxy-2-oxoethyl)sulfonyl)-3-nitrobenzoic acid (30 mg, 0.10 mmol, 1 equiv), HATU (57 mg, 0.15 mmol, 1.5 equiv), and DiPEA (39 mg, 0.30 mmol, 3 equiv) according to general procedure B in a yield of 41 mg (0.08 mmol, 84%). ^1^H NMR (400 MHz, CDCl_3_) δ 8.44–8.37 (m, 2H), 8.02 (dd, *J* = 8.0, 1.6 Hz, 1H), 7.40 (d, *J* = 2.4 Hz, 1H), 7.23 (dd, *J* = 8.6, 2.4 Hz, 1H), 6.97 (d, *J* = 8.7 Hz, 1H), 4.28–4.15 (m, 2H), 4.14 (d, *J* = 13.7 Hz, 1H), 4.00 (s, 2H), 3.78 (d, *J* = 13.7 Hz, 1H), 3.61 (s, 2H), 3.15 (s, 2H), 3.01 (s, 2H), 1.28 (t, *J* = 7.1 Hz, 3H). ^13^C NMR (101 MHz, CDCl_3_) δ 166.66, 164.58, 147.05, 144.91, 143.99, 139.83, 133.72, 130.57, 129.79, 129.35, 127.96, 127.92, 124.20, 121.50, 62.45, 60.08, 50.93, 48.10, 14.19. HRMS: Calcd for [C_21_H_21_Cl_2_N_3_O_6_S + H]^+^ = 514.0601, found = 514.0602.

#### Ethyl 2-((4-(4-(2,6-Dichlorophenyl)piperazine-1-carbonyl)-2-nitrophenyl)sulfinyl)acetate (**22**)

The title compound was synthesized using 1-(2,6-dichlorophenyl)piperazine (20 mg, 0.09 mmol, 1 equiv), 4-((2-ethoxy-2-oxoethyl)sulfonyl)-3-nitrobenzoic acid (39 mg, 0.14 mmol, 1.5 equiv), HATU (53 mg, 0.14 mmol, 1.5 equiv), and DiPEA (38 mg, 0.30 mmol, 3 equiv) according to general procedure B in a yield of 63 mg (0.08 mmol, 89%). ^1^H NMR (400 MHz, CDCl_3_) δ 8.43–8.37 (m, 2H), 8.01 (dd, *J* = 8.0, 1.6 Hz, 1H), 6.98–6.91 (m, 3H), 4.28–4.17 (m, 2H), 4.14 (d, *J* = 13.7 Hz, 1H), 3.98 (s, 2H), 3.77 (d, *J* = 13.7 Hz, 1H), 3.59 (s, 2H), 3.31 (s, 2H), 3.18 (s, 2H), 1.28 (t, *J* = 7.1 Hz, 3H). 13C NMR (101 MHz, CDCl_3_) δ 166.54, 164.58, 150.65, 144.91, 143.98, 139.79, 133.72, 129.41, 127.93, 124.22, 121.13, 117.00, 62.44, 60.08, 50.12, 49.62, 47.82, 42.51, 38.68, 14.18. HRMS: Calcd for [C_21_H_21_Cl_2_N_3_O_6_S + H]^+^ = 514.0601, found = 514.0600.

#### Ethyl 2-((4-((*S*)-4-(3-Fluorophenyl)-3-methylpiperazine-1-carbonyl)-2-nitrophenyl)sulfinyl)acetate (**23**)

The title compound was synthesized using (*S*)-1-(3-fluorophenyl)-2-methylpiperazine (26 mg, 0.13 mmol, 1 equiv), 4-((2-ethoxy-2-oxoethyl)sulfinyl)-3-nitrobenzoic acid (40 mg, 0.13 mmol, 1 equiv), oxalyl chloride (19 mg, 0.15 mmol, 1.1 equiv), and DiPEA (52 mg, 0.40 mmol, 3 equiv) according to general procedure A in a yield of 38 mg (0.08 mmol, 60%). ^1^H NMR (400 MHz, CDCl_3_) δ 8.60–8.27 (m, 2H), 8.06–7.91 (m, 1H), 7.22 (td, *J* = 8.4, 6.8 Hz, 1H), 6.76–6.46 (m, 3H), 4.47 (m, 1H), 4.21 (m, 2H), 4.14 (d, *J* = 13.7 Hz, 1H), 3.94 (m, 1H), 3.78 (d, *J* = 13.7 Hz, 1H), 3.74–2.99 (m, 5H), 1.29 (t, *J* = 7.1 Hz, 3H), 1.08 (m, 3H). ^13^C NMR (101 MHz, CDCl_3_) δ 167.37, 163.97 (d, *J* = 244.1 Hz), 164.59, 151.16 (d, *J* = 9.5 Hz), 144.96, 144.05, 139.73, 133.74, 130.55 (d, *J* = 9.9 Hz), 128.02, 124.22, 112.38 (d, *J* = 2.4 Hz), 106.99(d, *J* = 21.3 Hz), 104.00(d, *J* = 24.9 Hz), 62.50, 60.09, 52.53, 51.68, 47.39, 42.43, 14.23, 12.52. HRMS: Calcd for [C_22_H_24_FN_3_O_6_S + H]^+^ = 478.1443, found = 478.1440.

#### Ethyl 2-((4-((*R*)-4-(3-Chlorophenyl)-3-methylpiperazine-1-carbonyl)-2-nitrophenyl)sulfinyl)acetate ((*R*)**-24**)

The title compound was synthesized using (*R*)-1-(3-fluorophenyl)-2-methylpiperazine (28 mg, 0.13 mmol, 1 equiv), 4-((2-ethoxy-2-oxoethyl)sulfinyl)-3-nitrobenzoic acid (40 mg, 0.13 mmol, 1 equiv), oxalyl chloride (19 mg, 0.15 mmol, 1.1 equiv), and DiPEA (52 mg, 0.40 mmol, 3 equiv) according to general procedure A in a yield of 22 mg (0.05 mmol, 34%). ^1^H NMR (400 MHz, CDCl_3_) δ 8.43–8.38 (m, 2H), 8.11–7.94 (m, 1H), 7.20 (t, *J* = 8.3 Hz, 1H), 6.90–6.74 (m, 3H), 4.63–4.27 (m, 1H), 4.22 (m, 2H), 4.14 (d, *J* = 13.7 Hz, 1H), 3.92 (m, 1H), 3.78 (d, *J* = 13.7 Hz, 1H), 3.74–3.00 (m, 5H), 1.28 (t, *J* = 7.1 Hz, 3H), 1.06 (m, 3H). ^13^C NMR (101 MHz, CDCl_3_) δ 167.35, 164.59, 150.62, 144.95, 144.04, 139.71, 135.24, 133.73, 130.40, 128.01, 124.23, 120.43, 117.19, 115.18, 62.50, 60.10, 52.61, 51.81, 47.61, 42.41, 14.23, 12.63. HRMS: Calcd for [C_22_H_24_ClN_3_O_6_S + H]^+^ = 494.1147, found = 494.1142.

#### Ethyl 2-((4-((*S*)-4-(3-Chlorophenyl)-3-methylpiperazine-1-carbonyl)-2-nitrophenyl)sulfinyl)acetate ((*S*)**-24**)

The title compound was synthesized using (*S*)-1-(3-fluorophenyl)-2-methylpiperazine (28 mg, 0.13 mmol, 1 equiv), 4-((2-ethoxy-2-oxoethyl)sulfinyl)-3-nitrobenzoic acid (40 mg, 0.13 mmol, 1 equiv), oxalyl chloride (19 mg, 0.15 mmol, 1.1 equiv), and DiPEA (52 mg, 0.40 mmol, 3 equiv) according to general procedure A in a yield of 50 mg (0.10 mmol, 76%). ^1^H NMR (600 MHz, CDCl_3_) δ 8.51–8.33 (m, 2H), 8.04 (dd, *J* = 8.0, 1.6 Hz, 1H), 7.24 (d, *J* = 8.2 Hz, 1H), 6.95 (m, 3H), 4.57–4.09 (m, 4H), 4.05–3.08 (m, 7H), 1.28 (d, *J* = 7.2 Hz, 3H), 1.09 (m, 3H). ^13^C NMR (151 MHz, CDCl_3_) δ 167.39, 164.59, 149.17, 144.98, 143.92, 139.42, 135.45, 133.77, 130.63, 128.09, 124.29, 121.66, 117.78, 115.87, 62.56, 60.02, 52.87, 52.41, 47.21, 42.19, 14.19, 12.81. HRMS: Calcd for [C_22_H_24_ClN_3_O_6_S + H]^+^ = 494.1147, found = 494.1143.

#### Ethyl 2-((4-((*R*)-4-(3-Bromophenyl)-3-methylpiperazine-1-carbonyl)-2-nitrophenyl)sulfinyl)acetate ((*S*)**-25**)

The title compound was synthesized using (*S*)-1-(3-bromophenyl)-2-methylpiperazine (34 mg, 0.13 mmol, 1 equiv), 4-((2-ethoxy-2-oxoethyl)sulfinyl)-3-nitrobenzoic acid (40 mg, 0.13 mmol, 1 equiv), oxalyl chloride (19 mg, 0.15 mmol, 1.1 equiv), and DiPEA (52 mg, 0.40 mmol, 3 equiv) according to general procedure A in a yield of 40 mg (0.07 mmol, 56%). ^1^H NMR (400 MHz, CDCl_3_) δ 8.47–8.34 (m, 2H), 8.14–7.89 (m, 1H), 7.14 (m, 1H), 7.02 (m, 2H), 6.83 (d, *J* = 8.3 Hz, 1H), 4.42 (m, 1H), 4.22 (m, 2H), 4.14 (d, *J* = 13.7 Hz, 1H), 4.01 (m, 1H), 3.78 (d, *J* = 13.6 Hz, 1H), 3.72–3.02 (m, 5H), 1.28 (d, *J* = 7.1 Hz, 3H), 1.07 (m, 3H). ^13^C NMR (101 MHz, CDCl_3_) δ 167.36, 164.58, 150.78, 144.94, 144.06, 139.70, 133.73, 130.69, 128.00, 124.23, 123.47, 123.40, 120.06, 115.66, 62.49, 60.08, 52.62, 51.84, 47.38, 42.40, 14.23, 12.66. HRMS: Calcd for [C_22_H_24_BrN_3_O_6_S + H]^+^ = 538.0642, found = 538.0638.

#### Ethyl 2-((4-((*R*)-4-(3-Bromophenyl)-3-methylpiperazine-1-carbonyl)-2-nitrophenyl)sulfinyl)acetate ((*R*)**-25**)

The title compound was synthesized using (*R*)-1-(3-bromophenyl)-2-methylpiperazine (34 mg, 0.13 mmol, 1 equiv), 4-((2-ethoxy-2-oxoethyl)sulfinyl)-3-nitrobenzoic acid (40 mg, 0.13 mmol, 1 equiv), oxalyl chloride (19 mg, 0.15 mmol, 1.1 equiv), and DiPEA (52 mg, 0.40 mmol, 3 equiv) according to general procedure A in a yield of 50 mg (0.09 mmol, 70%). ^1^H NMR (600 MHz, CDCl_3_) δ 8.51–8.35 (m, 2H), 8.03 (dd, *J* = 8.0, 1.6 Hz, 1H), 7.23–7.09 (m, 3H), 6.96 (m, 1H), 4.50–4.04 (m, 4H), 3.99–3.13 (m, 7H), 1.27 (t, *J* = 7.1 Hz, 3H), 1.16–0.92 (m, 3H). ^13^C NMR (151 MHz, CDCl_3_) δ 167.42, 164.59, 149.38, 144.99, 143.89, 139.36, 133.78, 130.94, 128.10, 124.86, 124.31, 121.41, 117.19, 62.58, 60.01, 53.10, 52.37, 47.17, 42.14, 14.19, 12.89. HRMS: Calcd for [C_22_H_24_BrN_3_O_6_S + H]^+^ = 538.0642, found = 538.0639.

#### Ethyl 2-((4-((*R*)-3-Methyl-4-(3-(trifluoromethyl)phenyl)piperazine-1-carbonyl)-2-nitrophenyl)sulfinyl)acetate ((*R*)**-26**)

The title compound was synthesized using (*R*)-1-(3-bromophenyl)-2-methylpiperazine (32 mg, 0.13 mmol, 1 equiv), 4-((2-ethoxy-2-oxoethyl)sulfinyl)-3-nitrobenzoic acid (40 mg, 0.13 mmol, 1 equiv), oxalyl chloride (19 mg, 0.15 mmol, 1.1 equiv), and DiPEA (52 mg, 0.40 mmol, 3 equiv) according to general procedure A in a yield of 60 mg (0.11 mmol, 86%). ^1^H NMR (600 MHz, CDCl_3_) δ 8.51–8.33 (m, 2H), 8.05 (dd, *J* = 8.0, 1.6 Hz, 1H), 7.44 (m, 1H), 7.20 (m, 3H), 4.61–4.13 (m, 4H), 4.11–3.16 (m, 7H), 1.28 (t, *J* = 7.2 Hz, 3H), 1.19–1.01 (m, 3H). ^13^C NMR (151 MHz, CDCl_3_) δ 167.47, 164.60, 148.82, 145.00, 143.79, 139.39, 133.78, 131.97 (q, *J* = 32.0 Hz), 130.18, 128.10, 124.09 (q, *J* = 271.8 Hz), 124.31, 120.76, 118.53, 114.60, 62.59, 59.99, 52.98, 52.47, 47.33, 42.28, 14.17, 12.81. HRMS: Calcd for [C_23_H_24_F_3_N_3_O_6_S + H]^+^ = 528.1411, found = 528.1409.

#### Ethyl-2-((4-((*S*)-3-Methyl-4-(3-(trifluoromethyl)phenyl)piperazine-1-carbonyl)-2-nitrophenyl)sulfinyl)acetate ((*S*)**-26**)

The title compound was synthesized using (*S*)-1-(3-bromophenyl)-2-methylpiperazine (32 mg, 0.13 mmol, 1 equiv), 4-((2-ethoxy-2-oxoethyl)sulfinyl)-3-nitrobenzoic acid (40 mg, 0.13 mmol, 1 equiv), oxalyl chloride (19 mg, 0.15 mmol, 1.1 equiv), and DiPEA (52 mg, 0.40 mmol, 3 equiv) according to general procedure A in a yield of 57 mg (0.11 mmol, 82%). ^1^H NMR (400 MHz, CDCl_3_) δ 8.46–8.36 (m, 2H), 8.03 (dt, *J* = 8.0, 1.9 Hz, 1H), 7.40 (t, *J* = 7.9 Hz, 1H), 7.20–6.98 (m, 3H), 4.62–4.29 (m, 1H), 4.26–4.18 (m, 2H), 4.14 (d, *J* = 13.7 Hz, 1H), 4.11–3.83 (m, 1H), 3.79 (d, *J* = 13.7 Hz, 1H), 3.74–3.06 (m, 5H), 1.29 (t, *J* = 7.2 Hz, 3H), 1.20–0.93 (m, 3H). ^13^C NMR (101 MHz, CDCl_3_) δ 167.43, 164.61, 149.71, 144.96, 144.08, 139.68, 133.74, 131.78 (q, *J* = 31.8 Hz), 129.98, 128.02, 124.24, 124.22 (q, *J* = 273.71 Hz), 120.16, 117.04, 113.59, 62.49, 60.08, 52.61, 51.80, 47.38, 42.38, 14.21, 12.66. HRMS: Calcd for [C_23_H_24_F_3_N_3_O_6_S + H]^+^ = 528.1411, found = 528.1411.

#### Methyl-2-((4-((*R*)-4-(3-Chlorophenyl)-3-methylpiperazine-1-carbonyl)-2-nitrophenyl)sulfinyl)acetate (**27**)

The title compound was synthesized using 2-((4-((*R*)-4-(3-chlorophenyl)-3-methylpiperazine-1-carbonyl)-2-nitrophenyl)sulfinyl)acetic acid (40 mg, 0.09 mmol, 1 equiv), MeOH (55 mg, 1.72 mmol, 20 equiv), oxalyl chloride (12 mg, 0.94 mmol, 1.1 equiv), and DiPEA (33 mg, 0.26 mmol, 3 equiv) according to general procedure A in a yield of 28 mg (0.061 mmol, 68%). ^1^H NMR (600 MHz, CDCl_3_) δ 8.50–8.30 (m, 2H), 8.04 (dd, *J* = 8.0, 1.6 Hz, 1H), 7.24 (t, *J* = 8.2 Hz, 1H), 7.05–6.79 (m, 3H), 4.57–3.61 (m, 8H), 3.60–3.12 (m, 4H), 1.09 (d, *J* = 78.9 Hz, 3H). ^13^C NMR (151 MHz, CDCl_3_) δ 167.42, 165.03, 149.47, 145.03, 143.88, 139.53, 135.46, 133.81, 130.62, 128.05, 124.33, 122.12, 118.30, 116.40, 59.90, 53.18, 52.71, 52.44, 46.22, 42.24, 12.80. HRMS: Calcd for [C_21_H_22_ClN_3_O_6_S + H]^+^ = 480.0991, found = 480.0989.

#### 2-((4-((*R*)-4-(3-Chlorophenyl)-3-methylpiperazine-1-carbonyl)-2-nitrophenyl)sulfinyl)-*N*-ethyl-*N*-methylacetamide (**28**)

The title compound was synthesized using 2-((4-((*R*)-4-(3-chlorophenyl)-3-methylpiperazine-1-carbonyl)-2-nitrophenyl)sulfinyl)acetic acid (40 mg, 0.09 mmol, 1 equiv), *N*-methylethanamine (102 mg, 1.72 mmol, 20 equiv), oxalyl chloride (12 mg, 0.94 mmol, 1.1 equiv), and DiPEA (33 mg, 0.26 mmol, 3 equiv) according to general procedure A in a yield of 18 mg (0.036 mmol, 41%). ^1^H NMR (600 MHz, CDCl_3_) δ 8.39 (m, 2H), 8.01 (d, *J* = 7.8 Hz, 1H), 7.22 (t, *J* = 8.0 Hz, 1H), 6.88 (m, 3H), 4.36–3.91 (m, 2H), 3.76 (m, 3H), 3.58–3.32 (m, 4H), 3.32–3.12 (m, 2H), 3.09–2.95 (m, 3H), 1.27–0.91 (m, 6H). ^13^C NMR (151 MHz, CDCl_3_) δ 167.22, 163.56, 150.06, 145.10, 144.32, 139.36, 135.37, 133.70, 130.52, 128.10, 124.16, 121.00, 117.45, 115.51, 59.51, 52.40, 47.45, 45.51, 43.43, 42.30, 33.36, 13.72, 12.88. HRMS: Calcd for [C_23_H_27_ClN_4_O_5_S + H]^+^ = 507.1463, found = 507.1463.

#### Isopropyl-2-((4-((*R*)-4-(3-Chlorophenyl)-3-methylpiperazine-1-carbonyl)-2-nitrophenyl)sulfinyl)acetate (**29**)

The title compound was synthesized using 2-((4-((*R*)-4-(3-chlorophenyl)-3-methylpiperazine-1-carbonyl)-2-nitrophenyl)sulfinyl)acetic acid (40 mg, 0.09 mmol, 1 equiv), propan-2-ol (103 mg, 1.72 mmol, 20 equiv), oxalyl chloride (12 mg, 0.94 mmol, 1.1 equiv), and DiPEA (33 mg, 0.26 mmol, 3 equiv) according to general procedure A in a yield of 16 mg (0.03 mmol, 37%). ^1^H NMR (600 MHz, CDCl_3_) δ 8.50–8.33 (m, 2H), 8.03 (dd, *J* = 8.0, 1.6 Hz, 1H), 7.22 (t, *J* = 8.1 Hz, 1H), 6.88 (m, 3H), 5.07 (m, 1H), 4.52–4.38 (m, 1H), 4.13 (d, *J* = 13.8 Hz, 1H), 3.90 (m, 1H), 3.76 (d, *J* = 13.8 Hz, 1H), 3.74–3.10 (m, 5H), 1.31–1.22 (m, 6H), 1.14–1.02 (m, 3H). ^13^C NMR (151 MHz, CDCl_3_) δ 167.13, 164.17, 150.12, 145.00, 144.12, 139.55, 135.39, 133.76, 130.54, 128.19, 124.28, 121.53, 117.52, 115.53, 70.72, 60.24, 52.36, 47.38, 45.08, 42.35, 21.83, 12.75. HRMS: Calcd for [C_23_H_26_ClN_3_O_6_S + H]^+^ = 508.1304, found = 508.1305.

#### 2,2,2-Trifluoroethyl-2-((4-((*R*)-4-(3-Chlorophenyl)-3-methylpiperazine-1-carbonyl)-2-nitrophenyl)sulfinyl)acetate (**30**)

The title compound was synthesized using 2-((4-((*R*)-4-(3-chlorophenyl)-3-methylpiperazine-1-carbonyl)-2-nitrophenyl)sulfinyl)acetic acid (40 mg, 0.09 mmol, 1 equiv), 2,2,2-trifluoroethan-1-ol (172 mg, 1.72 mmol, 20 equiv), oxalyl chloride (12 mg, 0.94 mmol, 1.1 equiv), and DiPEA (33 mg, 0.26 mmol, 3 equiv) according to general procedure A in a yield of 8 mg (0.015 mmol, 17%). ^1^H NMR (600 MHz, CDCl_3_) δ 8.50–8.33 (m, 2H), 8.10–7.95 (m, 1H), 7.22 (t, *J* = 8.0 Hz, 1H), 6.95–6.75 (m, 3H), 4.52 (qd, *J* = 8.2, 1.8 Hz, 2H), 4.35–3.05 (m, 9H), 1.07 (m, 3H). ^13^C NMR (151 MHz, CDCl_3_) δ 167.35, 163.16, 150.41, 145.00, 143.45, 140.02, 135.38, 133.95, 130.51, 128.18, 124.42, 122.56 (q, *J* = 277.4 Hz), 120.88, 117.47, 115.46, 61.54 (q, *J* = 37.3 Hz), 59.13, 52.62, 52.19, 47.49, 42.44, 12.72. HRMS: Calcd for [C_22_H_21_ClF_3_N_3_O_6_S + H]^+^ = 548.0864, found = 548.0864.

#### Propyl 2-((4-((*R*)-4-(3-Chlorophenyl)-3-methylpiperazine-1-carbonyl)-2-nitrophenyl)sulfinyl)acetate (**31**)

The title compound was synthesized using 2-((4-((*R*)-4-(3-chlorophenyl)-3-methylpiperazine-1-carbonyl)-2-nitrophenyl)sulfinyl)acetic acid (40 mg, 0.09 mmol, 1 equiv), propan-1-ol (103 mg, 1.72 mmol, 20 equiv), oxalyl chloride (12 mg, 0.94 mmol, 1.1 equiv), and DiPEA (33 mg, 0.26 mmol, 3 equiv) according to general procedure A in a yield of 26 mg (0.051 mmol, 60%). ^1^H NMR (850 MHz, CDCl_3_) δ 8.42 (d, *J* = 7.9 Hz, 2H), 8.04 (d, *J* = 8.1 Hz, 1H), 7.24 (s, 1H), 7.05–6.85 (m, 3H), 4.50–3.14 (m, 11H), 1.69 (p, *J* = 7.0 Hz, 2H), 1.20–0.98 (m, 3H), 0.96 (td, *J* = 7.4, 1.1 Hz, 3H). ^13^C NMR (214 MHz, CDCl_3_) δ 167.08, 164.70, 149.57, 145.00, 144.09, 139.47, 135.46, 133.78, 130.63, 128.10, 124.32, 121.58, 118.31, 116.32, 68.10, 60.15, 52.73, 52.45, 47.17, 42.22, 21.94, 12.77, 10.41. HRMS: Calcd for [C_23_H_26_ClN_3_O_6_S + H]^+^ = 508.13036, found = 508.13022.

#### Butyl 2-((4-((*R*)-4-(3-Chlorophenyl)-3-methylpiperazine-1-carbonyl)-2-nitrophenyl)sulfinyl)acetate (**32**)

The title compound was synthesized using 2-((4-((*R*)-4-(3-chlorophenyl)-3-methylpiperazine-1-carbonyl)-2-nitrophenyl)sulfinyl)acetic acid (40 mg, 0.09 mmol, 1 equiv), butan-1-ol (127 mg, 1.72 mmol, 20 equiv), oxalyl chloride (12 mg, 0.94 mmol, 1.1 equiv), and DiPEA (33 mg, 0.26 mmol, 3 equiv) according to general procedure A in a yield of 22 mg (0.042 mmol, 49%). ^1^H NMR (850 MHz, CDCl_3_) δ 8.45–8.36 (m, 2H), 8.03 (dd, *J* = 8.0, 1.6 Hz, 1H), 7.21 (t, *J* = 8.1 Hz, 1H), 6.94–6.86 (m, 2H), 6.84–6.78 (m, 1H), 4.41–3.11 (m, 11H), 1.67–1.61 (m, 2H), 1.39 (m, 2H), 1.17–0.98 (m, 3H), 0.94 (td, *J* = 7.4, 1.6 Hz, 3H). ^13^C NMR (214 MHz, CDCl_3_) δ 167.08, 164.74, 150.24, 144.97, 143.99, 139.60, 135.34, 133.77, 130.50, 128.05, 124.29, 120.80, 117.87, 115.94, 66.42, 60.17, 52.14, 47.37, 44.79, 42.36, 30.52, 19.11, 13.77, 12.64. HRMS: Calcd for [C_24_H_28_ClN_3_O_6_S + H]^+^ = 522.14601, found = 522.14572.

#### *sec*-Butyl 2-((4-((*R*)-4-(3-Chlorophenyl)-3-methylpiperazine-1-carbonyl)-2-nitrophenyl)sulfinyl)acetate (**33**)

The title compound was synthesized using 2-((4-((*R*)-4-(3-chlorophenyl)-3-methylpiperazine-1-carbonyl)-2-nitrophenyl)sulfinyl)acetic acid (40 mg, 0.09 mmol, 1 equiv), butan-2-ol (127 mg, 1.72 mmol, 20 equiv), oxalyl chloride (12 mg, 0.94 mmol, 1.1 equiv), and DiPEA (33 mg, 0.26 mmol, 3 equiv) according to general procedure A in a yield of 12 mg (0.023 mmol, 27%). ^1^H NMR (850 MHz, CDCl_3_) δ 8.48–8.35 (m, 2H), 8.08–8.01 (m, 1H), 7.28 (m, 1H), 7.11–6.91 (m, 3H), 4.93 (m, 1H), 4.47–3.20 (m, 9H), 1.65 (m, 1H), 1.62–1.53 (m, 1H), 1.25 (m, 3H), 1.18–1.04 (m, 3H), 0.95–0.90 (m, 3H). ^13^C NMR (214 MHz, CDCl_3_) δ 167.18, 164.32, 147.91, 145.00, 144.07, 139.13, 135.67, 133.83, 130.84, 128.24, 124.38, 123.57, 118.95, 117.16, 75.40, 60.20, 54.46, 52.24, 46.99, 41.97, 28.78, 19.44, 13.27, 9.70. HRMS: Calcd for [C_24_H_28_ClN_3_O_6_S + H]^+^ = 522.14601, found = 522.14612.

#### *tert*-Pentyl 2-((4-((*R*)-4-(3-Chlorophenyl)-3-methylpiperazine-1-carbonyl)-2-nitrophenyl)sulfinyl)acetate (**34**)

The title compound was synthesized using 2-((4-((*R*)-4-(3-chlorophenyl)-3-methylpiperazine-1-carbonyl)-2-nitrophenyl)sulfinyl)acetic acid (40 mg, 0.09 mmol, 1 equiv), 2-methylbutan-2-ol (151 mg, 1.72 mmol, 20 equiv), oxalyl chloride (12 mg, 0.94 mmol, 1.1 equiv), and DiPEA (33 mg, 0.26 mmol, 3 equiv) according to general procedure A. This yielded the product (12 mg, 0.022 mmol, 26%). ^1^H NMR (850 MHz, CDCl_3_) δ 8.49–8.38 (m, 2H), 8.04 (d, *J* = 8.0 Hz, 1H), 7.28 (s, 1H), 7.05 (s, 3H), 4.51–3.19 (m, 9H), 1.80 (tt, *J* = 14.1, 6.8 Hz, 2H), 1.47 (d, *J* = 13.0 Hz, 6H), 1.19–1.05 (m, 3H), 0.93 (t, *J* = 7.5 Hz, 3H). ^13^C NMR (214 MHz, CDCl_3_) δ 167.13, 163.73, 145.01, 144.43, 139.13, 135.68, 133.80, 130.84, 128.26, 124.36, 86.81, 61.28, 53.78, 52.28, 46.94, 41.87, 33.64, 25.56, 13.34, 8.34. HRMS: Calcd for [C_25_H_30_ClN_3_O_6_S + H]^+^ = 536.1617, found = 536.1617.

#### 3-Hydroxypropyl-2-((4-((*R*)-4-(3-Chlorophenyl)-3-methylpiperazine-1-carbonyl)-2-nitrophenyl)sulfinyl)acetate (**35**)

The title compound was synthesized using 2-((4-((*R*)-4-(3-chlorophenyl)-3-methylpiperazine-1-carbonyl)-2-nitrophenyl)sulfinyl)acetic acid (40 mg, 0.09 mmol, 1 equiv), propane-1,3-diol (131 mg, 1.72 mmol, 20 equiv), oxalyl chloride (12 mg, 0.94 mmol, 1.1 equiv), and DiPEA (33 mg, 0.26 mmol, 3 equiv) according to general procedure A in a yield of 12 mg (0.040 mmol, 47%). ^1^H NMR (850 MHz, CDCl_3_) δ 8.50–8.34 (m, 2H), 8.05 (s, 1H), 7.25 (s, 1H), 6.97 (m, 3H), 4.59–3.12 (m, 13H), 3.01 (s, 1H), 1.93–1.75 (m, 2H), 1.21–0.97 (m, 3H). ^13^C NMR (214 MHz, CDCl_3_) δ 167.21, 164.38, 149.95, 145.04, 143.65, 139.57, 135.51, 133.86, 130.67, 128.26, 124.29, 121.56, 118.46, 116.56, 64.61, 63.54, 59.10, 58.95, 52.47, 47.20, 42.19, 31.18, 13.06.

#### Ethyl-2-((4-(4-(3-Chlorophenyl)-3,5-dimethylpiperazine-1-carbonyl)-2-nitrophenyl)sulfinyl)acetate (**36**)

The title compound was synthesized using 4-((2-ethoxy-2-oxoethyl)sulfinyl)-3-nitrobenzoic acid (40 mg, 0.13 mmol, 1 equiv), 1-(3-chlorophenyl)-2,6-dimethylpiperazine (30 mg, 0.13 mmol, 1 equiv), oxalyl chloride (19 mg, 0.15 mmol, 1.1 equiv), and DiPEA (52 mg, 0.40 mmol, 3 equiv) according to general procedure A in a yield of 36 mg (0.07 mmol, 53%). ^1^H NMR (850 MHz, CDCl_3_) δ 8.49 (s, 1H), 8.42 (d, *J* = 8.0 Hz, 1H), 8.09 (d, *J* = 8.0 Hz, 1H), 7.55–7.39 (m, 4H), 4.76 (s, 1H), 4.25–4.12 (m, 3H), 3.92 (s, 1H), 3.80 (d, *J* = 13.9 Hz, 1H), 3.75–3.33 (m, 4H), 1.28 (t, *J* = 7.2 Hz, 3H), 1.06 (m, 6H). ^13^C NMR (214 MHz, CDCl_3_) δ 166.57, 164.68, 145.16, 144.39, 142.76, 138.73, 136.19, 133.88, 131.35, 129.33, 128.14, 124.78, 123.92, 123.03, 62.61, 60.63, 60.03, 51.89, 46.59, 16.21, 14.21. HRMS: Calculated for [C_23_H_26_ClN_3_O_6_S + H]^+^ = 508.1304, found = 508.1303.

#### Ethyl-2-((4-((*R*)-4-(3-Chlorophenyl)-3-methylpiperazine-1-carbonyl)-2-fluorophenyl)sulfinyl)acetate (**37**)

The title compound was synthesized using 4-((2-ethoxy-2-oxoethyl)sulfinyl)-3-fluorobenzoic acid (40 mg, 0.15 mmol, 1 equiv), (*R*)-1-(3-chlorophenyl)-2-methylpiperazine (31 mg, 0.15 mmol, 1 equiv), oxalyl chloride (20 mg, 0.16 mmol, 1.1 equiv), and DiPEA (57 mg, 0.44 mmol, 3 equiv) according to general procedure A in a yield of 36 mg (0.07 mmol, 53%). ^1^H NMR (850 MHz, CDCl_3_) δ 7.95 (t, *J* = 7.2 Hz, 1H), 7.51 (d, *J* = 8.4 Hz, 1H), 7.31 (t, *J* = 11.5 Hz, 2H), 7.24–6.98 (m, 3H), 4.35–3.17 (m, 11H), 1.25 (t, *J* = 7.2 Hz, 3H), 1.22–0.94 (m, 3H). ^13^C NMR (214 MHz, CDCl_3_) δ 168.20, 164.20, 157.65 (d, *J* = 246.3 Hz), 146.52, 140.01 (d, *J* = 29.3 Hz), 135.80, 132.01, 131.01, 127.15, 124.17, 124.01, 118.82, 117.08, 115.23 (d, *J* = 22.0 Hz), 62.61, 58.57, 55.09, 51.80, 46.42, 41.44, 14.10, 13.39. HRMS: Calculated for [C_22_H_24_ClFN_2_O_4_S + H]^+^ = 467.1202, found = 467.1202.

#### Ethyl-2-((4-(4-(3-Chlorophenyl)-2-methylpiperazine-1-carbonyl)-2-fluorophenyl)sulfinyl)acetate (**38**)

The title compound was synthesized using 4-((2-ethoxy-2-oxoethyl)sulfinyl)-3-fluorobenzoic acid (18 mg, 0.07 mmol, 1 equiv), 1-(3-chlorophenyl)-3-methylpiperazine (14 mg, 0.07 mmol, 1 equiv), oxalyl chloride (9 mg, 0.07 mmol, 1.1 equiv), and DiPEA (25 mg, 0.20 mmol, 3 equiv) according to general procedure A in a yield of 15 mg (0.03 mmol, 49%). ^1^H NMR (850 MHz, CDCl_3_) δ 8.09–7.88 (m, 1H), 7.56–7.38 (m, 1H), 7.22 (m, 2H), 7.02–6.75 (m, 3H), 4.79 (m, 1H), 4.36–4.18 (m, 2H), 3.97 (d, *J* = 13.8 Hz, 1H), 3.81 (d, *J* = 13.9 Hz, 1H), 3.51 (m, 3H), 3.13–2.67 (m, 3H), 1.45 (m, 3H), 1.27 (t, *J* = 7.0 Hz, 3H). ^13^C NMR (214 MHz, CDCl_3_) δ 167.75, 164.30, 157.69 (d, *J* = 250.3 Hz), 152.28, 141.31, 135.19, 131.92 (d, *J* = 16.9 Hz), 130.38, 127.07, 123.74, 120.68, 116.95, 114.98, 114.77, 62.54, 58.99, 54.51, 49.37, 42.94, 37.36, 16.45, 14.19.

#### (±)-Ethyl-2-((4-(4-(3-Chlorophenyl)-cis-2,3-dimethylpiperazine-1-carbonyl)-2-fluorophenyl)sulfinyl)acetate (**±39**)

The title compound was synthesized using 4-((2-ethoxy-2-oxoethyl)sulfinyl)-3-fluorobenzoic acid (30 mg, 0.11 mmol, 1 equiv), *cis*-1-(3-chlorophenyl)-2,3-dimethylpiperazine (25 mg, 0.11 mmol, 1 equiv), oxalyl chloride (15 mg, 0.12 mmol, 1.1 equiv), and DiPEA (42 mg, 0.33 mmol, 3 equiv) according to general procedure A in a yield of 12 mg (0.03 mmol, 23%). ^1^H NMR (400 MHz, CDCl_3_) δ 7.95 (t, *J* = 7.2 Hz, 1H), 7.45 (d, *J* = 7.8 Hz, 1H), 7.32–7.14 (m, 4H), 7.10 (d, *J* = 7.9 Hz, 1H), 4.50–2.90 (m, 10H), 1.40 (dd, *J* = 6.6, 1.5 Hz, 3H), 1.26 (t, *J* = 7.1 Hz, 3H), 0.88 (d, *J* = 6.3 Hz, 3H). HRMS: Calcd for [C_22_H_24_ClFN_2_O_4_S + H]^+^ = 481.1359, found = 481.1357.

#### (±)-Ethyl-2-((4-(4-(3-Chlorophenyl)-trans-2,3-dimethylpiperazine-1-carbonyl)-2-fluorophenyl)sulfinyl)acetate (**±40**)

The title compound was synthesized using 4-((2-ethoxy-2-oxoethyl)sulfinyl)-3-fluorobenzoic acid (30 mg, 0.11 mmol, 1 equiv), (±) *trans*-1-(3-chlorophenyl)-2,3-dimethylpiperazine (25 mg, 0.11 mmol, 1 equiv), oxalyl chloride (15 mg, 0.12 mmol, 1.1 equiv), and DiPEA (42 mg, 0.33 mmol, 3 equiv) according to general procedure A in a yield of 18 mg (0.04 mmol, 34%). ^1^H NMR (400 MHz, CDCl_3_) δ 8.00–7.90 (m, 1H), 7.44 (q, *J* = 7.5 Hz, 1H), 7.26–7.12 (m, 2H), 6.90–6.61 (m, 3H), 4.88–4.55 (m, 1H), 4.23 (q, *J* = 7.1 Hz, 2H), 3.97 (d, *J* = 13.8 Hz, 1H), 3.81 (d, *J* = 13.9 Hz, 1H), 3.71–3.45 (m, 2H), 3.38–3.05 (m, 3H), 1.56–1.41 (m, 3H), 1.27 (t, *J* = 7.1 Hz, 3H), 1.17–0.96 (m, 3H). HRMS: Calcd for [C_22_H_24_ClFN_2_O_4_S + H]^+^ = 481.1359, found = 481.1358.

#### Ethyl-2-((4-(4-(3-Chlorophenyl)-3,3-dimethylpiperazine-1-carbonyl)-2-fluorophenyl)sulfinyl)acetate (**41**)

The title compound was synthesized using 4-((2-ethoxy-2-oxoethyl)sulfinyl)-3-fluorobenzoic acid (30 mg, 0.11 mmol, 1 equiv), 1-(3-chlorophenyl)-2,2-dimethylpiperazine (25 mg, 0.11 mmol, 1 equiv), oxalyl chloride (15 mg, 0.12 mmol, 1.1 equiv) and DiPEA (42 mg, 0.33 mmol, 3 equiv) according to general procedure A in a yield of 26 mg (0.05 mmol, 49%). ^1^H NMR (400 MHz, CDCl_3_) δ 7.96 (t, *J* = 7.2 Hz, 1H), 7.50 (s, 1H), 7.36 (d, *J* = 6.5 Hz, 2H), 7.29 (m, 3H), 4.27–3.30 (m, 10H), 1.27 (m, 6H), 1.12 (s, 3H). HRMS: Calcd for [C_22_H_24_ClFN_2_O_4_S + H]^+^ = 481.1359, found = 481.1358.

#### Ethyl-2-((4-(4-(3-Chlorophenyl)-2,2-dimethylpiperazine-1-carbonyl)-2-fluorophenyl)sulfinyl)acetate (**42**)

The title compound was synthesized using 4-((2-ethoxy-2-oxoethyl)sulfinyl)-3-fluorobenzoic acid (30 mg, 0.11 mmol, 1 equiv), 1-(3-chlorophenyl)-3,3-dimethylpiperazine (25 mg, 0.11 mmol, 1 equiv), oxalyl chloride (15 mg, 0.12 mmol, 1.1 equiv) and DiPEA (42 mg, 0.33 mmol, 3 equiv) according to general procedure A in a yield of 28 mg (0.06 mmol, 53%). ^1^H NMR (400 MHz, CDCl_3_) δ 7.95 (t, *J* = 7.0 Hz, 1H), 7.47 (s, 1H), 7.32–7.05 (m, 5H), 4.22 (q, *J* = 7.1 Hz, 2H), 4.07–3.15 (m, 8H), 1.26 (t, *J* = 7.1 Hz, 3H), 1.21 (s, 3H), 1.03 (s, 3H). HRMS: Calcd for [C_22_H_24_ClFN_2_O_4_S + H]^+^ = 481.1359, found = 481.1358.

#### (±)-Ethyl-2-((2-Chloro-4-(4-(3-chlorophenyl)-trans-2,3-dimethylpiperazine-1-carbonyl)phenyl)sulfinyl)acetate (**±43**)

The title compound was synthesized using (±) *trans*-1-(3-chlorophenyl)-2,3-dimethylpiperazine (23.2 mg, 0.10 μmol, 1 equiv), 3-chloro-4-((2-ethoxy-2-oxoethyl)sulfinyl)benzoic acid (30 mg, 0.10 mmol, 1 equiv), HATU (39.2 mg, 0.10 mmol, 1.00 equiv), and DiPEA (31 mg, 0.30 mmol, 3 equiv) according to general procedure B in a yield of 38.3 mg (77.3 μmol, 75%). ^1^H NMR (500 MHz, CDCl_3_) δ 8.01 (d, *J* = 8.0 Hz, 1H), 7.54 (dd, *J* = 8.0, 1.6 Hz, 1H), 7.51–7.42 (m, 1H), 7.16 (t, *J* = 8.0 Hz, 1H), 6.83–6.78 (m, 2H), 6.70 (d, *J* = 8.4 Hz, 1H), 4.80 (t, *J* = 6.7 Hz, 1H), 4.62 (s, 1H), 4.29–4.17 (m, 2H), 4.04 (dd, *J* = 14.1, 1.7 Hz, 1H), 3.87 (d, *J* = 7.0 Hz, 1H), 3.69 (dd, *J* = 14.0, 1.2 Hz, 1H), 3.67–3.60 (m, 1H), 3.57–3.49 (m, 1H), 3.37–3.06 (m, 3H), 1.52–1.44 (m, 3H), 1.27 (t, *J* = 7.1 Hz, 3H), 1.16–097 (m, 3H). ^13^C NMR (126 MHz, CDCl_3_) δ 168.8, 168.3, 164.5, 151.3, 142.4, 140.5, 135.3, 130.8, 130.4, 128.4, 127.7, 127.1, 126.3, 125.8, 119.5, 116.3, 114.3, 62.5, 58.4, 56.2, 55.6, 49.8, 42.4, 41.3, 40.5, 36.6, 17.8, 16.8, 14.2, 12.8, 12.6. HRMS: Calcd for [C_23_H_27_Cl_2_N_2_O_4_S + H]^+^ = 497.1063, found = 497.1065.

#### (±)-Ethyl-2-((2-Bromo-4-(4-(3-chlorophenyl)-trans-2,3-dimethylpiperazine-1-carbonyl)phenyl)sulfinyl)acetate (**±44**)

The title compound was synthesized using 3-bromo-4-((2-ethoxy-2-oxoethyl)sulfinyl)benzoic acid (50.0 mg, 0.150 mmol, 1equiv), (±)-1-(3-chlorophenyl)-trans-2,3-dimethylpiperazine (33.6 mg, 0.150 mmol, 1 equiv), HATU (85.0 mg, 0.23 mmol, 1.5 equiv), and DiPEA (58.0 mg, 0.450 mmol) according to general procedure B in a yield of 68.4 mg (0.126 mmol, 85%). ^1^H NMR (400 MHz, CDCl_3_) δ 8.02 (d, *J* = 8.0 Hz, 1H), 7.67 (d, *J* = 1.2 Hz, 1H), 7.62 (dd, *J* = 8.0, 1.4 Hz, 1H), 7.19 (t, *J* = 8.2 Hz, 1H), 6.83 (dd, *J* = 8.1, 1.2 Hz, 2H), 6.73 (d, *J* = 9.0 Hz, 1H), 4.93–4.53 (m, 1H), 4.26 (qd, *J* = 7.1, 1.4 Hz, 2H), 4.10 (dd, *J* = 14.1, 0.8 Hz, 1H), 3.97–3.02 (m, 6H), 1.51 (s, 3H), 1.30 (t, *J* = 7.1 Hz, 3H), 1.18–0.99 (m, 3H). ^13^C NMR (101 MHz, CDCl_3_) δ 164.43, 158.30, 151.18, 143.91, 140.39, 135.19, 131.26, 130.29, 127.30, 126.73, 119.48, 119.26, 116.19, 114.20, 62.50, 58.46, 56.08, 49.83, 40.37, 36.62, 17.73, 14.10, 12.47.

#### (±)-2-((2-Chloro-4-(4-(3-chlorophenyl)-trans-2,3-dimethylpiperazine-1-carbonyl)phenyl)sulfinyl)acetic Acid (**±45**)

To a solution of (±) ethyl 2-((2-chloro-4-(4-(3-chlorophenyl)-trans-2,3-dimethylpiperazine-1-carbonyl)phenyl)sulfinyl)acetate (180 mg, 0.36 mmol, 1 equiv) in MeOH (2 mL) were added TEA (2 mL) and water (2 mL). The reaction mixture was stirred at room temperature overnight. The reaction progress was monitored by TLC analysis. Upon full conversion of the starting materials, the mixture was acidified with 3 M HCl solution to pH 2, extracted with EtOAc, dried (MgSO_4_), filtered, and concentrated under reduced pressure. The residue was purified by silica gel column chromatography (MeOH/DCM, 1–5%) to afford the product (0.16 g, 0.35 mmol, 97%). ^1^H NMR (500 MHz, CDCl_3_) δ 8.03 (d, *J* = 8.1 Hz, 1H), 7.63–7.43 (m, 2H), 7.17 (t, *J* = 8.3 Hz, 1H), 6.85–6.77 (m, 2H), 6.73–6.69 (m, 1H), 4.85–4.59 (m, 2H), 4.08 (dd, *J* = 14.2, 3.0 Hz, 1H), 3.92–3.49 (m, 4H), 3.36–3.11 (m, 2H), 1.54–1.44 (m, 3H), 1.15–0.98 (m, 3H). ^13^C NMR (126 MHz, CDCl_3_) δ 176.17, 169.06, 151.35, 141.69, 140.54, 135.28, 130.87, 130.30, 128.51, 127.15, 126.01, 119.52, 116.25, 114.25, 57.76, 56.16, 49.88, 40.47, 36.69, 17.81, 12.84. HRMS: Calcd for [C_21_H_22_Cl_2_N_2_O_4_S + H]^+^ = 496.0750, found = 496.0746.

#### (±)-Ethyl-2-((2-Chloro-4-(4-(3-chlorophenyl)-trans-2,3-dimethylpiperazine-1-carbonyl)phenyl)thio)acetate (**±46**)

The title compound was synthesized using 3-chloro-4-((2-ethoxy-2-oxoethyl)thio)benzoic acid (0.15 mg, 0.54 mmol, 1 equiv) according to general procedure B. This yielded the product (0.25 g, 0.46 mmol, 85%). ^1^H NMR (400 MHz, CDCl_3_) δ 7.48–7.36 (m, 2H), 7.34–7.27 (m, 1H), 7.17 (t, *J* = 8.3 Hz, 1H), 6.80 (d, *J* = 6.1 Hz, 2H), 6.71 (d, *J* = 8.4 Hz, 1H), 4.84–4.53 (m, 1H), 4.21 (q, *J* = 7.1 Hz, 2H), 3.73 (s, 2H), 3.69–3.01 (m, 5H), 1.51–1.41 (m, 3H), 1.27 (t, *J* = 7.2 Hz, 3H), 1.06 (dd, *J* = 46.6, 6.5 Hz, 3H). ^13^C NMR (101 MHz, CDCl_3_) δ 169.52, 168.85, 151.39, 137.10, 135.16, 134.77, 133.25,130.25, 128.12, 128.87, 125.47, 119.18, 116.02, 114.05, 61.99, 56.06, 49.45, 40.43, 36.42, 34.69, 17.69, 14.13, 12.49. HRMS: Calcd for [C_23_H_26_Cl_2_N_2_O_3_S + H]+ = 483.1084, found = 483.1079.

#### (±)-Ethyl-2-((2-Chloro-4-(4-(3-chlorophenyl)-trans-2,3-dimethylpiperazine-1-carbonyl)phenyl)sulfonyl)acetate (**±47**)

The title compound was synthesized using *tert-*butyl 3-chloro-4-((2-ethoxy-2-oxoethyl)sulfonyl)benzoate (27 mg, 0.09 mmol, 1 equiv), (±)1-(3-chlorophenyl)-trans-2,3-dimethylpiperazine (20 mg, 0.09 mmol, 1 equiv), HATU (51 mg, 0.13 mmol, 1.5 equiv) and DiPEA (35 mg, 0.27 mmol, 3 equiv) according to general procedure B in a yield of 8 mg (0.02 mmol, 18%). ^1^H NMR (500 MHz, CDCl_3_) δ 8.21 (d, *J* = 8.1 Hz, 1H), 7.67–7.42 (m, 2H), 7.18 (t, *J* = 8.0 Hz, 1H), 6.86–6.78 (m, 2H), 6.72 (d, *J* = 8.5 Hz, 1H), 4.47 (s, 2H), 4.15 (q, *J* = 7.1 Hz, 2H), 3.95–3.00 (m, 6H), 1.49 (dd, *J* = 16.0, 6.7 Hz, 3H), 1.19 (td, *J* = 7.2, 3.0 Hz, 3H), 1.13 (d, *J* = 6.6 Hz, 2H), 1.02 (d, *J* = 6.5 Hz, 1H).

#### (±)-Ethyl-2-((2-Chloro-4-((4-(3-chlorophenyl)-trans-2,3-dimethylpiperazin-1-yl)methyl)phenyl)sulfinyl)acetate (**±48**)

The title compound was synthesized using (±) ethyl 2-((2-chloro-4-((4-(3-chlorophenyl)-*trans*-2,3-dimethylpiperazin-1-yl)methyl)phenyl)thio)acetate (19 mg, 0.04 mmol, 1 equiv) according to general procedure F in a yield of 6.3 mg (0.01 mmol, 32%). ^1^H NMR (500 MHz, CDCl_3_) δ 7.89 (d, *J* = 8.0 Hz, 1H), 7.55 (ddd, *J* = 8.0, 3.0, 1.5 Hz, 1H), 7.49 (dd, *J* = 3.9, 1.5 Hz, 1H), 7.14 (t, *J* = 8.0 Hz, 1H), 6.80 (t, *J* = 2.2 Hz, 1H), 6.76–6.68 m, 2H), 4.26–4.18 (m, 2H), 4.02 (dd, *J* = 13.7, 2.8 Hz, 1H), 3.75–3.66 (m, 2H), 3.61 (d, *J* = 14.3 Hz, 1H), 3.24–3.19 (m, 1H), 3.15 (dd, *J* = 4.0, 1.5 Hz, 1H), 2.85 (q, *J* = 6.4 Hz, 1H), 2.78 (td, *J* = 11.5, 4.2 Hz, 1H), 2.51 (dt, *J* = 11.5, 1.8 Hz, 1H), 1.27 (td, *J* = 7.2, 1.0 Hz, 3H), 1.22 (dd, *J* = 6.6, 1.4 Hz, 3H), 1.17 (d, *J* = 6.5 Hz, 3H). ^13^C NMR (126 MHz, CDCl_3_) δ 169.4, 152.1, 145.6, 139.3, 135.2, 130.2, 130.2, 129.8, 128.2, 126.4, 118.2, 115.6, 113.7, 62.3, 58.7, 58.2, 57.0, 56.8, 45.0, 42.0, 14.3, 13.0, 9.6. HRMS: Calcd for [C_23_H_28_Cl_2_N_2_O_3_S + H]^+^ = 483.1270, found = 483.1273.

#### (±)-Ethyl-3-(2-Chloro-4-(4-(3-chlorophenyl)-trans-2,3-dimethylpiperazine-1-carbonyl)phenyl)-3-oxopropanoate (**±49**)

To a solution of (±) 2-chloro-4-(4-(3-chlorophenyl)-trans-2,3-dimethylpiperazine-1-carbonyl)benzoic acid (12.0 mg, 29.0 μmol, 1.00 equiv) in dry THF (250 μL), carbonyldiimidazole (5.30 mg, 32.0 mmol, 1.1 equiv) was added, and the reaction mixture was stirred for 2 h. Then, a mixture of ethyl potassium malonate (5.00 mg, 29.0 μmol, 1.00 equiv) [which had been prepared from ethyl hydrogen malonate (1 g) and KOH (0.4 g) in abs. ethyl alcohol 4 mL], anhydr. MgCl_2_ (5.61 mg, 59.0 μmol, 2.00 equiv) and TEA (9.86 μL, 7.16 mg, 71.0 μmol, 2.40 equiv) were added. The reaction mixture was stirred further for 24 h. After concentration under reduced pressure, the obtained residue was resuspended in 2 m aq. HCl and extracted with DCM. The combined organic layers were washed with sat. aq. NaHCO_3_ and brine, dried (Na_2_SO_4_), and concentrated under reduced pressure. Purification by flash column chromatography (pentane/EtOAc, 3:1) resulted in the title compound as a colorless oil (0.82 mg, 1.72 μmol, 6%).^1^H NMR (500 MHz, CDCl_3_) δ 7.66 (dd, *J* = 10.0, 7.9 Hz, 1H), 7.48 (d, *J* = 4.5 Hz, 1H), 7.35 (dd, *J* = 13.4, 7.9 Hz, 1H), 7.20–7.14 (m, 1H), 6.84–6.79 (m, 2H), 6.71 (dd, *J* = 9.1, 2.1 Hz, 1H), 5.59–4.71 (m, 1H), 4.29 (q, *J* = 7.1 Hz, 1H), 4.21 (q, *J* = 7.1 Hz, 1H), 4.04 (s, 2H), 3.92–3.09 (m, 5H), 1.47 (s, 3H), 1.35 (t, *J* = 7.1 Hz, 1H), 1.26 (t, *J* = 7.1 Hz, 2H), 1.07 (s, 3H). ^13^C NMR (126 MHz, CDCl_3_) δ 172.7, 169.4, 166.8, 151.4, 139.0, 138.8, 135.4, 132.9, 132.2, 130.5, 130.4, 119.6, 119.5, 116.3, 114.3, 94.0, 61.8, 60.9, 56.3, 49.2, 14.4, 14.2. HRMS: Calcd for [C_24_H_28_Cl_2_N_2_O_4_S + H]^+^ = 477.1342, found = 477.1346.

#### (±)*iso*-Propyl-2-((2-Chloro-4-(4-(3-chlorophenyl)-trans-2,3-dimethylpiperazine-1-carbonyl)phenyl)sulfinyl)acetate (**±50**)

The title compound was synthesized using (±) 2-chloro-4-(4-(3-chlorophenyl)-trans-2,3-dimethylpiperazine-1-carbonyl)benzoic acid (30.0 mg, 64.0 μmol, 1.00 equiv), oxalyl chloride (2 M in DCM, 35.0 μL, 70.0 μmol, 1.10 equiv), 2-propanol (19.2 mg, 320 μmol, 5.00 equiv), and DiPEA (9.09 mg, 70.0 μmol, 1.10 equiv) according to general procedure A in a yield of 14.0 mg (27.5 μmol, 43%). ^1^H NMR (500 MHz, CDCl_3_) δ 8.02 (d, *J* = 7.9 Hz, 1H), 7.58–7.51 (m, 1H), 7.51–7.43 (m, 1H), 7.17 (t, *J* = 8.0 Hz, 1H), 6.84–6.79 (m, 2H), 6.71 (d, *J* = 8.4 Hz, 1H), 5.08–5.14 (m, 1H), 4.86–4.58 (m, 1H), 4.04 (d, *J* = 14.1 Hz, 1H), 3.92–3.07 (m, 6H), 1.53–1.44 (m, 3H), 1.32–1.24 (m, 6H), 1.16–0.99 (m, 3H). ^13^C NMR (126 MHz, CDCl_3_) δ 168.8, 164.2, 151.4, 142.7, 140.6, 135.4, 130.4, 126.2, 119.6, 116.3, 114.3, 70.6, 58.8, 56.3, 55.5, 49.7, 42.4, 41.4, 40.6, 36.6, 21.9, 17.9, 16.8, 12.9, 12.6. HRMS: Calcd for [C_24_H_28_Cl_2_N_2_O_4_S + H]^+^ = 511.1220, found = 511.1227.

#### (±)*sec*-Butyl-2-((2-Chloro-4-(4-(3-chlorophenyl)-trans-2,3-dimethylpiperazine-1-carbonyl)phenyl)sulfinyl)acetate (**±51**)

The title compound was synthesized using (±) 2-chloro-4-(4-(3-chlorophenyl)-trans-2,3-dimethylpiperazine-1-carbonyl)benzoic acid (50.0 mg, 107 μmol, 1.00 equiv), oxalyl chloride (2 M in DCM, 80.0 μL, 160 μmol, 1.50 equiv), butan-2-ol (49.0 μL, 39.5 mg, 533 μmol, 5.00 equiv), and DiPEA (28.0 μL, 20.7 mg, 160 μmol, 1.50 equiv) according to general procedure A in a yield of 14.1 mg (26.8 μmol, 25%). ^1^H NMR (400 MHz, CDCl_3_) δ 8.02 (dd, *J* = 7.9, 2.0 Hz, 1H), 7.59–7.51 (m, 1H), 7.50–7.42 (m, 1H), 7.17 (t, *J* = 8.0 Hz, 1H), 6.85–6.77 (m, 2H), 6.70 (dd, *J* = 7.9, 3.2 Hz, 1H), 5.00–4.90 (m, 1H), 4.85–4.57 (m, 1H), 4.04 (d, *J* = 14.0 Hz, 1H), 3.92–3.07 (m, 5H), 1.70–1.53 (m, 3H), 1.53–1.43 (m, 3H), 1.30–1.21 (m, 3H), 1.16–0.99 (m, 2H), 0.97–0.88 (m, 3H). ^13^C NMR (126 MHz, CDCl_3_) δ 164.4, 164.3, 151.4, 142.8, 140.7, 135.3, 130.8, 130.4, 128.6, 127.9, 127.0, 126.4, 126.2, 125.9, 119.6, 116.3, 114.3, 75.2, 59.0, 58.8, 56.2, 55.5, 49.7, 42.4, 41.4, 40.5, 36.6, 28.8, 19.5, 17.9, 16.8, 12.9, 12.6, 9.7. HRMS: Calcd for [C_25_H_30_Cl_2_N_2_O_4_S + H]^+^ = 525.1376, found = 525.1385.

#### (±)Cyclobutyl-2-((2-Chloro-4-(4-(3-chlorophenyl)-trans-2,3-dimethylpiperazine-1-carbonyl)phenyl)sulfinyl)acetate (**±52**)

The title compound was synthesized using (±) 2-chloro-4-(4-(3-chlorophenyl)-trans-2,3-dimethylpiperazine-1-carbonyl)benzoic acid (50.0 mg, 107 μmol, 1.00 equiv), oxalyl chloride (2 M in DCM, 80.0 μL, 160 μmol, 1.50 equiv), cyclobutanol (77 mg, 1.07 mmol, 10 equiv), and DiPEA (28.0 μL, 20.7 mg, 160 μmol, 1.50 equiv) according to general procedure A in a yield of 8 mg (0.015 mmol, 14%). ^1^H NMR (500 MHz, CDCl_3_) δ 8.03 (d, *J* = 8.0 Hz, 1H), 7.66–7.40 (m, 2H), 7.18 (t, *J* = 8.1 Hz, 1H), 6.89–6.76 (m, 2H), 6.72 (d, *J* = 8.4 Hz, 1H), 5.06 (tt, *J* = 7.9, 7.1 Hz, 1H), 4.88–3.00 (m, 8H), 2.45–2.27 (m, 2H), 2.19–2.00 (m, 2H), 1.89–1.57 (m, 2H), 1.49 (d, *J* = 10.0 Hz, 3H), 1.20–0.86 (m, 3H). ^13^C NMR (126 MHz, CDCl_3_) δ 169.03, 163.85, 151.33, 142.36, 140.47, 135.36, 130.95, 130.43, 128.36, 127.16, 125.85, 119.66, 116.37, 114.34, 70.27, 58.23, 56.27, 49.92, 40.54, 36.71, 30.37, 30.32, 18.42, 14.12, 12.61. HRMS: Calcd for [C_25_H_28_Cl_2_N_2_O_4_S + H]^+^ = 523.1220, found = 523.1218.

#### (±)Cyclopentyl-2-((2-Chloro-4-(4-(3-chlorophenyl)-trans-2,3-dimethylpiperazine-1-carbonyl)phenyl)sulfinyl)acetate (**±53**)

The title compound was synthesized using (±) 2-Chloro-4-(4-(3-chlorophenyl)-trans-2,3-dimethylpiperazine-1-carbonyl)benzoic acid (50.0 mg, 107 μmol, 1.00 equiv), oxalyl chloride (2 M in DCM, 80.0 μL, 160 μmol, 1.50 equiv), cyclopentanol (92 mg, 1.07 mmol, 10 equiv), and DiPEA (28.0 μL, 20.7 mg, 160 μmol, 1.50 equiv) according to general procedure A in a yield of 12 mg (0.022 mmol, 21%). ^1^H NMR (500 MHz, CDCl_3_) δ 8.03 (d, *J* = 8.0 Hz, 1H), 7.63–7.41 (m, 2H), 7.18 (t, *J* = 8.1 Hz, 1H), 6.87–6.76 (m, 2H), 6.72 (d, *J* = 8.7 Hz, 1H), 5.29–5.19 (m, 1H), 5.12–3.05 (m, 8H), 1.87 (q, *J* = 6.9 Hz, 2H), 1.79–1.40 (m, 9H), 1.22–0.88 (m, 3H). ^13^C NMR (126 MHz, CDCl_3_) δ 169.01, 164.34, 151.32, 140.35, 135.36, 130.95, 130.43, 128.58, 127.17, 126.39, 119.68, 116.38, 114.35, 79.90, 58.42, 56.28, 49.97, 40.53, 36.75, 32.84, 32.72, 23.81, 23.78, 17.89, 12.60. HRMS: Calcd for [C_26_H_30_Cl_2_N_2_O_4_S + H]^+^ = 537.1376, found = 537.1376.

#### (±)Cyclohexyl-2-((2-Chloro-4-(4-(3-chlorophenyl)-trans-2,3-dimethylpiperazine-1-carbonyl)phenyl)sulfinyl)acetate (**±54**)

The title compound was synthesized using (±) 2-chloro-4-(4-(3-chlorophenyl)-trans-2,3-dimethylpiperazine-1-carbonyl)benzoic acid (50.0 mg, 107 μmol, 1.00 equiv), oxalyl chloride (2 M in DCM, 80.0 μL, 160 μmol, 1.50 equiv), cyclohexanol (107 mg, 1.07 mmol, 10 equiv), and DiPEA (28.0 μL, 20.7 mg, 160 μmol, 1.50 equiv) according to general procedure A in a yield of 3 mg (0.005 mmol, 5%). ^1^H NMR (500 MHz, CDCl_3_) δ 8.03 (d, *J* = 8.0 Hz, 1H), 7.62–7.43 (m, 2H), 7.18 (t, *J* = 8.1 Hz, 1H), 6.87–6.77 (m, 2H), 6.71 (d, *J* = 8.3 Hz, 1H), 4.88 (tt, *J* = 8.9, 3.9 Hz, 1H), 4.84–2.94 (m, 8H), 1.70–1.20 (m, 13H), 1.20–0.96 (m, 3H). ^13^C NMR (126 MHz, CDCl_3_) δ 168.06, 163.57, 151.37, 142.76, 140.55, 134.94, 130.90, 130.40, 128.34, 127.08, 126.37, 119.62, 116.35, 114.32, 75.45, 58.72, 56.27, 49.78, 40.54, 36.65, 31.58, 25.34, 23.73, 17.89, 12.62. HRMS: Calcd for [C_27_H_32_Cl_2_N_2_O_4_S + H]^+^ = 551.1533, found = 551.1533.

#### (±)2,3-Dihydroxypropyl-2-((2-Chloro-4-(4-(3-chlorophenyl)-trans-2,3-dimethylpiperazine-1-carbonyl)phenyl)sulfinyl)acetate (**±55**)

The title compound was synthesized using (±) 2-chloro-4-(4-(3-chlorophenyl)-trans-2,3-dimethylpiperazine-1-carbonyl)benzoic acid (50.0 mg, 107 μmol, 1.00 equiv), oxalyl chloride (2 M in DCM, 80.0 μL, 160 μmol, 1.50 equiv), glycerol (98 mg, 1.07 mmol, 10 equiv), and DiPEA (28.0 μL, 20.7 mg, 160 μmol, 1.50 equiv) according to general procedure A. This yielded the product(3 mg, 6 μmol, 5%). ^1^H NMR (500 MHz, CDCl_3_) δ 7.98–7.87 (m, 1H), 7.60–7.42 (m, 2H), 7.18 (t, *J* = 8.1 Hz, 1H), 6.85–6.77 (m, 2H), 6.71 (d, *J* = 7.5 Hz, 1H), 4.87–3.05 (m, 13H), 1.95 (br, 2H), 1.50 (dd, *J* = 15.0, 6.7 Hz, 3H), 1.08 (m, 3H). HRMS: Calcd for [C_24_H_28_Cl_2_N_2_O_6_S + H]^+^ = 543.1118, found = 543.1117.

#### Ethyl-2-((2-Chloro-4-(−4-(5-chloropyridin-3-yl)-trans-2,3-dimethylpiperazine-1-carbonyl) phenyl)sulfinyl)acetate (**±56**)

The title compound was synthesized using 3-chloro-4-((2-ethoxy-2-oxoethyl)sulfinyl)benzoic acid (11.6 mg, 0.04 mmol, 1 equiv) and (±) 1-(5-chloropyridin-3-yl)-trans-2,3-dimethylpiperazine (9 mg, 0.04 mmol, 1 equiv) according to the general procedure B. This yielded the product (12.4 mg, 0.025 mmol, 62%). ^1^H NMR (400 MHz, CDCl_3_) 8.40 (s, 1H), 8.08 (s, 1H), 8.04 (d, *J* = 7.9 Hz, 1H), 7.62–7.54 (m, 1H), 7.47 (d, *J* = 13.7 Hz, 2H), 4.98–4.65 (m, 1H), 4.33–4.17 (m, 2H), 4.06 (dd, *J* = 14.0, 1.9 Hz, 1H), 4.00–3.19 (m, 6H), 1.52–1.17 (m, 9H). ^13^C NMR (101 MHz, CDCl_3_) δ 168.99, 164.36, 148.19, 142.53, 139.55, 135.34, 131.00, 130.26, 128.53, 128.24, 127.90, 127.09, 126.08, 62.57, 58.06, 54.94, 49.27, 40.23, 35.82, 17.67, 14.07, 13.76.

#### Ethyl-2-((2-Chloro-4-(−4-(2-chloropyridin-4-yl)-trans-2,3-dimethylpiperazine-1-carbonyl)phenyl)sulfinyl) acetate (**±57**)

The title compound was synthesized using 3-chloro-4-((2-ethoxy-2-oxoethyl)sulfinyl)benzoic acid (14.5 mg, 0.05 mmol) and (±) 1-(2-chloropyridin-4-yl)-trans-2,3-dimethylpiperazine (11 mg, 0.05 mmol, 1equiv) according to the general procedure B. This yielded the product (7.5 mg, 0.015 mmol, 30%). ^1^H NMR (400 MHz, CDCl_3_) δ 8.29 (d, *J* = 6.4 Hz, 1H), 8.04 (d, *J* = 7.9 Hz, 1H), 7.60–7.42 (m, 2H), 6.77 (d, *J* = 7.5 Hz, 2H), 4.96–4.62 (m, 1H), 4.24 (qd, *J* = 7.1, 1.5 Hz, 2H), 4.10–3.27 (m, 7H), 1.47–1.20 (m, 9H). ^13^C NMR (101 MHz, CDCl_3_) δ 168.88, 164.42, 158.23, 146.19, 143.54, 142.89, 139.39, 131.04, 128.60, 127.27, 126.28, 107.48, 106.82, 62.53, 58.17, 54.62, 49.33, 40.32, 35.84, 17.78, 15.38, 14.09.

#### Ethyl-2-((2-Chloro-4-(−4-(6-chloropyridin-2-yl)-trans-2,3-dimethylpiperazine-1-carbonyl) phenyl)sulfinyl)acetate (**±58**)

The title compound was synthesized using 3-chloro-4-((2-ethoxy-2-oxoethyl)sulfinyl)benzoic acid (24 mg, 0.082 mmol, 1 equiv) and (±) 1-(6-chloropyridin-2-yl)-trans-2,3-dimethylpiperazine (18.6 mg, 0.082 mmol) according to the general procedure B. This yielded the product (32.6 mg, 0.065 mmol, 79%). ^1^H NMR (400 MHz, CDCl_3_) δ 8.05 (dd, *J* = 8.0, 2.8 Hz, 1H), 7.61–7.41 (m, 3H), 6.66 (dd, *J* = 7.5, 2.9 Hz, 1H), 6.49 (dd, *J* = 12.1, 8.4 Hz, 1H), 4.91–4.56 (m, 1H), 4.38–4.05 (m, 5H), 3.77–3.06 (m, 4H), 1.45–1.14 (m, 9H). ^13^C NMR (101 MHz, CDCl_3_) δ 168.96, 164.51, 158.63, 149.76, 142.42, 140.45, 140.15, 130.89, 128.59, 127.19, 126.41, 112.80, 104.55, 62.59, 58.36, 51.75, 49.08, 38.85, 36.52, 17.81, 16.81, 14.21.

#### Ethyl-2-((2-Chloro-4-(−4-(4-chloropyridin-2-yl)-trans-2,3-dimethylpiperazine-1-carbonyl) phenyl)sulfinyl)acetate (**±59**)

The title compound was synthesized using 3-chloro-4-((2-ethoxy-2-oxoethyl)sulfinyl)benzoic acid (9.3 mg, 0.032 mmol, 1 equiv) and (±) 1-(4-chloropyridin-2-yl)-trans-2,3-dimethylpiperazine (7.2 mg, 0.032 mmol, 1 equiv) according to the general procedure B. This yielded the product (15.6 mg, 0.031 mmol, 98%). ^1^H NMR (400 MHz, CDCl_3_) δ 8.21 (t, *J* = 3.8 Hz, 1H), 8.06 (d, *J* = 6.1 Hz, 1H), 7.63–7.46 (m, 2H), 6.93–6.87 (m, 2H), 4.96–4.63 (m, 1H), 4.41–3.96 (m, 5H), 3.83–3.28 (m, 4H), 1.48–1.24 (m, 9H). ^13^C NMR (101 MHz, CDCl_3_) δ 168.89, 164.49, 155.08, 150.38, 143.34, 142.86, 139.46, 130.96, 128.56, 127.02, 126.32, 114.69, 109.82, 62.47, 58.29, 54.03, 49.17, 40.73, 35.82, 17.50, 15.63, 14.10.

#### (4-(3-Chlorophenyl)-trans-2,3-dimethylpiperazin-1-yl)(4-((2-ethoxyethyl)sulfinyl)-3-fluorophenyl)methanone (**±61**)

The title compound was synthesized using 4-((2-ethoxyethyl)sulfinyl)-3-fluorobenzoic acid (30 mg, 0.115 mmol, 1 equiv) according to general procedure B. This yielded the product (10 mg, 0.021 mmol, 19%). ^1^H NMR (400 MHz, CDCl_3_) δ 8.02 (dd, *J* = 8.1, 1.4 Hz, 1H), 7.85 (dd, *J* = 8.1, 6.6 Hz, 1H), 7.79 (dd, *J* = 9.8, 1.5 Hz, 1H), 7.15–7.18 (m, 1H), 6.92–6.63 (m, 3H), 4.88–4.55 (m, 1H), 4.23 (q, *J* = 7.1 Hz, 2H), 4.15–3.44 (m, 6H), 3.38–3.05 (m, 3H), 1.56–1.41 (m, 3H), 1.27 (t, *J* = 7.1 Hz, 3H), 1.17–0.96 (m, 3H).

#### Ethyl-2-((2-Chloro-4-(4-(3-chlorophenyl)-trans-2,3-dimethylpiperazine-1-carbonyl)phenyl)sulfinyl)propanoate (**±62**)

The title compound was synthesized using 3-chloro-4-((1-ethoxy-1-oxopropan-2-yl)sulfinyl)benzoic acid (0.10 g, 0.34 mmol, 1 equiv) according to general procedure B. This yielded the product (0.15 g, 0.29 mmol, 85%). ^1^H NMR (400 MHz, CDCl_3_) δ 7.95–7.85 (m, 1H), 7.60–7.39 (m, 2H), 7.17 (t, *J* = 8.3 Hz, 1H), 6.86–6.78 (m, 2H), 6.78–6.66 (m, 1H), 4.91–4.53 (m, 1H), 4.32 (q, *J* = 7.2 Hz, 1H), 4.10–3.05 (m, 7H), 1.56–1.43 (m, 3H), 1.40–1.31 (m, 3H), 1.31–1.24 (m, 3H), 1.17–0.97 (m, 3H). ^13^C NMR (126 MHz, CDCl_3_) δ 168.56, 166.53, 151.31, 140.49, 135.26, 131.61, 131.03, 130.34, 128.61, 128.10, 125.35, 119.45, 116.21, 114.20, 62.50, 60.52, 56.14, 40.47, 38.68, 36.50, 17.81, 14.21, 12.57, 6.60. HRMS: Calcd for [C_24_H_28_Cl_2_N_2_O_4_S + Na]^+^ = 535.1008, found = 535.1009.

#### *sec*-Butyl-2-((2-Chloro-4-(4-(3-chlorophenyl)-trans-2,3-dimethylpiperazine-1-carbonyl)phenyl)sulfinyl)propanoate (**±63**)

The title compound was synthesized using (±) 2-((2-chloro-4-(trans-4-(3-chlorophenyl)-2,3-dimethylpiperazine-1-carbonyl)phenyl)sulfinyl)propanoic acid (60 mg, 0.12 mmol, 1 equiv), butan-2-ol (1 mL, 10.87 mmol, 88 equiv), 2 M oxalyl chloride solution (0.07 mL, 0.14 mmol. 1.1 equiv), and DIPEA (0.1 mL, 0.57 mmol, 4.6 equiv) according to the H. This yielded the product (8.1 mg, 0.02 mmol, 12%). ^1^H NMR (500 MHz, CDCl_3_) δ 7.93 (d, 1H), 7.57–7.46 (m, 2H), 7.19 (t, *J* = 8.0 Hz, 1H), 6.86–6.81 (m, 2H), 6.74 (d, *J* = 8.4 Hz, 1H), 5.06–4.99 (m, 1H), 3.90 (qd, *J* = 7.2, 5.0 Hz, 1H), 3.84–3.11 (m, 6H), 1.77–1.60 (m, 2H), 1.51 (d, *J* = 6.7 Hz, 3H), 1.31 (dd, *J* = 6.3, 0.9 Hz, 3H), 1.27 (ddd, *J* = 7.3, 6.0, 1.5 Hz, 3H), 1.14–1.04 (m, 3H), 0.97 (dt, *J* = 11.0, 7.4 Hz, 3H). ^13^C NMR (126 MHz, CDCl_3_) δ 166.53, 165.29, 151.34, 141.97, 140.49, 135.38, 131.84, 130.42, 128.30, 127.91, 125.92, 119.66, 116.37, 114.35, 74.55, 74.38, 62.00, 61.35, 56.31, 28.74, 19.34, 12.46, 12.24, 9.61. HRMS: Calcd for [C_26_H_32_Cl_2_N_2_O_4_S + H]^+^ = 541.1503, found = 541.1501.

#### 1-Methoxypropan-2-yl-2-((2-Chloro-4-(4-(3-chlorophenyl)-trans-2,3-dimethylpiperazine-1-carbonyl)phenyl)sulfinyl)acetate (**±64**)

The title compound was synthesized using 1-methoxypropan-2-ol (1 mL, 10.21 mmol, 192 equiv) according to procedure H. This yielded the product (5.3 mg, 9.8 μmol, 18%). ^1^H NMR (500 MHz, CDCl_3_) δ 8.03 (d, *J* = 7.9 Hz, 1H), 7.64–7.39 (m, 2H), 7.18 (t, *J* = 8.1 Hz, 1H), 6.87–6.77 (m, 2H), 6.72 (d, *J* = 8.5 Hz, 1H), 5.24–5.11 (m, 1H), 5.04–4.54 (m, 1H), 4.08 (d, *J* = 10.6 Hz, 1H), 3.87 (s, 1H), 3.70 (dd, *J* = 14.0, 3.7, 0.8 Hz, 1H), 3.52–3.40 (m, 3H), 3.38 (s, 3H), 3.19 (s, 3H), 1.49 (d, *J* = 6.7 Hz, 3H), 1.28 (dd, *J* = 19.0, 6.5 Hz, 3H), 1.18–0.96 (m, 3H). HRMS: Calcd for [C_25_H_30_Cl_2_N_2_O_5_S + H]^+^ = 541.1325, found = 541.1323.

#### Benzo[*d*][1,3]dioxol-5-ylmethyl-2-((2-Chloro-4-(4-(3-chlorophenyl)-trans-2,3-dimethylpiperazine-1-carbonyl)phenyl)sulfinyl)acetate (**±65**)

The title compound was synthesized using benzo[d][1,3]dioxol-5-ylmethanol (20 mg, 0.13 mmol, 3 equiv) according to general procedure A. This yielded the product (8 mg, 0.01 mmol, 31%). ^1^H NMR (500 MHz, CDCl_3_) δ 7.98 (d, *J* = 8.0 Hz, 1H), 7.57–7.39 (m, 2H), 7.18 (t, *J* = 8.0 Hz, 1H), 6.88–6.76 (m, 5H), 6.71 (d, *J* = 8.4 Hz, 1H), 5.98 (s, 2H), 5.18–5.04 (m, 2H), 4.16–3.08 (m, 8H), 1.55–1.43 (m, 3H), 1.30–1.22 (m, 3H). HRMS: Calcd for [C_29_H_28_Cl_2_N_2_O_6_S + H]^+^ = 603.1118, found = 603.1116.

#### 2-((2-Chloro-4-(trans-4-(3-chlorophenyl)-2,3-dimethylpiperazine-1-carbonyl)phenyl)sulfinyl)-*N*-ethylacetamide (**±66**)

The title compound was synthesized using (±) 2-((2-chloro-4-(trans-4-(3-chlorophenyl)-2,3-dimethylpiperazine-1-carbonyl)phenyl)sulfinyl)propanoic acid (0.16 g, 0.34 mmol, 1 equiv) according to general procedure B. This yielded the product (1.5 mg, 3.0 μmol, 2%). ^1^H NMR (500 MHz, CDCl_3_) δ 8.20 (d, *J* = 8.0 Hz, 1H), 7.69–7.46 (m, 2H), 7.19 (t, *J* = 8.0 Hz, 1H), 6.82–6.74 (m, 2H), 6.74 (d, *J* = 8.5 Hz, 1H), 3.95–3.00 (m, 10H), 1.19–1.15 (m, 3H), 1.13 (d, *J* = 6.5 Hz, 3H), 1.02 (d, *J* = 6.5 Hz, 3H). HRMS: Calcd for [C_23_H_27_Cl_2_N_3_O_3_S + H]^+^ = 496.1223, found = 496.1220.

#### (3-Chloro-4-(((3-methyl-1,2,4-oxadiazol-5-yl)methyl)sulfinyl)phenyl)(−4-(3-chlorophenyl)-trans-2,3-dimethylpiperazin-1-yl)methanone (**±67**)

The title compound was synthesized using 3-chloro-4-(((3-methyl-1,2,4-oxadiazol-5-yl)methyl)sulfinyl)benzoic acid (45.0 mg, 0.150 mmol, 1 equiv) according to procedure B. This yielded the product (61.8 mg, 0.122 mmol, 81%). ^1^H NMR (400 MHz, CDCl_3_) δ 7.78 (d, *J* = 6.8 Hz, 1H), 7.48 (d, *J* = 12.7 Hz, 2H), 7.17 (t, *J* = 8.1 Hz, 1H), 6.81 (d, *J* = 7.0 Hz, 2H), 6.71 (d, *J* = 9.2 Hz, 1H), 4.85–4.38 (m, 3H), 3.92–3.02 (m, 5H), 2.37 (d, *J* = 3.4 Hz, 3H), 1.48 (d, *J* = 4.7 Hz, 3H), 1.06 (d, *J* = 43.9 Hz, 3H). ^13^C NMR (101 MHz, CDCl_3_) δ 169.68, 168.76, 167.82, 151.28, 141.28, 140.85, 135.29, 131.02, 130.38, 128.31, 126.81, 125.87, 119.60, 116.31, 114.28, 56.19, 55.67, 49.72, 40.48, 36.66, 17.80, 12.47, 11.55. LC-MS, *m*/*z*: Calcd for C_23_H_24_Cl_2_N_4_O_3_S, 506.09; found 507.29 ([M + H^+^]).

#### 3-((2-Chloro-4-(4-(3-chlorophenyl)-trans-2,3-dimethylpiperazine-1-carbonyl)phenyl)thio)-1,1,1-trifluoropropan-2-one (**±68**)

To a solution of trifluoroacetic anhydride (34 μL, 0.24 mmol, 2.2 equiv) in toluene (0.5 mL) was added (±) 2-((2-chloro-4-(4-(3-chlorophenyl)-trans-2,3-dimethylpiperazine-1-carbonyl)phenyl)thio)acetic acid (50 mg, 0.11 mmol, 1 equiv) and the mixture was cooled to 0 °C. Then, pyridine (22 μL, 0.28 mmol, 2.5 equiv) in toluene (0.5 mL) was slowly added and the mixture was stirred at 65 °C overnight. Afterward, it was cooled to 0 °C, and 1 mL water was added dropwise. Then, the temperature was brought up to 45 °C and maintained for 2h. After cooling, the aqueous phase of the mixture was separated and washed with ethyl acetate. The combined organic layers were washed with water and brine, dried (MgSO_4_), filtered, and concentrated. The crude product was purified by prep HPLC to provide a white solid (9.4 mg, 0.11 mmol, 17%). ^1^H NMR (400 MHz, CDCl_3_) δ 7.63–7.27 (m, 3H), 7.17 (t, *J* = 8.4 Hz, 1H), 6.81 (d, *J* = 7.2 Hz, 2H), 6.71 (d, *J* = 8.5 Hz, 1H), 4.69 (dd, *J* = 76.4, 10.1 Hz, 1H), 4.06 (s, 1H), 3.98–3.02 (m, 5H), 1.47 (dd, *J* = 9.7, 6.7 Hz, 3H), 1.06 (dd, *J* = 45.1, 6.7 Hz, 3H). HRMS: Calcd for [C_22_H_21_Cl_2_F_3_N_2_O_2_S + H_2_O + H]^+^ = 523.0831, found = 523.0828.

#### 3-((2-Chloro-4-(4-(3-chlorophenyl)-trans-2,3-dimethylpiperazine-1-carbonyl)phenyl)sulfinyl)-1,1,1-trifluoropropan-2-one (**±69**)

The title compound was synthesized using (±) (3-chloro-4-(methylsulfinyl)phenyl)(trans-4-(3-chlorophenyl)-2,3-dimethylpiperazin-1-yl)methanone (50 mg, 0.12 mmol, 1 equiv) and ethyl 2,2,2-trifluoroacetate (167 mg, 1.18 mmol, 10 equiv) according to general procedure C. This yielded the product (48 mg, 0.09 mmol, 79%). ^1^H NMR (600 MHz, CDCl_3_) δ 8.07 (d, *J* = 8.0 Hz, 1H), 7.66–7.47 (m, 2H), 7.19 (t, *J* = 8.1 Hz, 1H), 6.86–6.80 (m, 2H), 6.72 (dd, *J* = 8.4, 2.4 Hz, 1H), 3.77–2.99 (m, 7H), 1.52 (d, *J* = 6.8 Hz, 3H), 1.07 (m, 3H). ^13^C NMR (126 MHz, CDCl_3_) δ 168.76, 168.28, 151.30, 141.82, 140.78, 135.27, 130.60, 130.38, 128.68, 126.78, 121.76 (q, *J* = 287.3 Hz), 119.53, 116.26, 114.26, 93.88 (q, *J* = 33.8 Hz), 56.18, 53.97, 49.80, 40.45, 36.64, 17.81, 12.25. HRMS: Calcd for [C_22_H_21_Cl_2_F_3_N_2_O_3_S + H_2_O + H]^+^ = 539.0780, found = 539.0779.

#### (3-Chloro-4-((3,3,3-trifluoro-2-hydroxypropyl)sulfinyl)phenyl)(trans-4-(3-chlorophenyl)-2,3-dimethylpiperazin-1-yl)methanone (**±70**)

To a cooled (0 °C) solution of (±) 3-((2-chloro-4-(trans-4-(3-chlorophenyl)-2,3-dimethylpiperazine-1-carbonyl)phenyl)sulfinyl)-1,1,1-trifluoropropan-2-one 1,1,1-trifluoropropan-2-one (25 mg, 0.05 mmol, 1 equiv) in MeOH (1.7 mL) was added NaBH_4_ (1.5 mg, 0.04 mmol, 0.8 equiv). The resulting solution was stirred for 1 h. The reaction was quenched with sat. aq. NH_4_Cl, extracted with DCM (3×), dried (MgSO_4_), filtered, and concentrated. The crude product was purified with prep HPLC to afford the compound as a white solid (9.3 mg, 0.05 mmol, 37%). ^1^H NMR (500 MHz, CDCl_3_) δ 8.06–7.97 (m, 1H), 7.64–7.44 (m, 2H), 7.20 (t, *J* = 8.1 Hz, 1H), 6.86–6.81 (m, 2H), 6.71 (d, *J* = 8.4 Hz, 1H), 6.38 (s, 1H), 5.38–5.45 (m, 1H), 3.78–2.94 (m, 8H), 1.52 (d, *J* = 6.8 Hz, 3H), 1.07–0.96 (m, 3H). HRMS: Calcd for [C_22_H_23_Cl_2_F_3_N_2_O_3_S + H]+ = 523.0829, found = 523.0831.

#### 1-((2-Chloro-4-(4-(3-chlorophenyl)-trans-2,3-dimethylpiperazine-1-carbonyl)phenyl)sulfinyl)-3,3-difluorobutan-2-one (**±72**)

The title compound was synthesized using (±) (3-chloro-4-(methylsulfinyl)phenyl)(trans-4-(3-chlorophenyl)-2,3-dimethylpiperazin-1-yl)methanone (25 mg, 0.06 mmol, 1 equiv) and ethyl 2,2-difluoropropanoate (81 mg, 0.59 mmol, 10 equiv) according to general procedure C. This yielded the product (4.9 mg, 0.06 mmol, 16%). ^1^H NMR (500 MHz, CDCl_3_) δ 8.06 (dd, *J* = 18.3, 8.0 Hz, 1H), 7.63–7.49 (m, 2H), 7.20 (t, *J* = 8.0 Hz, 1H), 6.88–6.82 (m, 2H), 6.76 (d, *J* = 8.4 Hz, 1H), 4.44 (dd, *J* = 15.1, 4.4 Hz, 1H), 4.07 (dd, *J* = 15.1, 3.4 Hz, 1H), 3.85–3.10 (m, 6H), 1.78 (t, *J* = 19.3 Hz, 3H), 1.52 (d, *J* = 6.8 Hz, 3H), 1.10 (s, 3H). ^13^C NMR (101 MHz, CDCl_3_) δ 168.67, 168.19, 151.36, 142.33, 140.83, 135.32, 130.67, 130.40, 128.68, 126.98, 126.52, 119.56, 117.31 (t, *J* = 249 Hz), 116.30, 114.28, 59.45, 56.22, 49.72, 40.52, 36.58, 18.73 (t, *J* = 24 Hz), 17.87, 12.60. HRMS: Calcd for [C_23_H_24_Cl_2_F_2_N_2_O_3_S + H_2_O + H]^+^ = 535.1031, found = 535.1027.

#### 1-((2-Chloro-4-(4-(3-chlorophenyl)-trans-2,3-dimethylpiperazine-1-carbonyl)phenyl)sulfinyl)-3,3-difluoropentan-2-one (**±73**, **LEI-515**)

The title compound was synthesized using (±) (3-chloro-4-(methylsulfinyl)phenyl)(trans-4-(3-chlorophenyl)-2,3-dimethylpiperazin-1-yl)methanone (20 mg, 0.05 mmol, 1 equiv) and ethyl 2,2-difluorobutanoate (72 mg, 0.47 mmol, 10 equiv) according to general procedure C. This yielded the product (2.5 mg, 0.05 mmol, 10%). ^1^H NMR (400 MHz, CDCl_3_) δ 8.05–7.95 (m, 1H), 7.60–7.51 (m, 1H), 7.51–7.43 (m, 1H), 7.19–7.11 (m, 1H), 6.82–6.76 (m, 2H), 6.74–6.65 (m, 1H), 4.85–4.53 (m, 1H), 4.46–4.27 (m, 1H), 4.02 (ddd, *J* = 15.5, 5.0, 2.9 Hz, 1H), 3.92–3.79 (m, 1H), 3.74–3.57 (m, 1H), 3.56–3.46 (m, 1H), 3.40–3.03 (m, 2H), 2.16–1.97 (m, 2H), 1.48 (dd, *J* = 12.2, 6.7 Hz, 3H), 1.12 (d, *J* = 6.6 Hz, 1H), 1.07–0.90 (m, 5H). ^13^C NMR (101 MHz, CDCl_3_) δ 192.26 (t, *J* = 35.1 Hz), 168.56, 151.32, 142.28, 140.86, 135.21, 130.57, 130.33, 128.60, 127.03, 126.46, 119.42, 117.89 (dd, *J* = 249.9 Hz), 116.18, 114.28, 60.09, 56.11, 49.61, 40.43, 36.48, 25.45 (t, *J* = 23.1 Hz), 17.77, 12.52, 5.40 (t, *J* = 5.3 Hz). HRMS: Calcd for [C_24_H_26_Cl_2_F_2_N_2_O_3_S + H_2_O + H]^+^ = 549.1188, found = 549.1183.

#### 1-((2-Chloro-4-(4-(3-chlorophenyl)-trans-2,3-dimethylpiperazine-1-carbonyl)phenyl)sulfinyl)-3,3-difluoropentan-2-one (**(+)73**)

The title compound was synthesized using (+)-(3-chloro-4-(methylsulfinyl)phenyl)(trans-4-(3-chlorophenyl)-2,3-dimethylpiperazin-1-yl)methanone (30 mg, 71 μmol, 1 equiv) and ethyl 2,2-difluorobutanoate (107 mg, 0.47 mmol, 10 equiv) according to general procedure C. This yielded the product (15 mg, 28 mmol, 40%). [α]^20^_D_ = +16.2. ^1^H NMR (400 MHz, CDCl_3_) δ 8.07–7.99 (m, 1H), 7.63–7.52 (m, 1H), 7.54–7.43 (m, 1H), 7.22–7.12 (m, 1H), 6.86–6.78 (m, 2H), 6.76–6.66 (m, 1H), 4.89–4.56 (m, 1H), 4.45–4.33 (m, 1H), 4.10–3.97 (m, 1H), 3.92–3.78 (m, 1H), 3.74–3.59 (m, 1H), 3.57–3.49 (m, 1H), 3.47–3.05 (m, 2H), 2.07 (tt, *J* = 17.7, 7.7 Hz, 2H), 1.49 (dd, *J* = 13.0, 6.8 Hz, 3H), 1.13 (d, *J* = 6.5 Hz, 1H), 1.09–0.97 (m, 5H). ^13^C NMR (101 MHz, CDCl_3_) δ 192.29 (t, *J* = 35.1 Hz), 168.66, 151.40, 142.36, 140.94, 135.37, 130.65, 130.42, 128.69, 127.01, 126.53, 119.59, 117.92 (dd, *J* = 249.9 Hz), 116.31, 114.28, 60.16, 56.23, 49.72, 40.47, 36.52, 25.48 (t, *J* = 23.1 Hz), 17.83, 12.61, 5.43 (t, *J* = 5.3 Hz). HRMS: Calcd for [C_24_H_26_Cl_2_F_2_N_2_O_3_S + H_2_O + H]^+^ = 549.1188, found = 549.1187.

#### 1-((2-Chloro-4-(4-(3-chlorophenyl)-trans-2,3-dimethylpiperazine-1-carbonyl)phenyl)sulfinyl)-3,3-difluoropentan-2-one (**(−)73**)

The title compound was synthesized using (−)-(3-chloro-4-(methylsulfinyl)phenyl)(trans-4-(3-chlorophenyl)-2,3-dimethylpiperazin-1-yl)methanone (20 mg, 47 μmol, 1 equiv) and ethyl 2,2-difluorobutanoate (72 mg, 0.47 mmol, 10 equiv) according to general procedure C. This yielded the product (11 mg, 21 μmol, 44%). [α]^20^_D_ = −55.4. ^13^C NMR (101 MHz, CDCl_3_) δ 192.29 (t, *J* = 35.1 Hz), 168.62, 151.41, 142.36, 140.88, 135.34, 130.57, 130.40, 128.56, 126.95, 126.43, 119.47, 117.89 (dd, *J* = 249.9 Hz), 116.21, 114.16, 60.07, 56.11, 49.65, 40.53, 36.58, 25.52 (t, *J* = 23.1 Hz), 17.79, 12.59, 5.39 (t, *J* = 5.3 Hz). HRMS: Calcd for [C_24_H_26_Cl_2_F_2_N_2_O_3_S + H_2_O + H]^+^ = 549.1188, found = 549.1187.

#### 3-((2-Chloro-4-(4-(3-chlorophenyl)-trans-2,3-dimethylpiperazine-1-carbonyl)phenyl)sulfinyl)-1,1-difluoro-1-phenylpropan-2-one (**±74**)

The title compound was synthesized using (±) (3-chloro-4-(methylsulfinyl)phenyl)(trans-4-(3-chlorophenyl)-2,3-dimethylpiperazin-1-yl)methanone (40 mg, 0.09 mmol, 1 equiv) and ethyl 2,2-difluoro-2-phenylacetate (188 mg, 0.94 mmol, 10 equiv) according to general procedure C. This yielded the product (13.4 mg, 0.02 mmol, 25%). ^1^H NMR (600 MHz, CDCl_3_) δ 7.95 (d, *J* = 7.9 Hz, 1H), 7.63–7.42 (m, 7H), 7.17 (t, *J* = 8.1, 2.4 Hz, 1H), 6.85–6.78 (m, 2H), 6.74–6.68 (m, 1H), 4.69–4.32 (m, 1H), 4.07–3.01 (m, 5H), 1.54–1.41 (m, 3H), 1.17–0.97 (m, 3H). ^13^C NMR (151 MHz, CDCl_3_) δ 191.69, 191.44, 152.19, 143.10, 141.58, 136.27, 132.65 (t), 131.65, 131.31, 131.18, 130.07, 129.20, 128.43, 128.03, 126.86 (t), 120.59, 117.27, 116.39 (t), 115.24, 61.07, 61.04, 57.17, 56.53, 30.71, 13.74, 1.01. HRMS: Calcd for [C_28_H_26_Cl_2_F_2_N_2_O_3_S + H_2_O + H]^+^ = 597.1188, found = 597.1189.

#### 1-((2-Chloro-4-(4-(6-chloropyridin-2-yl)-trans-2,3-dimethylpiperazine-1-carbonyl)phenyl)sulfinyl)-3,3-difluoropentan-2-one (**±75**)

The title compound was synthesized using (±) (3-chloro-4-(methylsulfinyl)phenyl)(4-(6-chloropyridin-2-yl)-trans-2,3-dimethylpiperazin-1-yl)methanone (33 mg, 0.08 mmol, 1.0 equiv) and ethyl 2,2-difluorobutanoate (118 mg, 0.77 mmol, 10 equiv) according to general procedure C. This yielded the product (18 mg, 0.02 mmol, 29%). ^1^H NMR (500 MHz, CDCl_3_) δ 8.16 (d, *J* = 5.5 Hz, 1H), 8.04 (d, *J* = 8.0 Hz, 1H), 7.62–7.54 (m, 1H), 7.54–7.41 (m, 1H), 6.79 (d, *J* = 12.3 Hz, 2H), 4.93–4.60 (m, 1H), 4.44–4.35 (m, 1H), 4.28–3.95 (m, 2H), 3.80–3.67 (m, 1H), 3.67–3.50 (m, 1H), 3.45–3.21 (m, 1H), 2.21–1.99 (m, 1H), 1.47–1.31 (m, 5H), 1.28–1.17 (m, 2H), 1.05 (t, *J* = 7.5 Hz, 3H). HRMS: Calcd for [C_23_H_25_Cl_2_F_2_N_3_O_3_S + H_2_O + H]^+^ = 550.1140, found = 550.1141.

## Supporting Information Available

Significant positive correlation between pIC_50_ values and *c*Log *P;* synthesis of **(+)-73** and **(−)-73**; synthesis of the benzoic acid and amine intermediates; synthesis of the final inhibitors; synthetic procedures for the synthesis of all intermediates. ^1^H and ^13^C NMR spectra for **±73** (**LEI-515**) and HPLC traces for **10**, **14**, (*R*)**-25**, **27**, **34**, **±43**, **±55,** and **±73 (LEI-515)**; and chiral HPLC spectra of enantiomerically enriched sulfoxide intermediates (PDF)
Molecular formula strings (CSV)
PDB ID Code: 8AQF^28^

## Abbreviations

2-AG: 2-arachidonyl glycerol
AA: arachidonic acid
Cl_int_: intrinsic clearance
CNS: central nervous system
DiPEA: *N,N*-diisopropylethylamine
GK: glycerol kinase
GPO: glycerolphosphate oxidase
HATU: hexafluorophosphate azabenzotriazole tetramethyl uronium
HRP: horse radish peroxide
HTS: high throughput screening
LDA: lithium diisopropylamide
LipE: lipophilic efficiency
MAGL: monoacylglycerol lipase
