# Supplementary material for: Structure–Activity Relationship Studies of Aryl Sulfoxides as Reversible Monoacylglycerol Lipase Inhibitors
Source: J Med Chem. 2024 Jul 11;67(14):12331–48. doi: 10.1021/acs.jmedchem.4c01037 (PMC11284796; doi:10.1021/acs.jmedchem.4c01037)
Supplement: Supplementary file 1 — jm4c01037_si_001.pdf [file jm4c01037_si_001.pdf]

Supporting Information

Structure-activity relationship studies of aryl sulfoxides as reversible monoacylglycerol lipase inhibitors

Ming Jiang[1], Mirjam C. W. Huizenga[1], Florian Mohr[1], Avand Amedi[1], Renze Bakker, Richard J. B. H. N. van den Berg[2], Hui Deng[1], Tom van der Wel[1], Constant A.A. van Boeckel[1], Mario van der Stelt[1]*

[1]Department of Molecular Physiology, Leiden University & Oncode Institute, 2333 CC, Netherlands;

[2]Department of Bio-organic Synthesis, Leiden University, 2333 CC, Netherlands.

Email: m.van.der.stelt@chem.leidenuniv.nl

Contents **Page**

Contents	Page
Figure S1	2
Scheme S1-7	3
Synthetic procedures	8
^{1}H and ^{13}C NMR spectra for ±73 (LEI-515)	47
HPLC-traces for **10**, **14**, **(R)-25**, **27**, **34**, **±43**, **±55** and **±73 (LEI-515)**	48
Chiral HPLC spectra of enantiomerically enriched sulfoxide intermediates ((+)-136 and (-)-136)	51

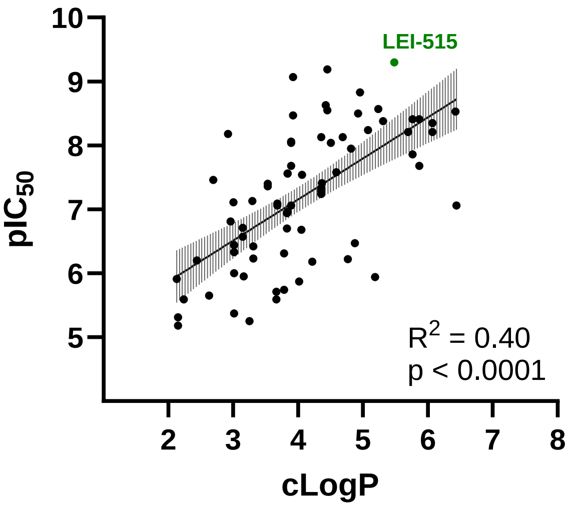

Figure S1 – Significant positive correlation between pIC_{50} values and cLogP. LipE = pIC_{50} – cLogP.

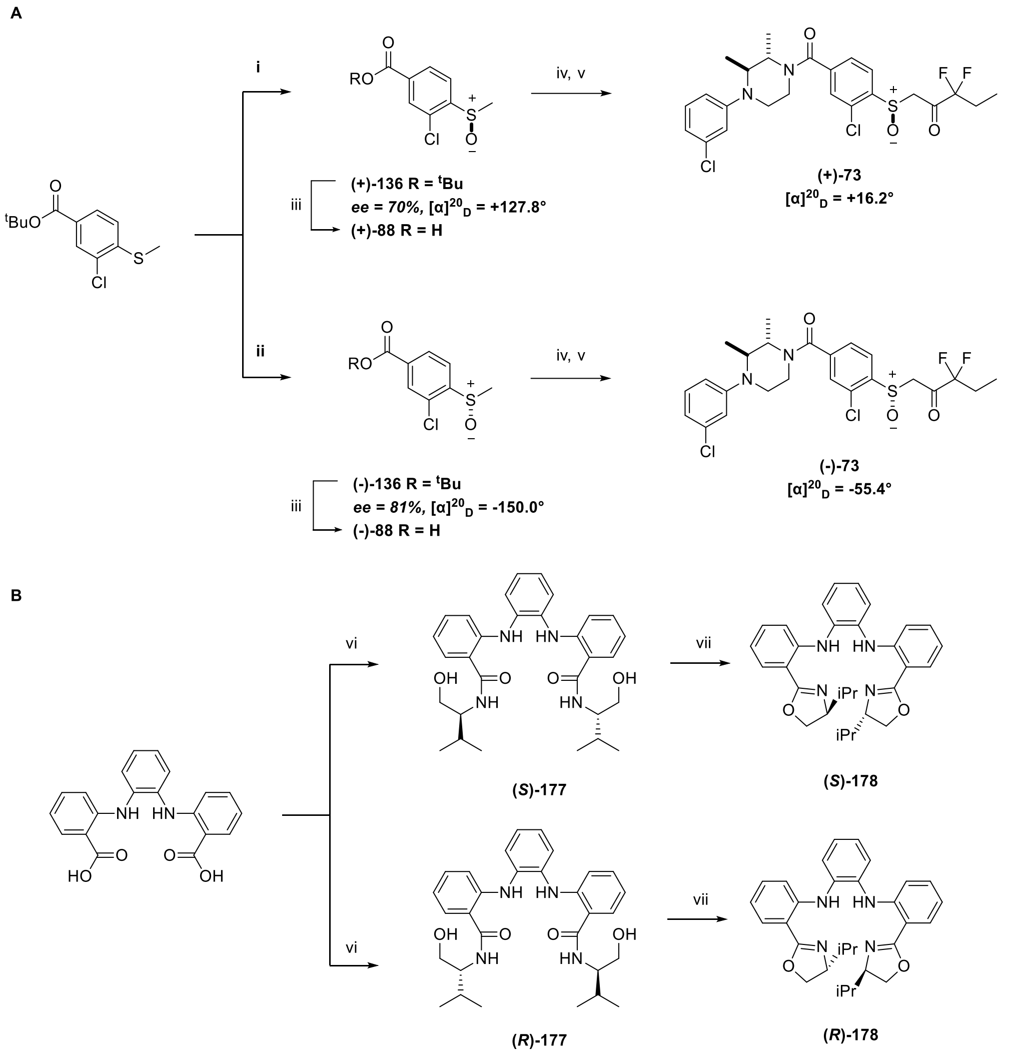

Scheme S1 – Synthesis route of compounds **(+)-73** and **(-)-73**. Reagents and conditions: i) **(*S*)-178**, $Mn(OTf)_2$, AcOH, H_2O_2, DCM, RT → 0°C, ee = 70%; ii) **(*R*)-178**, $Mn(OTf)_2$, AcOH, H_2O_2, DCM, RT → 0 °C, ee = 81%; iii) TFA, DCM, 85%-quant.; iv) **±115**, HATU, DiPEA, DCM; v) LDA, ethyl 2,2-difluorobutanoate, THF, -94 °C; vi) (*S* or *R*)-2-amino-3-methylbutan-1-ol, EDC, HOBt, DiPEA, DCM; vii) PPh_3, Et_3N, CCl_4, ACN.

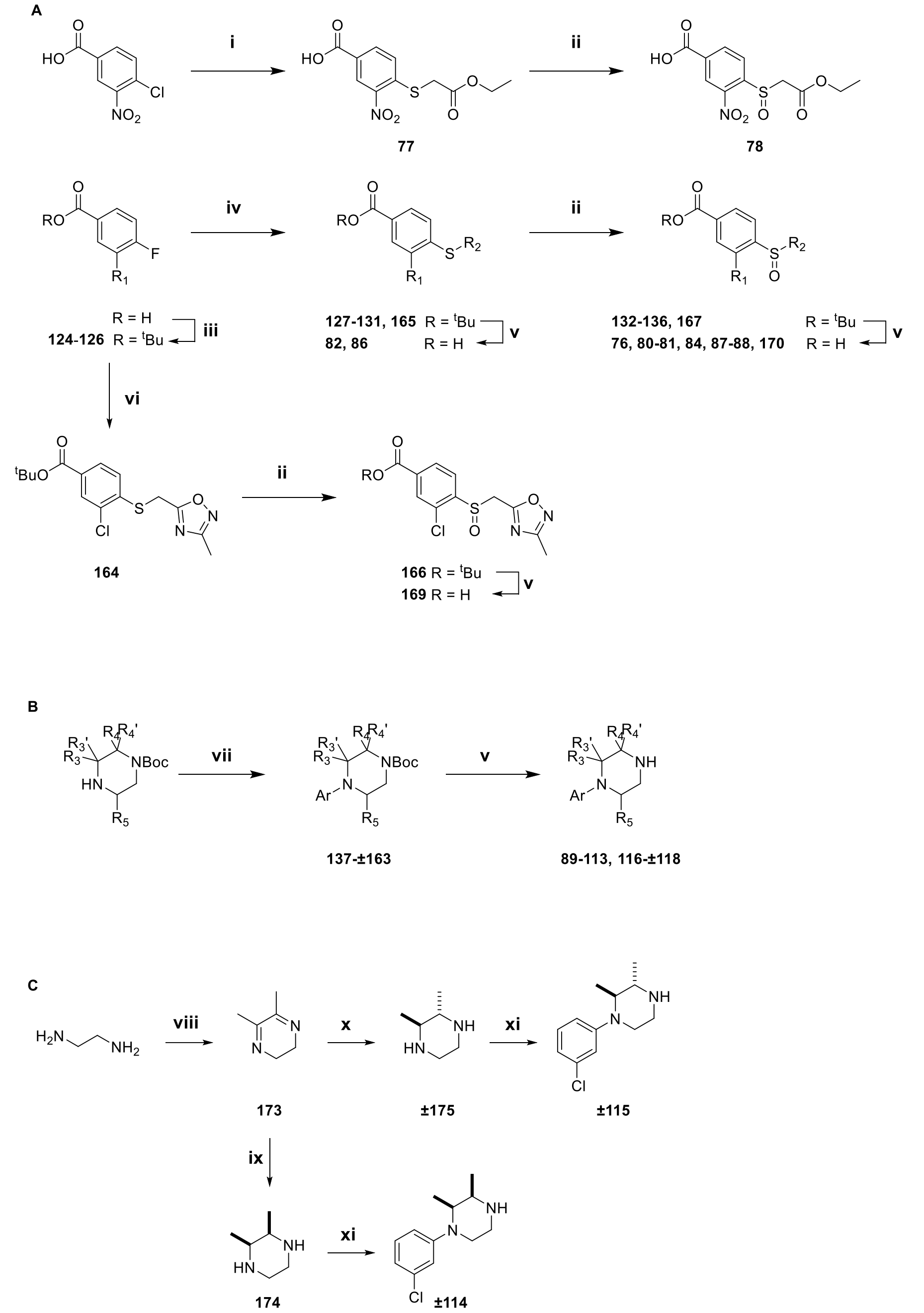

Scheme S2 – Synthesis route of benzoic acid (**A**) and piperazine (**B-C**) intermediates. Reagents and conditions: i) ethyl 2-mercaptoacetate, pyridine, 115 °C; ii) Oxone, MeOH, H_2O, rt; iii) Boc_2O, DMAP, *tert*-BuOH, 65 °C; iv) appropriate thiol, K_2CO_3, ACN, rt; v) TFA, DCM, rt; vi) NaSH, 5-(chloromethyl)-3-methyl-1,2,4-oxadiazole, DMF, rt; vii) sodium *tert*-butoxide, BINAP, $Pd(OAc)_2$, 1,4-dioxane, 85 °C; viii) 2,3-butadione, Et_2O, rt; ix) Pd/C, H_2, EtOH, 0 °C - rt; x) Na, EtOH, reflux; xi) KHMDS, dioxane, rt.

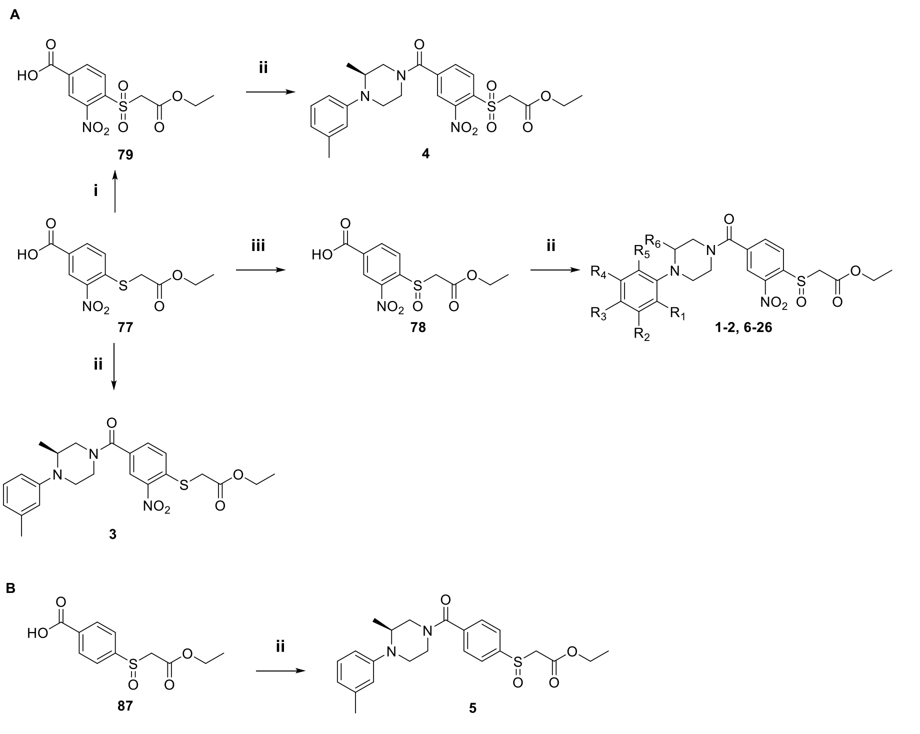

Scheme S3 – Synthesis route of compounds **1-26**. Reagents and conditions: i) H_2O_2, tetrabutylammonium hygrogen sulfate, Na_2WO_4 $2H_2O$, AcOH, reflux; ii) HATU, DiPEA, the appropriate phenylpiperazine, DCM, rt; iii) Oxone, MeOH, H_2O, 0 °C – rt.

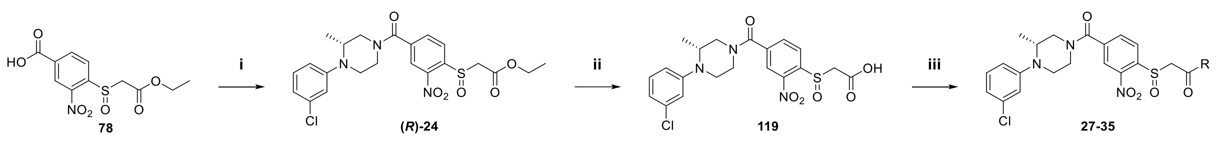

Scheme S4 – Synthesis route of compounds **27-36**. Reagents and conditions: i) HATU, DiPEA, **(*R*)-109**, DCM, rt; ii) TEA, H_2O, MeOH, rt; iii) oxalyl chloride, DiPEA, appropriate alcohol or *N*-methylethanamine, DCM, 0 °C – rt.

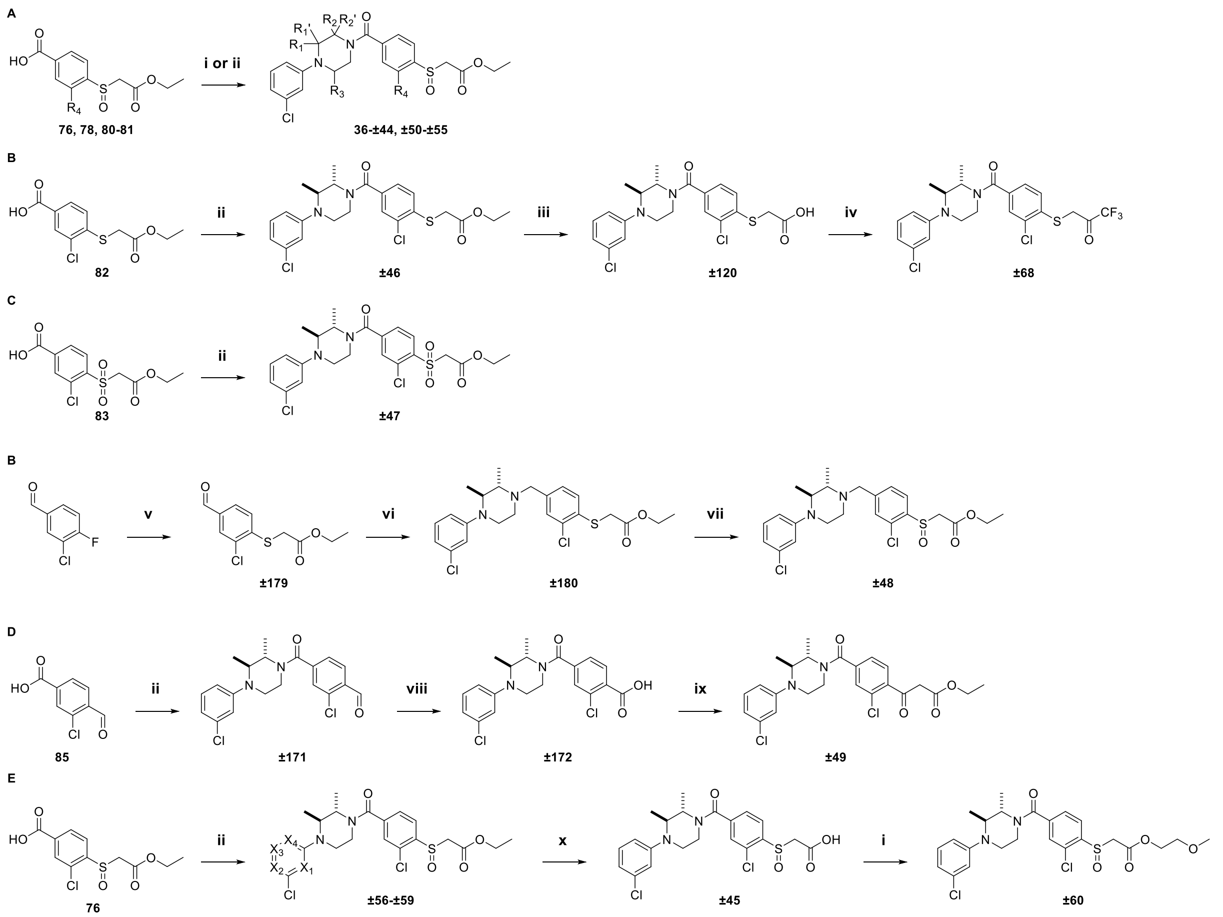

Scheme S5 – Synthesis route of compounds **36-±60**, **±68** and **±120**. Reagents and conditions: i) oxalyl chloride, DiPEA, appropriate alcohol or amine, DCM, 0 °C – rt; ii) HATU, DiPEA, appropriate phenylpiperazine, DCM, rt; iii) NaOH, MeOH, rt; iv) TFAA, pyridine, toluene, 0 °C – 65 °C; v) ethyl 2-mercaptoacetate, K_2CO_3, DMF, rt; vi) **±115**, $NaBH(OAc)_3$, 1,2-dichloroethane, rt; vii) Oxone, MeOH/H_2O, 0 °C-rt; viii) Oxone, DMF, rt; ix) carbonyldiimidazole, ethyl potassium malonate, TEA, THF, rt; x) TEA, H_2O, MeOH, rt.

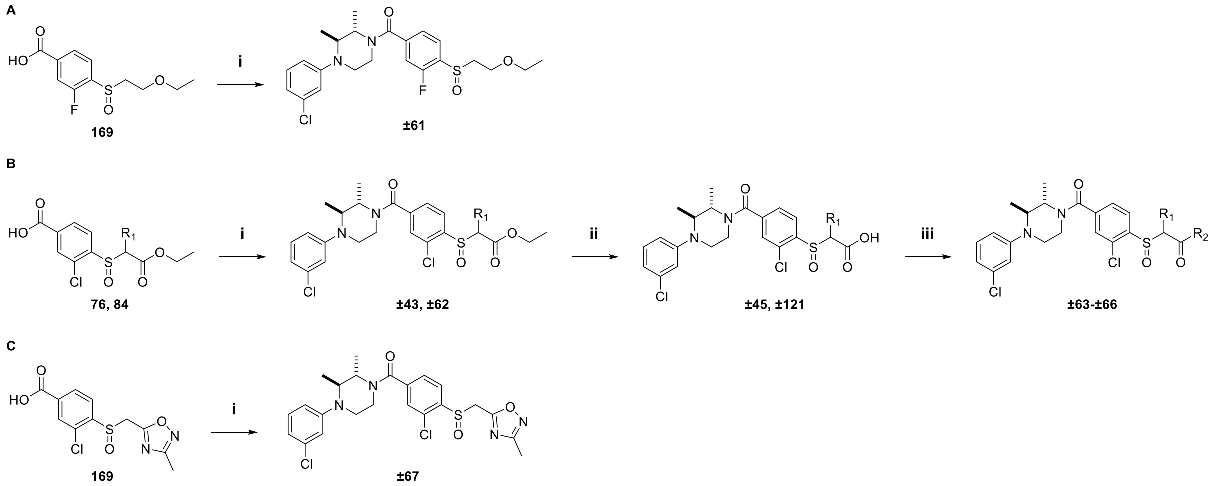

Scheme S6 – Synthesis route of compounds **±61-±67**. Reagents and conditions: i) HATU, DiPEA, **±115**, DCM, rt; ii) TEA, H_2O, MeOH, rt; iii) oxalyl chloride, DiPEA, appropriate alcohol or ethylamine, DCM, 0 °C – rt.

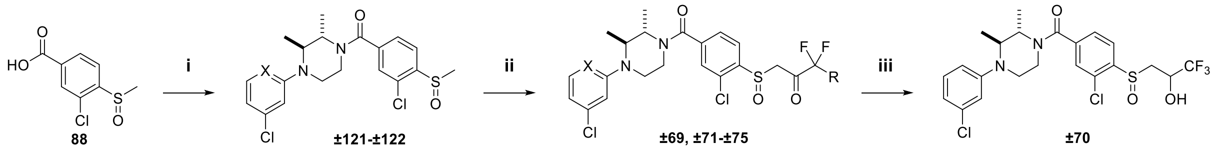

Scheme S7 – Synthesis route of compounds **±69-±75**. Reagents and conditions: i) HATU, DiPEA, **±115** or **±118**, DCM, rt; ii) LDA, the appropriate ethyl ester, THF, -78 °C – rt; iii) $NaBH_4$, MeOH, 0 °C.

Chemistry procedures

General remarks. All reactions were performed using oven or flame-dried glassware and analytical grade solvents that were dried with molecular sieves. Reagents were purchased from Sigma Aldrich, Acros and Merck and used without further purification unless noted otherwise. All moisture sensitive reactions were performed under an argon or nitrogen atmosphere. Traces of water were removed from starting compounds by co-evaporation with toluene. Reactions were followed by thin layer chromatography and was performed using TLC Silica gel 60 F_{245} on aluminums sheets. Compounds were visualized using a $KMnO_4$ stain (K_2CO_3 (66 mg/mL), $KMnO_4$ (10 mg/mL) in 0.1 % NaOH. Amines were visualized using ninhydrin (0.75 g/L) and acetic acid (12.5 mL/L) in ethanol. ^{1}H- and ^{13}C-NMR spectra were recorded on a Bruker AV-400, 500, 600 or 850 using $CDCl_3$ or CD_3OD as solvent, unless stated otherwise. Chemical shift values are reported in ppm with tetramethylsilane or solvent resonance as the internal standard ($CDCl_3$: δ 7.26 for ^{1}H, δ 77.16 for ^{13}C, CD_3OD: δ 3.31 for ^{1}H, δ 49.00 for ^{13}C). Data are reported as follows: chemical shifts (δ) in ppm, multiplicity (s = singlet, d = doublet, dd = double doublet, td = triple doublet, t = triplet, q = quartet, quintet = quint, br = broad, m = multiplet), coupling constants *J* (Hz), and integration. LC-MS measurements were performed on a Thermo Finnigan LCQ Advantage Max ion-trap mass spectrometer (ESI^+) coupled to a Surveyor HPLC system (Thermo Finnigan) equipped with a standard C18 (Gemini, 4.6 mmD × 50 mmL, 5 µm particle size, Phenomenex) analytical column and buffers A: H_2O, B: ACN, C: 0.1% aq. TFA. Preparative HPLC purification was performed on a Waters Acquity Ultra Performance LC with a C18 column (Gemini, 150 × 21.2 mm, Phenomenex). Diode detection was done between 210 and 600 nm. Gradient: ACN in (H_2O + 0.2% TFA). High-resolution mass spectra (HRMS) were recorded on a Thermo Scientific LTQ Orbitrap XL. Specific rotations of intermediates are provided in degrees (°) and were measured by Anton Paar® MCP100 polarimeter at 20 °C using wavelength of 589 nm and a cuvette length of 10 cm in chloroform (1 mL), "(+)" prefix indicates *dextro*-rotation and "(-)" indicates *levo*-rotation. Enantiomeric excess (*ee*) was measured using Chiral shell HPLC and Chiralcel OD stationary phase (4.6 × 250mm, 1 mL/min), racemates were used as reference. All final compounds were determined to be >95% pure by integrating UV intensity recorded via HPLC.

General procedure A

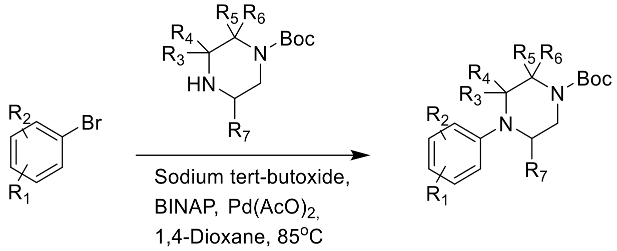

A mixture of the appropriate bromobenzene (1 eq.), mono Boc-protected piperazine (1 eq.), palladium diacetate (0.04 eq.), BINAP (0.06 eq.) and sodium *tert*-butoxide (1.5 eq.) in degassed 1,4-dioxane (0.4 M) was heated to 85 °C under nitrogen atmosphere overnight. The reaction progress was monitored by TLC analysis. Upon full conversion of the starting materials, the mixture was diluted with DCM, washed with water, dried ($MgSO_4$), filtered and concentrated under reduced pressure. The residue was purified by silica gel column chromatography (pentane/Et_2O) to give the product.

General procedure B

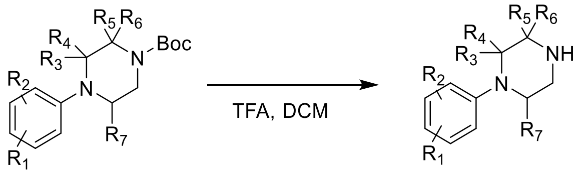

To a solution of the appropriate *tert*-butyl phenylpiperazine-1-carboxylate in DCM was added TFA (0.4 M, final TFA concentration, 20% *v/v*) and the mixture was stirred at room temperature for 2h. The reaction progress was monitored by TLC analysis. Upon full conversion of the starting materials, the mixture was diluted with DCM and washed with saturated aqueous $NaHCO_3$. The organic layer was dried ($MgSO_4$), filtered and concentrated under reduced pressure. The residue was purified by silica gel column chromatography (DCM/MeOH) to give the product.

General procedure C

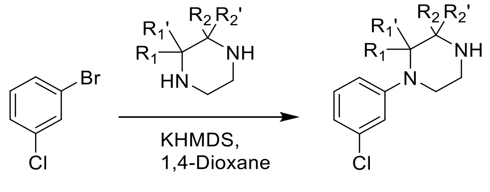

To a solution of appropriate piperazine (1 eq.) and bromochloropyridine (1 eq.) in anhydrous 1,4-dioxane (0.3M) was added KHMDS (1.5 eq.) and the mixture was stirred overnight at RT. The reaction progress was monitored by TLC analysis. Once completed, the reaction mixture was diluted with DCM,

washed with water and dried over anhydrous $MgSO_4$. After filtration, the filtrate was concentrated under reduced pressure. The residue was purified by silica gel column chromatography.

General procedure D

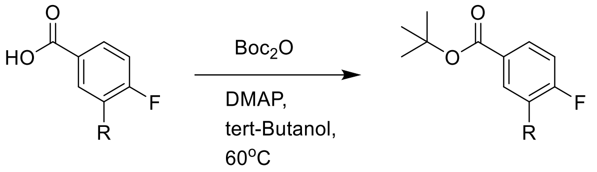

A mixture of the appropriate benzoic acid (1 eq.), di-*tert*-butyl dicarbonate (3 eq.) and DMAP (0.3 eq.) in *tert*-butanol (0.4 M) was heated to 60 °C and stirred overnight. The reaction progress was monitored by TLC analysis. Upon full conversion of the starting meterials, the solvent was evaporated and the residue was purified by silica gel column chromatography (pentane/diethyl ether) to give the product.

General procedure E

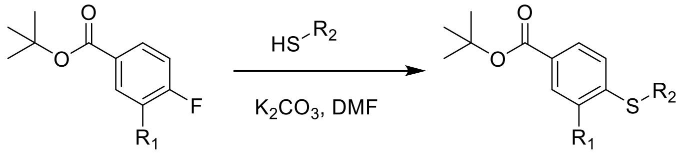

To a solution of the appropriate *tert*-butyl 4-fluorobenzoate (1.2 eq.) in ACN (0.4 M) were added K_2CO_3 (3 eq.) and the appropriate thiol (1 eq.). The reaction mixture was stirred at RT overnight. The reaction progress was monitored by TLC analysis. Upon full conversion of the starting materials, the mixture was diluted with Et_2O and washed with water, dried ($MgSO_4$). After filtration, the filtrate was concentrated under reduced pressure. The residue was purified by silica gel column chromatography (pentane/diethyl ether).

General procedure F

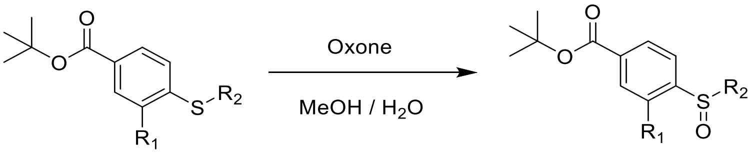

To a cooled (0 °C) solution of the appropriate thioether (1 eq.) in MeOH (0.15 M) was dropwise added an Oxone in water solution (0.25 M) and the mixture was stirred at RT for 2h. The reaction progress was monitored by TLC analysis. Upon full conversion of the starting materials, the mixture was diluted

with EtOAc and washed with water. The organic layer was dried ($MgSO_4$), filtered and concentrated under reduced pressure. The residue was purified by silica column chromatography (pentane/EtOAc).

General procedure G

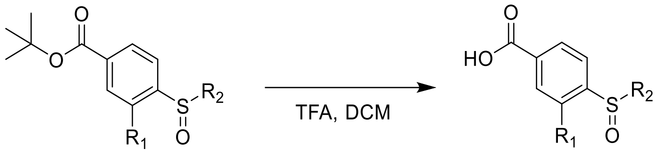

To a solution of the appropriate *tert*-butyl protected carboxylic acid in DCM was added TFA (0.4 M, final TFA concentration, 20% *v/v*) and the mixture was stirred at RT for 6 h. The reaction progress was monitored by TLC analysis. Upon full conversion of the starting materials, the solvent was removed under reduced pressure and the residue was purified silica gel column chromatography (DCM/MeOH).

General procedure H

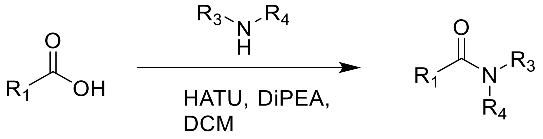

To a suspension or solution of the appropriate benzoic acid (1 eq.) in DCM (0.4 M) was added HATU (1.5 eq.) and DiPEA (3 eq.) and then the mixture was stirred at room temperature for 1h. The appropriate phenylpiperazine (1eq.) was added and the mixture was stirred overnight. The reaction progress was monitored by TLC analysis. Upon full conversion of the starting materials, the mixture was diluted with DCM and washed with water, dried ($MgSO_4$), filtered and concentrated under reduce pressure. The residue was purified by silica gel column chromatography (pentane/EtOAc) or HPLC-MS.

3-Chloro-4-((2-ethoxy-2-oxoethyl)sulfinyl)benzoic acid (**76**)

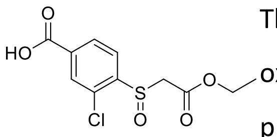

The title compound was synthesized using *tert*-butyl 3-chloro-4-((2-ethoxy-2-oxoethyl)sulfinyl)benzoate (1.39 g, 4.00 mmol, 1 eq.) according to general procedure G in a yield of 1.04 g (3.59 mmol, 90%). ^{1}H NMR (500 MHz, $CDCl_3$) δ 8.17 (dd, *J* = 8.1, 1.5 Hz, 1H), 8.07 (d, *J* = 1.5, 1H), 7.99 (d, *J* = 8.1, 1H), 4.26 – 4.14 (m, 2H), 4.04 (d, *J* = 14.0 Hz, 1H), 3.72 (d, *J* = 14.0 Hz, 1H), 1.23 (t, *J* = 7.1 Hz, 3H). ^{13}C NMR (126 MHz, $CDCl_3$) δ 166.5, 164.5, 145.4, 135.0, 131.2, 130.2, 129.3, 126.7, 62.5, 58.0, 14.1.

4-((2-Ethoxy-2-oxoethyl)thio)-3-nitrobenzoic acid (**77**)

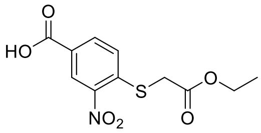

To a solution of 4-chloro-3-nitrobenzoic acid (1.26 mg, 6.24 mmol, 1.50 eq.) in pyridine (5 mL) was added ethyl mercaptoacetate (0.5 g, 4.16 mmol, 1.00 eq.) and the mixture was heated to 115 ^{o}C overnight in an oil bath. The reaction progress was monitored by TLC analysis. Upon full conversion of the starting material, the mixture was allowed to cool to rt and the pH was adjusted to 1 with 1M HCl solution. The precipitate was filtered and the solid were washed with water to provide the product (1.01 g, 3.54 mmol, 85%). ^{1}H NMR (400 MHz, Methanol-*d*4) δ 8.73 (d, *J* = 1.9 Hz, 1H), 8.16 (dd, *J* = 8.5, 1.9 Hz, 1H), 7.66 (d, *J* = 8.5 Hz, 1H), 4.20 (q, *J* = 7.1 Hz, 2H), 4.00 (s, 2H), 1.25 (t, *J* = 7.1 Hz, 3H). ^{13}C NMR (101 MHz, Methanol-*d*4) δ 170.38, 167.34, 143.24, 135.02, 129.44, 128.46, 128.08, 63.20, 35.65, 14.53.

4-((2-Ethoxy-2-oxoethyl)sulfinyl)-3-nitrobenzoic acid (78)

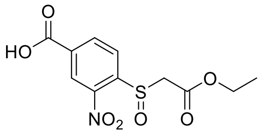

To a cooled solution of 4-((2-ethoxy-2-oxoethyl)thio)-3-nitrobenzoic acid (285 mg, 1.00 mmol, 1.00 eq.) in methanol (13mL) was dropwise added a solution of Oxone (62 mg, 1.00 mmol, 1.00 eq.) in water (4 mL) and the reaction mixture was stirred at rt for 2.5 h. The reaction progress was monitored by TLC analysis. Upon full conversion of the starting materials, the mixture was diluted with water and extracted with DCM. The combined organic layers were washed with brine, dried ($MgSO_4$), filtered and concentrated under reduced pressure. The residue was purified by silica gel column chromatography (MeOH/DCM, 1%→2%) to afford the product (210 mg, 0.70 mmol, 70%). ^{1}H NMR (400 MHz, Methanol-*d*4) δ 8.88 (d, *J* = 1.6 Hz, 1H), 8.61 (dd, *J* = 8.2, 1.6 Hz, 1H), 8.34 (d, *J* = 8.2 Hz, 1H), 4.36 (d, *J* = 14.4 Hz, 1H), 4.29 – 4.13 (m, 2H), 3.82 (d, *J* = 14.5 Hz, 1H), 1.26 (t, *J* = 7.1 Hz, 3H). ^{13}C NMR (101 MHz, Methanol-*d*4) δ 166.82, 166.60, 147.30, 136.89, 136.55, 128.43, 127.26, 63.37, 61.32, 14.54.

4-((2-Ethoxy-2-oxoethyl)sulfonyl)-3-nitrobenzoic acid (79)

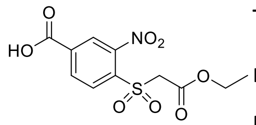

To a solution of 4-((2-ethoxy-2-oxoethyl)thio)-3-nitrobenzoic acid (1.10 g, 3.86 mmol, 1.00 eq.) in AcOH (20 mL) was added H_2O_2 (35%, 5 mL), *tert*-butylammonium hydrogen sulfate (65 mg, 0.19 mmol, 0.05 eq.) and sodium tungstate dihydrate (127 mg, 0.39 mmol, 0.1 eq.) and the reaction mixture was refluxed for 3h. The reaction progress was monitored by TLC analysis. Upon full conversion of the starting materials, the mixture was cooled down to rt and diluted with water, extracted with EtOAc and dried ($MgSO_4$), filtered and concentrated under reduced pressure. The residue was purified by silica gel column chromatography (MeOH/DCM, 1%→2%) to afford the product (0.62g, 1.95 mmol, 51%). ^{1}H NMR (400 MHz, DMSO-*d*6) δ 8.51 (d, *J* = 1.6 Hz, 1H), 8.43 (dd, *J* = 8.2, 1.7 Hz, 1H), 8.26 (d, *J* = 8.2 Hz, 1H), 4.93 (s, 2H), 4.10 (q, *J* = 7.1 Hz, 2H), 1.10 (t, *J* = 7.1 Hz, 3H). ^{13}C NMR (101 MHz, DMSO-*d*6) δ 164.46, 162.06, 148.41, 137.81, 134.35, 133.34, 133.28, 125.63, 62.09, 60.66, 13.71.

4-((2-Ethoxy-2-oxoethyl)sulfinyl)-3-fluorobenzoic acid (80)

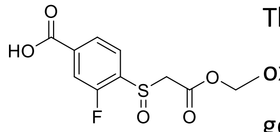

The title compound was synthesized using *tert*-butyl 4-((2-ethoxy-2-oxoethyl)sulfinyl)-3-fluorobenzoate (30 mg, 0.09 mmol, 1 eq.) according to general procedure G in a yield of 18 mg (0.7 mmol, 72%). ^{1}H NMR (400 MHz, Methanol-*d*4) δ 8.10 (dd, *J* = 8.1, 1.5 Hz, 1H), 7.95 – 7.80 (m, 2H), 4.27 – 4.05 (m, 3H), 3.95 (d, *J* = 14.2 Hz, 1H), 1.19 (t, *J* = 7.1 Hz, 3H). ^{13}C NMR (101 MHz, Methanol-*d*4) δ 167.20, 165.75, 159.03 (d, *J* = 247.7 Hz), 137.86 (d, *J* = 7.1 Hz), 135.69 (d, *J* = 16.9 Hz), 127.66 (d, *J* = 3.3 Hz), 127.53 (d, *J* = 2.1 Hz), 117.91 (d, *J* = 22.2 Hz), 63.17, 59.38, 14.31.

3-Bromo-4-((2-ethoxy-2-oxoethyl)sulfinyl)benzoic acid (81)

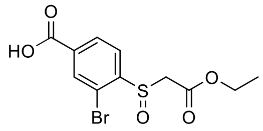

The title compound was synthesized using *tert*-butyl 3-bromo-4-((2-ethoxy-2-oxoethyl)sulfinyl)benzoate (167 mg, 0.43 mmol, 1 eq.) according to general procedure G in a yield of 60 mg (0.18 mmol, 43 %). ^{1}H NMR (300 MHz, Methanol-*d*4) δ 8.32 – 8.12 (m, 2H), 7.96 (d, *J* = 8.5 Hz, 1H), 4.22 – 4.18(m, 3H), 3.86 (d, *J* = 14.3 Hz, 1H), 1.31 – 1.10 (m, 3H). ^{13}C NMR (75 MHz, Methanol-*d*4) δ 165.64, 164.61, 146.54, 135.50, 133.81, 129.35, 126.54, 118.38, 61.85, 57.72, 12.98.

3-Chloro-4-((2-ethoxy-2-oxoethyl)thio)benzoate (82)

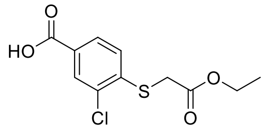

The title compound was synthesized using *tert*-butyl 3-chloro-4-((2-ethoxy-2-oxoethyl)thio)benzoate (61.5 mg, 0.19 mmol) according to procedure G. This yielded the product (51.1 mg, 0.19 mmol, 92%). ^{1}H NMR (400 MHz, $CDCl_3$) δ 8.07

(s, 1H), 7.95 (d, *J* = 8.3 Hz, 1H) , 7.37 (d, *J* = 8.2 Hz, 1H), 4.23 (q, *J* = 7.2 Hz, 2H), 3.78 (s, 2H), 1.28 (t, *J* = 7.1 Hz, 3H). ^{13}C NMR (400 MHz, $CDCl_3$) δ 170.34, 168.37, 142.67, 130.76, 128.58, 127.15, 125.87, 61.98, 34.03, 13.91.

3-chloro-4-((2-ethoxy-2-oxoethyl)sulfonyl)benzoate (**83**)

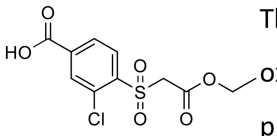

The title compound was synthesized using *tert*-butyl 3-chloro-4-((2-ethoxy-2-oxoethyl)sulfonyl)benzoate (65 mg, 0.18 mmol, 1 eq.) according to general procedure G in a yield of 50.2 mg (0.16 mmol, 91%). ^{1}H NMR (400 MHz, Methanol-*d*4) δ 8.28 – 8.05 (m, 3H), 4.61 (s, 2H), 4.07 (q, *J* = 7.1 Hz, 2H), 1.08 (t, *J* = 7.1 Hz, 3H). ^{13}C NMR (101 MHz, Methanol-*d*4) δ 165.24, 162.16, 139.79, 137.42, 132.58, 132.39, 131.98, 128.13, 61.91, 58.48, 12.67.

3-Chloro-4-((1-ethoxy-1-oxopropan-2-yl)sulfinyl)benzoic acid (**84**)

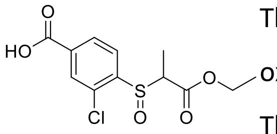

The title compound was synthesized using *tert*-butyl 3-chloro-4-((2-ethoxy-2-oxoethyl)sulfinyl)benzoate (0.14 g, 0.40 mmol, 1 eq.) according to procedure G. This yielded the product (0.12 g, 0.40 mmol, 100%). ^{1}H NMR (400 MHz, Methanol-*d*4): δ 8.22 (dd, *J* = 8.1, 1.5 Hz, 1H), 8.11 (d, *J* = 1.5 Hz, 1H), 7.89 (d, *J* = 8.1 Hz, 1H), 4.30 (qd, *J* = 7.1, 2.3 Hz, 2H), 4.13 (q, *J* = 7.1 Hz, 1H), 1.32 (t, *J* = 7.1 Hz, 3H), 1.21 (d, *J* = 7.1 Hz, 3H). ^{13}C NMR (101 MHz, Methanol-*d*4): δ 169.70, 144.80, 136.92, 132.16, 131.70, 129.93, 128.61, 63.50, 61.67, 48.58, 14.42, 6.78.

3-Chloro-4-formylbenzoic acid (85)

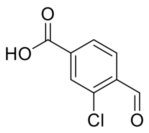

To a cooled (-78 °C) solution of 4-bromo-3-chlorobenzoic acid (300 mg, 1.27 mmol, 1.00 eq.) in THF (5.0 mL) was added Turbo-Grignard (1.3 M in THF, 2.94 ml, 3.82 mmol, 3.00 eq.). The temperature was raised to 0 °C after 10 min and the reaction mixture was stirred at 0 °C for 1 h. Subsequently DMF (6.37 ml, 0.49 mmol, 5.00 eq.) was added, the reaction mixture warmed up to room temperature and stirred further for 1.5 h. The reaction was quenched by addition of sat. aq. NH_4Cl, followed by washing with EtOAc. Then the pH of the aqueous layer was adjusted (pH 2) with aq. 1 N HCl and extracted with EtOAc. The combined organic layers were neutralized with sat. aq. $NaHCO_3$, washed with brine, dried (Na_2SO_4) and concentrated under reduced pressure. The title compound was used for the next step without any further purification (165 mg, 0.89 mmol, 70%). ^{1}H NMR (500 MHz, DMSO-*d*6) δ 10.38 (s, 1H), 8.07 – 8.00 (m, 2H), 8.01 – 7.95 (m, 1H). ^{13}C NMR (126 MHz, DMSO-*d*6) δ 189.6, 165.4, 136.8, 136.1, 134.8, 131.1, 130.1, 128.3.

4-((2-Ethoxy-2-oxoethyl)thio)benzoic acid (**86**)

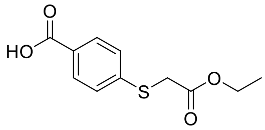

To a suspension of 4-mercaptobenzoic acid (0.46 g, 2.99 mmol, 1.00 eq.) in water (5mL) was added NaOH (0.18 g, 4.49 mmol, 1.50 eq.) and the resulting solution was stirred for 30 min at room temperature. Then ethyl 2-bromoacetate (0.50 g, 2.99 mmol, 1.00 eq.) was slowly added to the solution and the reaction mixture was stirred for another 2h at rt. The reaction progress was monitored by TLC analysis. Upon full conversion of the starting materials, the mixture was acidified with 1M HCl and the obtained precipitate was filtered, washed with water and dried to give the product (252 mg, 1.05 mmol, 35 %). ^{1}H NMR ($CDCl_3$, 400 MHz) δ 7.88 (d, *J* = 8.3 Hz, 2H), 7.40 – 7.26 (d, *J* = 8.3 Hz, 2H), 4.10 (q, *J* = 7.3 Hz, 2H), 3.79 (s, 2H), 1.15 (t, *J* = 7.2 Hz, 3H). ^{13}C NMR (Methanol-*d*4, 101 MHz) δ 170.94, 169.28, 143.74, 131.16, 129.19, 127.98, 62.72, 35.35, 14.37.

4-((2-Ethoxy-2-oxoethyl)sulfinyl)benzoic acid (**87**)

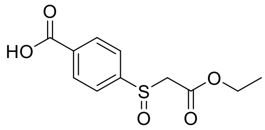

To a cooled solution of 4-((2-ethoxy-2-oxoethyl)thio)benzoic acid (100 mg, 0.42 mmol, 1.00 eq.) in methanol (5mL) was dropwise added a solution of Oxone (26 mg, 1.00 mmol, 1.00 eq.) in water (5 mL) and the reaction mixture was stirred at rt for 2.5 h. The reaction progress was monitored by TLC analysis. Upon full conversion of the starting materials, the mixture was diluted with water and extracted with DCM. The combined organic layers were washed with brine, dried ($MgSO_4$), filtered and concentrated under reduced pressure. The residue was purified by silica gel column chromatography (MeOH/DCM, 1%→2%) to afford the product (83 mg, 0.33 mmol, 78%). ^{1}H NMR (400 MHz, Methanol-*d*4) δ 8.22 (d, *J* = 8.5 Hz, 2H), 7.84 (d, *J* = 8.5 Hz, 2H), 4.16 (q, *J* = 7.1 Hz, 2H), 4.06 (d, *J* = 14.4 Hz, 1H), 3.94 (d, *J* = 14.3 Hz, 1H), 1.20 (t, *J* = 7.1 Hz, 3H). ^{13}C NMR (101 MHz, Methanol-*d*4) δ 168.37, 166.14, 148.49, 135.28, 131.64, 125.61, 63.07, 61.45, 14.33.

3-Chloro-4-(methylsulfinyl)benzoic acid (**88**)

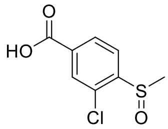

The title compound was synthesized using *tert*-butyl 3-chloro-4-(methylsulfinyl)benzoate (0.26 g, 0.94 mmol) according to procedure G. This yielded the product (0.19 g, 0.87 mmol, 92%). ^{1}H NMR (400 MHz, Methanol-*d*4) δ 8.22 (dd, *J* = 8.2, 1.6 Hz, 1H), 8.08 (d, *J* = 1.6 Hz, 1H), 7.98 (d, *J* = 8.1 Hz, 1H), 2.90 (s, 3H). ^{13}C NMR (400 MHz, Methanol-*d*4) δ 167.19, 148.93, 136.49, 132.03, 131.20, 130.45, 126.47, 41.68.

(+)-3-Chloro-4-(methylsulfinyl)benzoic acid ((+)-88)

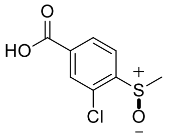

The title compound was synthesized from **(+)-135** (56 mg, 0.20 mmol) according to general procedure G. The crude product was purified by silica gel column chromatography (0-10% MeOH in DCM, 1% AcOH) to afford the title compound as a white solid (38 mg, 0.17 mmol, 85%). ^{1}H NMR (500 MHz, Methanol-*d*4) δ 8.22 (dd, *J* = 8.1, 1.5 Hz, 1H), 8.09 (d, *J* = 1.5 Hz, 1H), 7.98 (d, *J* = 8.2 Hz, 1H), 2.90 (s, 3H). ^{13}C NMR (126 MHz, Methanol-*d*4) δ 167.54, 148.74, 136.94, 132.03, 131.17, 130.43, 126.42, 41.68.

(-)-3-Chloro-4-(methylsulfinyl)benzoic acid ((-)-88)

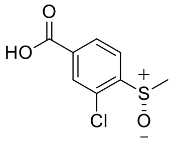

The title compound was synthesized from **(-)-135** (67 mg, 0.24 mmol) according to general procedure G. The crude product was purified by silica gel column chromatography (0-10% MeOH in DCM, 1% AcOH) to afford the title compound as a white solid (54 mg, 0.25 mmol, quant.). ^{1}H NMR (500 MHz, Methanol-*d*4) δ 8.22 (dd, *J* = 8.1, 1.6 Hz, 1H), 8.09 (d, *J* = 1.2 Hz, 1H), 7.99 (d, *J* = 7.8 Hz, 1H), 2.91 (s, 3H). ^{13}C NMR (126 MHz, Methanol-*d*4) δ 167.24, 148.93, 136.56, 132.04, 131.21, 130.45, 126.47, 41.68.

(*S*)-2-Methyl-1-(*m*-tolyl)piperazine ((*S*)-89)

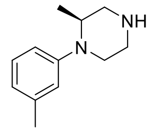

The title compound was synthesized using *tert*-butyl (*S*)-3-methyl-4-(*m*-tolyl)piperazine-1-carboxylate (150 mg, 0.52 mmol, 1.00 eq.) according to general procedure B in a yield of 90 mg (0.47 mmol, 92%). ^{1}H NMR (400 MHz, $CDCl_3$) δ 7.14 (t, *J* = 7.5 Hz, 1H), 6.78 – 6.62 (m, 3H), 3.73 (qq, *J* = 6.7, 3.7, 3.3 Hz, 1H), 3.15 – 2.78 (m, 6H), 2.31 (s, 3H), 1.03 (d, *J* = 6.5 Hz, 3H). ^{13}C NMR (101 MHz, $CDCl_3$) δ 150.78, 138.76, 128.94, 120.80, 118.44, 114.69, 51.85, 51.40, 46.46, 45.97, 21.83, 12.64.

(*R*)-2-Methyl-1-(*m*-tolyl)piperazine ((*R*)-89)

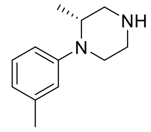

The title compound was synthesized using *tert*-butyl (*R*)-3-methyl-4-(*m*-tolyl)piperazine-1-carboxylate (0.75 g, 2.58 mmol, 1.00 eq.) according to general procedure B in a yield of 405 mg (2.13 mmol, 82%). ^{1}H NMR (400 MHz, $CDCl_3$) δ 7.20 – 7.08 (m, 1H), 6.80 – 6.68 (m, 3H), 3.72 (m, 1H), 3.67 – 3.50 (br, 1H), 3.19 – 2.82 (m, 6H), 2.31 (s, 3H), 1.03 (d, *J* = 6.5 Hz, 3H). ^{13}C NMR (101 MHz, $CDCl_3$) δ 150.56, 138.84, 128.98, 121.47, 119.16, 115.41, 51.38, 51.30, 46.09, 45.93, 21.78, 13.00.

1-(*m*-Tolyl)piperazine (**90**)

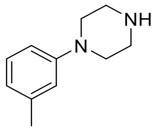

The title compound was synthesized using *tert*-butyl 4-(m-tolyl)piperazine-1-carboxylate (0.75 g, 2.70 mmol, 1 eq.) according to general procedure B in a yield of 450 mg (2.55 mmol, 95%). ^{1}H NMR (400 MHz, $CDCl_3$) δ 7.19 (dd, *J* = 8.2, 7.2 Hz, 1H), 6.85 – 6.68 (m, 3H), 5.93 (s, 1H), 3.37 – 3.27 (m, 4H), 3.26 – 3.19 (m, 4H), 2.35 (s, 3H). ^{13}C NMR (101 MHz, $CDCl_3$) δ 150.88, 138.88, 129.02, 121.48, 117.42, 113.72, 48.52, 44.66, 21.70.

1-Phenylpiperazine (**91**)

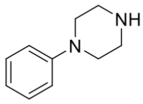

The title compound was synthesized using *tert*-butyl 4-phenylpiperazine-1-carboxylate (100 mg, 0.38 mmol, 1 eq.) according to general procedure B in a yield of 54 mg (0.34 mmol, 89%). ^{1}H NMR (400 MHz, $CDCl_3$) δ 7.31 – 7.21 (m, 2H), 6.96 – 6.88 (m, 2H), 6.90 – 6.81 (m, 1H), 3.13 (t, *J* = 5.2 Hz, 4H), 3.02 (t, *J* = 5.2 Hz, 4H), 2.37 (s, 1H). ^{13}C NMR (101 MHz, $CDCl_3$) δ 151.57, 129.20, 120.46, 116.63, 83.53.

1-(3-Fluorophenyl)piperazine (**92**)

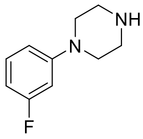

The title compound was synthesized using *tert*-butyl 4-(3-fluorophenyl)piperazine-1-carboxylate (100 mg, 0.36 mmol, 1 eq.) according to general procedure B in a yield of 62 mg (0.34 mmol, 96%). ^{1}H NMR (400 MHz, $CDCl_3$) δ 7.19 (td, *J* = 8.2, 7.0 Hz, 1H), 6.67 (ddd, *J* = 8.4, 2.4, 0.8 Hz, 1H), 6.62 – 6.46 (m, 2H),3.37 (br, 1H), 3.25 – 3.12 (m, 4H), 3.08 – 2.95 (m, 4H). ^{13}C NMR (101 MHz, $CDCl_3$) δ 163.89 (d, *J* = 243.2 Hz), 153.32 (d, *J* = 9.7 Hz), 130.19 (d, *J* = 10.0 Hz), 111.30, 106.05 (d, *J* = 21.5 Hz), 102.80 (d, *J* = 25.0 Hz), 49.54, 45.76.

1-(3-Chlorophenyl)piperazine (**93**)

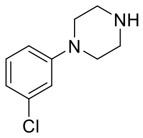

The title compound was synthesized using *tert*-butyl 4-(3-chlorophenyl)piperazine-1-carboxylate (100 mg, 0.34 mmol, 1 eq.) according to general procedure B in a yield of 62 mg (0.31 mmol, 93%). ^{1}H NMR (400 MHz, $CDCl_3$) δ 7.18 (t, *J* = 8.0 Hz, 1H), 6.94 – 6.67 (m, 3H), 6.47 (br, 1H), 3.24 (t, *J* = 5.1 Hz, 4H), 3.14 (t, *J* = 5.2 Hz, 4H). ^{13}C NMR (101 MHz, $CDCl_3$) δ 152.09, 135.03, 130.23, 120.18, 116.31, 114.43, 48.41, 44.78.

1-(4-Chlorophenyl)piperazine (**94**)

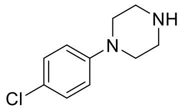

The title compound was synthesized using *tert*-butyl 4-(4-chlorophenyl)piperazine-1-carboxylate (100 mg, 0.34 mmol, 1 eq.) according to general procedure B in a yield of 66 mg (0.33 mmol, 99%). ^{1}H NMR (400 MHz, $CDCl_3$) δ 7.23 – 7.17 (m, 2H), 6.86 – 6.79

(m, 2H), 3.12 (dt, *J* = 6.1, 3.7 Hz, 4H), 3.05 (dd, *J* = 6.4, 3.3 Hz, 4H). ^{13}C NMR (101 MHz, $CDCl_3$) δ 150.32, 129.06, 124.84, 117.50, 50.10, 45.81.

1-(2-Chlorophenyl)piperazine (**95**)

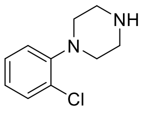

The title compound was synthesized using *tert*-butyl 4-(2-chlorophenyl)piperazine-1-carboxylate (100 mg, 0.34 mmol, 1 eq.) according to general procedure B in a yield of 63 mg (0.32 mmol, 95%). ^{1}H NMR (400 MHz, $CDCl_3$) δ 7.35 (d, *J* = 8.0 Hz, 1H), 7.22 (t, *J* = 7.7 Hz, 1H), 7.08 – 6.90 (m, 2H), 4.27 (br, 1H), 3.21 – 2.86 (m, 8H). ^{13}C NMR (101 MHz, $CDCl_3$) δ 149.21, 131.61, 130.64, 127.65, 123.94, 120.50, 51.62, 45.63.

1-(3-Bromophenyl)piperazine (**96**)

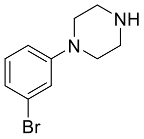

The title compound was synthesized using *tert*-butyl 4-(3-bromophenyl)piperazine-1-carboxylate (100 mg, 0.29 mmol, 1 eq.) according to general procedure B in a yield of 66 mg (0.28 mmol, 94%). ^{1}H NMR (400 MHz, $CDCl_3$) δ 7.14 – 7.05 (m, 1H), 7.02 (t, *J* = 2.1 Hz, 1H), 6.98 – 6.89 (m, 1H), 6.82 (ddd, *J* = 8.4, 2.5, 0.9 Hz, 1H), 3.12 (m, 4H), 3.03 – 2.94 (m, 4H), 1.84 (s, 1H). ^{13}C NMR (101 MHz, $CDCl_3$) δ 153.02, 130.35, 123.27, 122.23, 118.71, 114.44, 49.89, 46.02.

1-(4-Bromophenyl)piperazine (**97**)

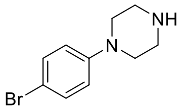

The title compound was synthesized using *tert*-butyl 4-(4-bromophenyl)piperazine-1-carboxylate (100 mg, 0.29 mmol, 1 eq.) according to general procedure B in a yield of 66 mg (0.28 mmol, 93%). ^{1}H NMR (400 MHz, $CDCl_3$) δ 7.37 – 7.31 (m, 2H), 6.82 – 6.75 (m, 2H), 3.16 – 3.09 (m, 4H), 3.07 – 2.97 (m, 4H). ^{13}C NMR (101 MHz, $CDCl_3$) δ 150.80, 131.98, 117.85, 112.07, 50.07, 45.91.

1-(3-(Trifluoromethyl)phenyl)piperazine (**98**)

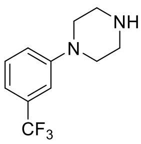

The title compound was synthesized using *tert*-butyl 4-(3-(trifluoromethyl)phenyl)piperazine-1-carboxylate (100 mg, 0.30 mmol, 1 eq.) according to general procedure B in a yield of 68 mg (0.29 mmol, 97%). ^{1}H NMR (400 MHz, $CDCl_3$) δ 7.34 (t, *J* = 7.9 Hz, 1H), 7.17 – 6.99 (m, 3H), 3.87 (s, 1H), 3.27 – 3.19 (m, 4H), 3.14 – 3.01 (m, 4H). ^{13}C NMR (101 MHz, $CDCl_3$) δ 151.62, 131.43 (q, *J* = 31.7 Hz), 129.65, 124.35 (q, *J* = 272.4 Hz), 119.03, 116.19 (q, *J* = 3.8 Hz), 112.39 (q, *J* = 3.9 Hz), 49.24, 45.50.

1-(4-(Trifluoromethyl)phenyl)piperazine (**99**)

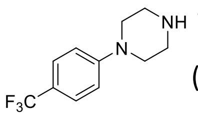

The title compound was synthesized using *tert*-butyl 4-(4-(trifluoromethyl)phenyl)piperazine-1-carboxylate (100 mg, 0.30 mmol, 1 eq.) according to general procedure B in a yield of 60 mg (0.26 mmol, 86%). ^{1}H NMR (400 MHz, $CDCl_3$) δ 7.48 (d, *J* = 8.7 Hz, 2H), 6.92 (d, *J* = 8.6 Hz, 2H), 4.26 (br, 1H), 3.36 – 3.24 (m, 4H), 3.13 – 3.04 (m, 4H). ^{13}C NMR (101 MHz, $CDCl_3$) δ 153.48, 126.47 (q, *J* = 3.7 Hz), 124.75 (q, *J* = 271.69 Hz), 120.86 (q, *J* = 32.6 Hz), 114.82, 48.49, 45.36.

1-(3-Methoxyphenyl)piperazine (**100**)

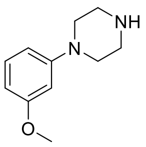

The title compound was synthesized using *tert*-butyl 4-(3-(methoxyphenyl)piperazine-1-carboxylate (200 mg, 0.68 mmol, 1 eq.) according to general procedure B in a yield of 130 mg (0.68 mmol, 99%). ^{1}H NMR (400 MHz, $CDCl_3$) δ 9.73 (br, 1H), 7.21 (t, *J* = 8.2 Hz, 1H), 6.55 – 6.49 (m, 2H), 6.45 (t, *J* = 2.3 Hz, 1H), 3.79 (s, 3H), 3.41 (m, 4H), 3.33 (m, 4H). ^{13}C NMR (101 MHz, $CDCl_3$) δ 160.81, 151.45, 130.32, 109.86, 106.53, 103.90, 55.39, 46.99, 43.47.

1-(4-Methoxyphenyl)piperazine (**101**)

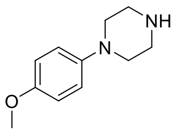

The title compound was synthesized using *tert*-butyl 4-(4-methoxyphenyl)piperazine-1-carboxylate (438 mg, 1.50 mmol, 1 eq.) according to general procedure B in a yield of 260 mg (1.35 mmol, 90%). ^{1}H NMR (400 MHz, $CDCl_3$) δ 6.89 – 6.77 (m, 4H), 3.70 (s, 3H), 2.96 (m, 8H). ^{13}C NMR (101 MHz, $CDCl_3$) δ 153.66, 146.20, 118.07, 114.30, 55.38, 51.76, 46.20.

1-(3-Nitrophenyl)piperazine (**102**)

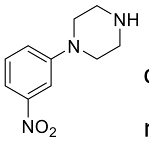

The title compound was synthesized using *tert*-butyl 4-(3-nitrophenyl)piperazine-1-carboxylate (100 mg, 0.33 mmol, 1 eq.) according to general procedure B in a yield of 66 mg (0.32 mmol, 98%). ^{1}H NMR (400 MHz, $CDCl_3$) δ 7.71 (d, *J* = 2.1 Hz, 1H), 7.65 (d, *J* = 8.0 Hz, 1H), 7.38 (t, *J* = 8.2 Hz, 1H), 7.19 (d, *J* = 8.3, 2.5 Hz, 1H), 3.40 – 3.16 (m, 4H), 3.13 – 2.95 (m, 4H). ^{13}C NMR (101 MHz, $CDCl_3$) δ 152.31, 149.32, 129.76, 121.27, 113.80, 109.74, 49.48, 45.88.

1-([1,1'-Biphenyl]-3-yl)piperazine (**103)**

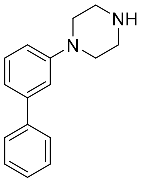

The title compound was synthesized using *tert*-butyl 4-([1,1'-biphenyl]-3-yl)piperazine-1-carboxylate (100 mg, 0.30mmol, 1 eq.) according to general procedure B. The obtained crude product was used directly without any purification.

1-(3,5-Dichlorophenyl)piperazine (**104**)

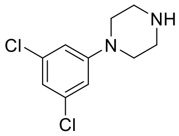

The title compound was synthesized using *tert*-butyl 4-(3,5-dichlorophenyl)piperazine-1-carboxylate (130 mg, 0.39 mmol, 1 eq.) according to general procedure B in a yield of 90 mg (0.39 mmol, 99%). ^{1}H NMR (400 MHz, $CDCl_3$) δ 6.79 (t, *J* = 1.7 Hz, 1H), 6.74 (d, *J* = 1.8 Hz, 2H), 3.17 – 3.11 (m, 4H), 3.04 – 2.96 (m, 4H), 1.42 (br, 1H). ^{13}C NMR (101 MHz, $CDCl_3$) δ 153.05, 135.47, 118.90, 113.87, 49.25, 45.74.

1-(3,4-Dichlorophenyl)piperazine (**105**)

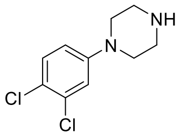

The title compound was synthesized using *tert*-butyl 4-(3,4-dichlorophenyl)piperazine-1-carboxylate (100 mg, 0.30 mmol, 1 eq.) according to general procedure B in a yield of 63 mg (0.27 mmol, 90%). ^{1}H NMR (400 MHz, $CDCl_3$) δ 7.37 (d, *J* = 2.4 Hz, 1H), 7.22 – 7.18 (m, 1H), 6.96 (d, *J* = 8.6 Hz, 1H), 3.58 (br, 1H), 3.12 (m, 4H), 3.05 (m, 4H). ^{13}C NMR (101 MHz, $CDCl_3$) δ 148.07, 130.45, 129.69, 128.64, 127.80, 121.39, 51.69, 45.71.

1-(2,4-Dichlorophenyl)piperazine (**106**)

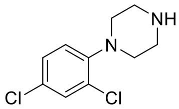

The title compound was synthesized using *tert*-butyl 4-(2,4-dichlorophenyl)piperazine-1-carboxylate (100 mg, 0.30 mmol, 1.00 eq.) according to general procedure B in a yield of 66 mg (0.28 mmol, 96%). ^{1}H NMR (400 MHz, $CDCl_3$) δ 7.35 (s, 1H), 7.18 (d, *J* = 8.6 Hz, 1H), 6.95 (d, *J* = 8.6, 2.0 Hz, 1H), 3.06 – 3.02 (m, 4H), 3.01 – 2.90 (m, 4H), 2.14 (s, 1H). ^{13}C NMR (101 MHz, $CDCl_3$) δ 148.48, 130.33, 129.55, 128.17, 127.66, 121.24, 52.56, 46.19.

1-(2,6-Dichlorophenyl)piperazine (**107**)

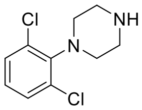

The title compound was synthesized using *tert*-butyl 4-(2,6-dichlorophenyl)piperazine-1-carboxylate (35 mg, 0.11 mmol, 1.00 eq.) according to general procedure B in a yield of 22 mg (0.10 mmol, 90%). ^{1}H NMR (400 MHz, $CDCl_3$) δ 7.26 (d, *J* = 8.1 Hz, 2H), 6.97 (t, 1H), 3.21 – 3.17 (m, 4H), 3.03 – 2.99 (m, 4H), 2.19 (s, 1H). ^{13}C NMR (101 MHz, $CDCl_3$) δ 145.41, 135.29, 129.22, 126.03, 50.81, 46.81.

(*S*)-1-(3-Fluorophenyl)-2-methylpiperazine (**108**)

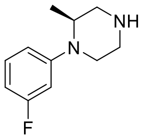

The title compound was synthesized using *tert*-butyl (*S*)-4-(3-fluorophenyl)-3-methylpiperazine-1-carboxylate (100 mg, 0.34 mmol, 1.00 eq.) according to general procedure B. The obtained crude product was used directly without any purification.

(*R*)-1-(3-Chlorophenyl)-2-methylpiperazine (**(*R*)-109**)

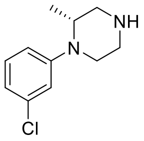

The title compound was synthesized using *tert*-butyl (*R*)-4-(3-chlorophenyl)-3-methylpiperazine-1-carboxylate (100 mg, 0.34 mmol, 1.00 eq.) according to general procedure B. The obtained crude product was used directly without any purification.

(*S*)-1-(3-Chlorophenyl)-2-methylpiperazine (**(*S*)-109**)

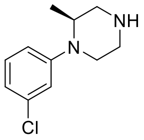

The title compound was synthesized using *tert*-butyl (*S*)-4-(3-chlorophenyl)-3-methylpiperazine-1-carboxylate (100 mg, 0.34 mmol, 1.00 eq.) according to general procedure B in a yield of 56 mg (0.26 mmol, 82%). ^{1}H NMR (400 MHz, $CDCl_3$) δ 7.16 (t, *J* = 8.1 Hz, 1H), 6.92 – 6.67 (m, 3H), 3.82 (tt, *J* = 6.7, 3.2 Hz, 1H), 3.59 (br, 1H), 3.29 – 2.78 (m, 6H), 1.08 (d, *J* = 6.6 Hz, 3H). ^{13}C NMR (101 MHz, $CDCl_3$) δ 151.54, 135.04, 130.15, 119.50, 116.88, 114.99, 50.97, 50.72, 45.84, 44.22, 12.47.

(*S*)-1-(3-Bromophenyl)-2-methylpiperazine (**(*S*)-110**)

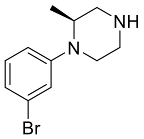

The title compound was synthesized using *tert*-butyl (S)-4-(3-bromophenyl)-3-methylpiperazine-1-carboxylate (100 mg, 0.28 mmol, 1.00 eq.) according to general procedure B in a yield of 72 mg (0.28 mmol, 98%). ^{1}H NMR (400 MHz, $CDCl_3$) δ 7.10 (t, *J* = 8.1 Hz, 1H), 7.01 (t, *J* = 2.1 Hz, 1H), 6.95 (ddd, *J* = 7.9, 1.9, 0.9 Hz, 1H), 6.81 (ddd, *J* = 8.3, 2.5, 0.9 Hz, 1H), 3.81 (m, 1H), 3.77 (br, 1H), 3.19 – 2.86 (m, 6H), 1.07 (d, *J* = 6.6 Hz, 3H). ^{13}C NMR (101 MHz, $CDCl_3$) δ 151.66, 130.41, 123.30, 122.36, 119.73, 115.42, 50.92, 50.68, 45.79, 44.17, 12.44.

(*R*)-1-(3-Bromophenyl)-2-methylpiperazine (**(*R*)-110**)

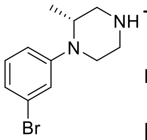

The title compound was synthesized using *tert*-Butyl (*R*)-4-(3-bromophenyl)-3-methylpiperazine-1-carboxylate (100 mg, 0.28 mmol, 1.00 eq.) according to general procedure B in a yield of 68 mg (0.27 mmol, 95%). ^{1}H NMR (400 MHz, $CDCl_3$) δ 7.11 (t, *J* = 8.1 Hz, 1H), 7.06 – 6.97 (m, 2H), 6.83 (ddd, *J* = 8.3, 2.4, 0.9 Hz, 1H), 4.17 (s, 1H), 3.82 (qt, *J* = 6.7, 3.7 Hz, 1H), 3.24 – 2.90 (m, 6H), 1.09 (d, *J* = 6.6 Hz, 3H). ^{13}C NMR (101 MHz, $CDCl_3$) δ 151.56, 130.49, 123.33, 123.00, 120.42, 116.07, 50.75, 50.61, 45.50, 44.31, 12.79.

(*R*)-2-Methyl-1-(3-(trifluoromethyl)phenyl)piperazine (**(*R*)-111**)

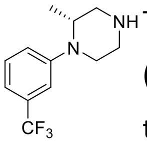

The title compound was synthesized using *tert*-butyl (*R*)-3-methyl-4-(3-(trifluoromethyl)phenyl)piperazine-1-carboxylate (100 mg, 0.29 mmol, 1.00 eq.) according to general procedure B in a yield of 70 mg (0.29 mmol, 99%). ^{1}H NMR (400 MHz, $CDCl_3$) δ 7.34 (t, *J* = 8.0 Hz, 1H), 7.13 – 7.00 (m, 3H), 3.88 (qt, *J* = 6.7, 3.4 Hz, 1H), 3.25 – 2.89 (m, 6H), 1.08 (d, *J*

= 6.6 Hz, 3H). ^{13}C NMR (101 MHz, $CDCl_3$) δ 150.64, 131.50 (q, *J* = 31.6 Hz), 129.66, 124.43 (q, *J* = 272.4 Hz), 119.68, 115.87 (q, *J* = 3.8 Hz), 113.11 (q, *J* = 3.9 Hz), 51.07, 50.70, 45.90, 44.29, 12.32.

(*S*)-2-Methyl-1-(3-(trifluoromethyl)phenyl)piperazine (**(*S*)-111)**

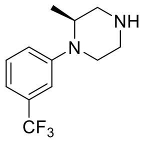

The title compound was synthesized using *tert*-butyl (*S*)-3-methyl-4-(3-(trifluoromethyl)phenyl)piperazine-1-carboxylate (100 mg, 0.29 mmol, 1.00 eq.) according to general procedure B in a yield of 62 mg (0.26 mmol, 95%). ^{1}H NMR (400 MHz, $CDCl_3$) δ 7.42 – 7.24 (m, 1H), 7.19 – 6.94 (m, 3H), 3.87 (qt, *J* = 6.6, 3.3 Hz, 1H), 3.32 – 2.80 (m, 6H), 2.50 (s, 1H), 1.08 (d, *J* = 6.6 Hz, 3H). ^{13}C NMR (101 MHz, $CDCl_3$) δ 150.63, 131.39 (q, *J* = 31.5 Hz), 129.57, 124.42 (q, *J* = 272.5 Hz), 119.28, 115.46 (q, *J* = 3.8 Hz), 112.66 (q, *J* = 3.9 Hz), 51.18, 50.60, 46.02, 44.09, 12.06.

1-(3-Chlorophenyl)-2,6-dimethylpiperazine (**112**)

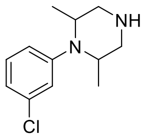

The title compound was synthesized using *tert*-butyl 4-(3-chlorophenyl)-3,5-dimethylpiperazine-1-carboxylate (70 mg, 0.22 mmol, 1 eq.) according to general procedure B in a yield of 42 mg (0.19 mmol, 86%). ^{1}H NMR (400 MHz, $CDCl_3$) δ 7.36 – 7.27 (m, 2H), 7.25 (t, *J* = 1.9 Hz, 1H), 7.16 (dt, *J* = 7.7, 1.6 Hz, 1H), 3.52 (d, *J* = 11.0 Hz, 2H), 3.44 – 3.36 (m, 2H), 2.99 (t, *J* = 11.8 Hz, 2H), 0.84 (d, *J* = 6.3 Hz, 6H).^{13}C NMR (101 MHz, $CDCl_3$) δ 134.97, 130.45, 127.74, 54.18, 49.39, 17.80.

1-(3-Chlorophenyl)-3-methylpiperazine (**113**)

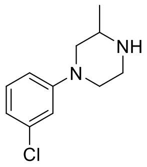

The title compound was synthesized using *tert*-butyl 4-(3-chlorophenyl)-2-methylpiperazine-1-carboxylate (104 mg, 0.34 mmol, 1 eq.) according to general procedure B in a yield of 66 mg (0.31 mmol, 93%). ^{1}H NMR (400 MHz, $CDCl_3$) δ 7.15 (t, *J* = 8.1 Hz, 1H), 6.87 (t, *J* = 2.2 Hz, 1H), 6.79 (ddt, *J* = 9.6, 7.5, 1.2 Hz, 2H), 3.50 (dq, *J* = 11.5, 1.5 Hz, 2H), 3.13 (ddd, *J* = 12.0, 3.3, 2.3 Hz, 1H), 3.07 – 2.92 (m, 2H), 2.85 – 2.69 (m, 2H), 2.41 (dd, *J* = 11.9, 10.2 Hz, 1H), 1.16 (d, *J* = 6.4 Hz, 3H). ^{13}C NMR (101 MHz, $CDCl_3$) δ 152.47, 134.96, 130.08, 119.33, 115.89, 114.07, 56.18, 50.59, 48.75, 45.60, 19.58.

(±) *cis*-1-(3-Chlorophenyl)-2,3-dimethylpiperazine (**±114**)

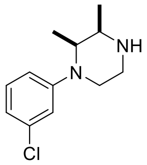

The titel product was synthetsized using 1-bromo-3-chlorobenzene (335 mg, 1.75 mmol, 1 eq.) in 2 mL 1,4-dioxane, *cis*-2,3-dimethylpiperazine (200 mg, 1.75 mmol, 1 eq.) and KHMDS (524 mg, 2.63 mmol, 1.5 eq.) at RT according to general procedure C. The residue was purified by silica gel column chromatography (MeOH/DCM, 1%→5%) to afford the product (43 mg, 0.12 mmol, 11%). ^{1}H NMR (400 MHz, $CDCl_3$) δ 7.14 (t, *J* = 8.1 Hz, 1H), 6.81 (t, *J* = 2.2

Hz, 1H), 6.74 (tdd, *J* = 8.0, 2.2, 1.0 Hz, 2H), 3.76 (m, 1H), 3.26 – 2.59 (m, 5H), 1.11 (d, *J* = 6.7 Hz, 3H), 0.97 (d, *J* = 6.7 Hz, 3H). ^{13}C NMR (101 MHz, $CDCl_3$) δ 151.40, 135.15, 130.16, 118.36, 115.43, 113.54, 54.95, 54.04, 46.07, 41.26, 19.03, 6.51.

(±) *trans*-1-(3-Chlorophenyl)-2,3-dimethylpiperazine (**±115**)

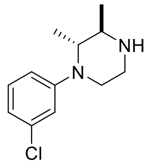

The title product was synthesized using (±) *trans*-2,3-dimethylpiperazine (0.70 g, 6.1 mmol, 1 eq.) in anhydrous dioxane (17 ml), 1-bromo-3-chlorobenzene (0.60 ml, 6.1 mmol, 1 eq.) and KHMDS solution (1M in THF, 6.1 ml, 6.1 mmol, 1 eq.) at RT according to general procedure C. The crude product was purified using column chromatography (1% -> 10% MeOH in DCM with 1% TEA) yielded the product (0.51 mg, 2.0 mmol, 32%). ^{1}H NMR (400 MHz, $CDCl_3$) δ 7.20 (t, *J* = 8.1 Hz, 1H), 6.97 (t, *J* = 2.2 Hz, 1H), 6.89 (dddd, *J* = 18.0, 8.3, 2.1, 0.9 Hz, 2H), 3.24 – 2.82 (m, 6H), 1.31 (d, *J* = 6.6 Hz, 3H), 1.05 (d, *J* = 6.4 Hz, 3H). ^{13}C NMR (101 MHz, $CDCl_3$) δ153.01, 134.81, 130.07, 121.22, 120.23, 118.30, 57.84, 54.59, 48.88, 42.59, 19.04, 14.91.

1-(3-Chlorophenyl)-2,2-dimethylpiperazine (**116**)

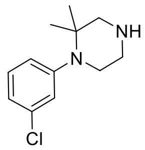

The title compound was synthesized using *tert*-butyl 4-(3-chlorophenyl)-3,3-dimethylpiperazine-1-carboxylate (86 mg, 0.27 mmol, 1 eq.) according to procedure B in a yield of 57 mg (0.25 mmol, 96%). ^{1}H NMR (400 MHz, $CDCl_3$) δ 7.18 (ddd, *J* = 8.2, 7.2, 1.2 Hz, 1H), 7.09 (dd, *J* = 7.2, 1.3 Hz, 2H), 7.02 – 6.95 (m, 1H), 3.99 (s, 1H), 3.19 – 2.96 (m, 4H), 2.81 (s, 2H), 1.07 (s, 6H). ^{13}C NMR (101 MHz, $CDCl_3$) δ 150.68, 133.70, 129.04, 127.54, 125.91, 124.71, 58.40, 54.59, 47.59, 46.72, 22.22.

1-(3-Chlorophenyl)-3,3-dimethylpiperazine (**117**)

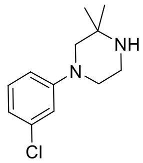

The title product was synthesized using 1-bromo-3-chlorobenzene (335 mg, 1.75 mmol, 1 eq.), 2,2-dimethylpiperazine (200 mg, 1.75 mmol, 1 eq.) in anhydrous dioxane (3 mL) and KHMDS (1M in THF, 2.1 ml, 2.1 mmol, 1.2 eq.) at RT according to general procedure C. The crude product was purified using column chromatography (MeOH/DCM, 1%-10%) yielded the product (96 mg, 0.27 mmol, 28%). ^{1}H NMR (500 MHz, $CDCl_3$) δ 7.17 – 7.10 (m, 1H), 6.84 (t, *J* = 2.2 Hz, 1H), 6.80 – 6.74 (m, 2H), 3.11 – 2.95 (m, 4H), 2.89 (s, 2H), 1.22 (s, 6H). ^{13}C NMR (126 MHz, $CDCl_3$) δ 153.20, 134.98, 130.09, 119.12, 116.04, 114.21, 60.82, 49.41, 48.19, 41.20, 26.29.

(±) 1-(4-Chloropyridin-2-yl)-trans-2,3-dimethylpiperazine (**±118**)

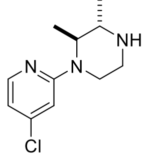

The title compound was synthesized using (±) *tert*-butyl-4-(4-chloropyridin-2-yl)-trans-2,3-dimethylpiperazine-1-carboxylate (14.5 mg, 0.045 mmol) according to procedure B. This yielded the product (7.2 mg, 0.032 mmol, 72%). ^{1}H NMR (400 MHz, $CDCl_3$) δ 8.06 (d, *J* = 5.3 Hz, 1H), 6.66 (dd, *J* = 5.4, 1.6 Hz, 1H), 6.58 (d, *J* = 1.6 Hz, 1H), 4.38 (q, *J* = 6.4 Hz, 1H), 4.27 – 4.17 (m, 1H), 3.56 (qd, *J* = 7.0, 1.7 Hz, 1H), 3.47 – 3.23 (m, 3H), 1.54 (d, *J* = 6.8 Hz, 3H), 1.45 (d, *J* = 6.8 Hz, 3H). ^{13}C NMR (101 MHz, $CDCl_3$) δ 159.21, 149.07, 145.56, 114.45, 106.82, 52.27, 51.02, 37.92, 36.48, 15.88, 14.68.

2-((4-((*R*)-3-Methyl-4-(m-tolyl)piperazine-1-carbonyl)-2-nitrophenyl)sulfinyl)acetic acid (**119**)

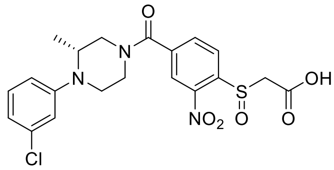

To a solution of ethyl 2-((4-((R)-4-(3-chlorophenyl)-3-methylpiperazine-1-carbonyl)-2-nitrophenyl)sulfinyl)acetate (38 mg, 0.08 mmol, 1 eq.) in MeOH (2 mL) were added TEA (2 mL) and water (1 mL). Then the reaction mixture was stirred overnight at room temperature. The reaction progress was monitored by TLC analysis. Upon full conversion of the starting materials, the pH of resulted mixture was adjusted to 1 with 1M HCl solution. Then the mixture was diluted with EtOAc and the organic layer was washed with water, dried ($MgSO_4$), filtered and concentrated under reduced pressure. The residue was purified by silica gel column chromatography (MeOH/DCM, 1%→2%) to afford the product (31 mg, 0.07 mmol, 86%). ^{1}H NMR (500 MHz, $CDCl_3$) δ 11.31 (s, 1H), 8.51 – 8.30 (m, 2H), 8.07 – 7.96 (m, 1H), 7.20 (t, *J* = 8.3 Hz, 1H), 6.84 (m, 3H), 4.59 – 3.19 (m, 10H), 1.18 – 0.95 (m, 3H). ^{13}C NMR (126 MHz, $CDCl_3$) δ 167.39, 166.68, 150.52, 145.00, 143.44, 139.44, 135.30, 133.92, 130.46, 128.16, 124.32, 120.58, 117.27, 115.26, 59.62, 52.63, 51.95, 47.52, 42.50, 12.83.

(±) 2-((2-Chloro-4-(4-(3-chlorophenyl)-trans-2,3-dimethylpiperazine-1-carbonyl)phenyl)sulfinyl)propanoic acid (**±120**)

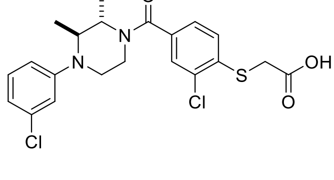

To a solution of (±) ethyl 2-((2-chloro-4-*trans*-4-(3-chlorophenyl)-2,3-dimethylpiperazine-1-carbonyl)phenyl)thio)acetate 3-chloro-4-((2-ethoxy-2-oxoethyl)thio)benzoate (36 mg, 0.75 mmol, 1 eq.) in 10 mL MeOH was added 2 M NaOH solution (0.75 mL, 1.25 mmol, 2 eq.) and the reaction mixture was sitrred for 2 h. Once completed, the solution was acidified to pH 3 using 3 M HCl solution and extraced with DCM (3x). The organic layer was dried ($MgSO_4$), filtered and concentrated under reduced pressure. The residue was purified by silica gel column chromatography (MeOH/DCM, 0-5%, with 1% AcOH). This yielded the product (0.33 g, 0.72 mmol, 96%). ^{1}H NMR (400 MHz, $CDCl_3$) δ 7.48 – 7.36 (m, 2H), 7.29 (d, *J* = 6.0 Hz, 1H), 7.17 (t, *J* = 8.3 Hz, 1H), 6.80 (d, *J* = 6.1 Hz, 2H), 6.71 (d, *J* =

8.4 Hz, 1H), 4.80 – 4.50 (m, 1H), 4.04 – 3.95 (m, 1H), 3.90 (s, 2H), 3.86 – 3.38 (m, 2H), 3.32 – 3.06 (m, 2H), 1.52 – 1.45 (m, 3H), 1.04 (dd, *J* = 55.7, 6.5 Hz, 3H). ^{13}C NMR (101 MHz, $CDCl_3$) δ 172.24, 170.27, 151.35, 137.79, 135.25, 133.79, 132.72, 130.33, 129.11, 128.18, 125.37, 119.39, 116.17, 114.18, 56.16, 49.90, 40.48, 36.75, 34.50, 17.75, 12.49.

(±) 2-((2-Chloro-4-(4-(3-chlorophenyl)-trans-2,3-dimethylpiperazine-1-carbonyl)phenyl)sulfinyl)propanoic acid (**±121**)

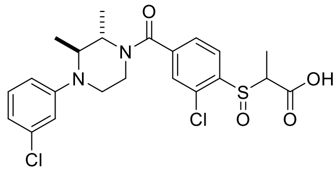

To a solution of (±) ethyl 2-((2-chloro-4-(4-(3-chlorophenyl)-*trans*-2,3-dimethylpiperazine-1-carbonyl)phenyl)sulfinyl)propanoate (0.15 g, 0.29 mmol, 1 eq.) in 4 ml MeOH was added 4 ml TEA and 2 ml water. Then, the mixture was stirred overnight. The reaction progress was monitored by TLC analysis. Once completed, the mixture was extracted with DCM and washed with water, dried ($MgSO_4$). After filtration, the filtrate was concentrated under reduced pressure. The residue was purified by silica gel column chromatography (MeOH/DCM, 0-5%). This yielded the product (0.13 g, 0.28 mmol, quant.). ^{1}H NMR (500 MHz, $CDCl_3$) δ 8.28 (s, 1H), 7.96 (dd, *J* = 23.2, 7.9 Hz, 1H), 7.58 – 7.42 (m, 2H), 7.18 (t, *J* = 8.1 Hz, 1H), 6.85 – 6.79 (m, 2H), 6.71 (d, *J* = 8.3 Hz, 1H), 4.95 – 4.58 (m, 1H), 3.96 (q, *J* = 7.1 Hz, 1H), 3.92 – 3.02 (m, 4H), 1.74 (d, *J* = 7.0 Hz, 1H), 1.54 – 1.45 (m, 3H), 1.30 (d, *J* = 7.2 Hz, 3H), 1.17 – 0.96 (m, 3H). ^{13}C NMR (126 MHz, $CDCl_3$) δ 170.41, 169.00, 151.30, 140.59, 140.22, 135.34, 131.47, 130.42, 128.78, 128.21, 126.10, 119.64, 116.36, 114.33, 60.57, 56.25, 39.89, 36.77, 17.88, 16.84, 12.56, 7.02.

(±) (3-Chloro-4-(methylsulfinyl)phenyl)(4-(3-chlorophenyl)-trans-2,3-dimethylpiperazin-1-yl)methanone (**±122**)

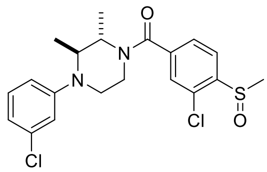

To a stirred suspension of 3-chloro-4-((2-ethoxy-2-oxoethyl)sulfinyl)benzoic acid (0.19 g, 0.87 mmol, 1 eq.) in DCM (10 ml) were added (±) *trans*-1-(3-chlorophenyl)-2,3-dimethylpiperazine (0.230 g, 1.04 mmol, 1.2 eq.), DIPEA (0.34 mg, 2.59 mmol, 3 eq.), HOBt (0.18 mg, 1.30 mmol, 1.5 eq.) and EDCl (0.25 mg, 1.30 mmol, 1.5 eq.). The mixture was stirred overnight. The reaction progress was monitored by TLC analysis. Once completed, the mixture was washed with water and brine, dried over anhydrous $MgSO_4$, filtered and concentrated. The crude product was purified using column chromatography (EtOAc / pentane, 0%-60%) to yield the product (0.27 mg, 0.64 mmol, 74%). ^{1}H NMR (400 MHz, $CDCl_3$) δ 8.04 – 7.97 (m, 2H), 7.56 – 7.38 (m, 2H), 7.14 (t, *J* = 8.2 Hz, 1H).6.60 (d, *J* = 2.4 Hz, 1H), 6.56 – 6.47 (m, 1H), 4.85 – 4.49 (m, 1H), 3.98 – 3.07 (m, 5H), 2.82 (s, 3H), 1.42 – 1.33 (m, 3H), 1.28 – 1.06 (m, 3H). ^{13}C NMR (126 MHz, $CDCl_3$) δ 168.83, 151.33, 145.25, 140.18, 135.19, 130.47, 130.33, 128.43, 127.77, 125.85, 119.34, 116.13, 114.19, 56.08, 49.57, 42.29, 41.64, 36.46, 17.76, 12.52.

(+) (3-Chloro-4-(methylsulfinyl)phenyl)(4-(3-chlorophenyl)-trans-2,3-dimethylpiperazin-1-yl)methanone (**(+)-122**)

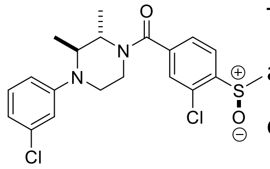

The title product was synthesized from (+)-3-chloro-4-(methylsulfinyl)benzoic acid (**(+)-88,** 25 mg, 0.11 mmol, 1 eq.) according to general procedure H. The crude product was purified using column chromatography (EtOAc / pentane, 0%-60%) to yield the product (37 mg, 87 µmol, 76%). ^{1}H NMR (400 MHz, $CDCl_3$) δ 8.04 – 7.97 (m, 2H), 7.56 – 7.38 (m, 2H), 7.14 (t, *J* = 8.2 Hz, 1H).6.60 (d, *J* = 2.4 Hz, 1H), 6.56 – 6.47 (m, 1H), 4.85 – 4.49 (m, 1H), 3.98 – 3.07 (m, 5H), 2.82 (s, 3H), 1.42 – 1.33 (m, 3H), 1.28 – 1.06 (m, 3H). ^{13}C NMR (126 MHz, $CDCl_3$) δ 168.83, 151.33, 145.25, 140.18, 135.19, 130.47, 130.33, 128.43, 127.77, 125.85, 119.34, 116.13, 114.19, 56.08, 49.57, 42.29, 41.64, 36.46, 17.76, 12.52.

(-) (3-Chloro-4-(methylsulfinyl)phenyl)(4-(3-chlorophenyl)-trans-2,3-dimethylpiperazin-1-yl)methanone (**(-)-122**)

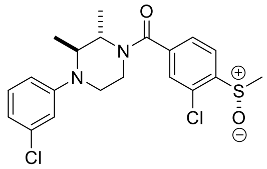

The title product was synthesized from (-)-3-chloro-4-((2-ethoxy-2-oxoethyl)sulfinyl)benzoic acid (**(-)-88**, 25 mg, 0.11 mmol, 1 eq.) according to general procedure H. The crude product was purified using column chromatography (EtOAc / pentane, 0%-60%) to yield the product (36 mg, 83 µmol, 73%). ^{1}H NMR (400 MHz, $CDCl_3$) δ 8.04 – 7.97 (m, 2H), 7.56 – 7.38 (m, 2H), 7.14 (t, *J* = 8.2 Hz, 1H) 6.60 (d, *J* = 2.4 Hz, 1H), 6.56 – 6.47 (m, 1H), 4.85 – 4.49 (m, 1H), 3.98 – 3.07 (m, 5H), 2.82 (s, 3H), 1.42 – 1.33 (m, 3H), 1.28 – 1.06 (m, 3H). ^{13}C NMR (126 MHz, $CDCl_3$) δ 168.83, 151.33, 145.25, 140.18, 135.19, 130.47, 130.33, 128.43, 127.77, 125.85, 119.34, 116.13, 114.19, 56.08, 49.57, 42.29, 41.64, 36.46, 17.76, 12.52.

(±) (3-Chloro-4-(methylsulfinyl)phenyl)(4-(4-chloropyridin-2-yl)-trans-2,3-dimethylpiperazin-1-yl)methanone (**±123**)

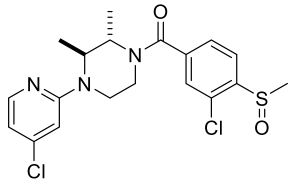

The title compound was synthesized using (±) 1-(4-chloropyridin-2-yl)-trans-2,3-dimethylpiperazine (40 mg, 0.18 mmol, 1.2 eq.) and 3-chloro-4-(methylsulfinyl)benzoic acid (32 mg, 0.15 mmol, 1 eq.) according to general procedure H. This yielded the product (56 mg, 0.13 mmol, 89%). ^{1}H NMR (500 MHz, $CDCl_3$) δ 8.40 – 7.81 (m, 2H), 7.70 – 7.33 (m, 2H), 6.94 – 6.57 (m, 2H), 5.11 – 3.06 (m, 6H), 2.89 (d, *J* = 4.4 Hz, 3H), 1.44 – 1.19 (m, 6H). ^{13}C NMR (126 MHz, $CDCl_3$) δ 169.10, 156.72, 149.65, 145.50, 144.74, 139.58, 130.74, 128.58, 126.49, 126.11, 114.61, 109.18, 53.64, 49.32, 41.53, 39.62, 36.02, 17.63, 15.48.

tert-Butyl 3,4-difluorobenzoate (124)

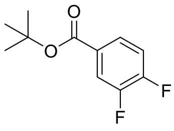

The title compound was synthesized using 3,4-difluorobenzoic acid (200 mg, 1.27 mmol, 1 eq.) according to procedure general procedure D in a yield of 198 mg (0.92 mmol, 73%). ^{1}H NMR (400 MHz, $CDCl_3$) δ 7.90 – 7.67 (m, 2H), 7.18 (q, *J* = 9.5, 8.8 Hz, 1H), 1.60 (s, 9H). ^{13}C NMR (101 MHz, $CDCl_3$) δ 163.74, 153.31 (dd, *J* = 254.9, 12.8 Hz), 150.00 (dd, *J* = 249.3, 13.0 Hz), 129.14 (dd, *J* = 5.3, 3.6 Hz), 126.31 (dd, *J* = 7.3, 3.6 Hz), 118.75 (d, *J* = 18.6 Hz), 117.06 (d, *J* = 17.8 Hz), 81.83, 28.07.

tert-Butyl 3-chloro-4-fluorobenzoate (125)

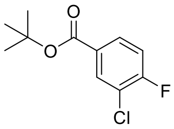

The title compound was synthesized using 4-fluoro-3-chlorobenzoic acid (1 g, 5.73 mmol, 1 eq.), Boc_2O (3.13 g, 14.3 mmol, 2.5 eq.) and DMAP (210 mg, 1.72 mmol, 0.30 eq.) according to general procedure D in a yield of 1.16 g (5.04 mmol, 88%). ^{1}H NMR (500 MHz, $CDCl_3$) δ 8.03 (dd, *J* = 7.2, 2.2 Hz, 1H), 7.88 (ddd, *J* = 8.6, 4.7, 2.2 Hz, 1H), 7.17 (t, *J* = 8.6 Hz, 1H), 1.59 (s, 9H). ^{13}C NMR (126 MHz, $CDCl_3$) δ 163.9, 160.9 (d, *J* = 255.2 Hz), 132.3, 129.9 (d, *J* = 8.4 Hz), 129.3 (d, *J* = 3.6 Hz), 121.3 (d, *J* = 18.2 Hz), 116.5 (d, *J* = 21.6 Hz), 82.1, 28.3.

tert-Butyl 3-bromo-4-fluorobenzoate (126)

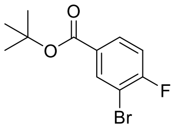

The title compound was synthesized using 3-bromo-4-fluorobenzoic acid (212 mg, 1 mmol, 1 eq.) according to general procedure D in a yield of 228 mg (0.83 mmol, 83%). ^{1}H NMR (300 MHz, $CDCl_3$) δ 8.17 (dd, *J* = 6.7, 2.0 Hz, 1H), 8.04 – 7.81 (m, 1H), 7.13 (t, *J* = 8.4 Hz, 1H), 1.58 (s, 9H). ^{13}C NMR (75 MHz, $CDCl_3$) δ 163.10, 161.38 (d, *J* = 247.8 Hz), 134.84, 130.37 (d, *J* = 8.4 Hz), 129.38 (d, *J* = 3.4 Hz), 115.93 (d, *J* = 22.8 Hz), 108.70 (d, *J* = 21.6 Hz), 81.43, 27.86.

tert-Butyl 4-((2-ethoxy-2-oxoethyl)thio)-3-fluorobenzoate (127)

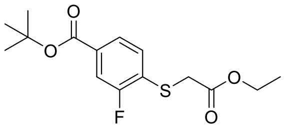

The title compound was synthesized using ethyl 2-mercaptoacetate (101 mg, 0.84 mmol, 1 eq.) and *tert*-butyl 3,4-difluorobenzoate (198 mg, 0.92 mmol, 1.1eq.) according to general procedure E in a yield of 188 mg (0.60 mmol, 71%). ^{1}H NMR (400 MHz, $CDCl_3$) δ 7.73 (dd, *J* = 8.1, 1.8 Hz, 1H), 7.64 (dd, *J* = 10.4, 1.7 Hz, 1H), 7.42 (dd, *J* = 8.2, 7.3 Hz, 1H), 4.17 (q, *J* = 7.1 Hz, 2H), 3.71 (s, 2H), 1.59 (s, 9H), 1.23 (t, *J* = 7.1 Hz, 3H). ^{13}C NMR (101 MHz, $CDCl_3$) δ 168.86, 164.20, 160.22 (d, *J* = 246.1 Hz), 132.42 (d, *J* = 7.1 Hz), 130.11 (d, *J* = 1.9 Hz), 127.99 (d, *J* = 17.4 Hz), 125.48 (d, *J* = 3.4 Hz), 116.27 (d, *J* = 23.7 Hz), 81.72, 61.83, 34.70, 28.14, 14.10.

tert-Butyl 3-chloro-4-((2-ethoxy-2-oxoethyl)thio)benzoate (**128**)

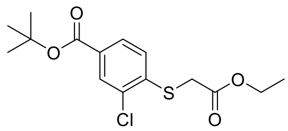

The title compound was synthesized using ethyl 2-mercaptoacetate (1.21 g, 10.1 mmol, 2 eq.) and *tert*-butyl 3-chloro-4-fluorobenzoate (1.16 g, 5.04 mmol, 1 eq.) according to general procedure E in a yield of 1.62 g (4.60 mmol, 91%). ^{1}H NMR (400 MHz, $CDCl_3$) δ 7.94 (d, *J* = 1.8 Hz, 1H), 7.83 (dd, *J* = 8.3, 1.8 Hz, 1H), 7.32 (d, *J* = 8.3 Hz, 1H), 4.20 (q, *J* = 7.1 Hz, 2H), 3.74 (s, 2H), 1.58 (s, 9H), 1.26 (t, *J* = 7.1 Hz, 3H). ^{13}C NMR (101 MHz, $CDCl_3$) δ 168.75, 164.35, 140.61, 132.17, 130.61, 130.44, 128.19, 126.62, 81.79, 62.12, 34.51, 28.25, 14.21.

tert-Butyl 3-bromo-4-((2-ethoxy-2-oxoethyl)thio)benzoate (**129**)

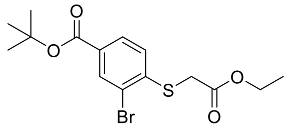

The title compound was synthesized using ethyl 2-mercaptoacetate (36 mg, 0.30 mmol, 1.1 eq.) and *tert*-butyl 3-bromo-4-fluorobenzoate (100 mg, 0.27 mmol, 1 eq.) according to procedure E in a yield of 34 mg (0.09 mmol, 34%). ^{1}H NMR (300 MHz, $CDCl_3$) δ 8.13 (d, *J* = 1.8 Hz, 1H), 7.90 (dt, *J* = 8.3, 4.3 Hz, 1H), 7.37 – 7.20 (m, 1H), 4.31 – 4.10 (m, 2H), 3.76 (s, 2H), 1.61 (s, 9H), 1.28 (t, *J* = 7.1 Hz, 3H). ^{13}C NMR (75 MHz, $CDCl_3$) δ 168.58, 164.09, 142.52, 133.63, 130.40, 128.67, 126.12, 121.48, 81.58, 62.02, 34.90, 28.14, 13.99.

tert-Butyl 3-chloro-4-((1-ethoxy-1-oxopropan-2-yl)thio)benzoate (**130**)

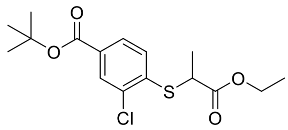

The title compound was synthesized using *tert*-butyl 3-chloro-4-fluorobenzoate (0.270 g, 1.18 mmol, 1.2 eq.) and ethyl 2-mercaptopropanoate (0.13 ml, 0.98 mmol, 1 eq.) according to procedure E. This yielded the product (0.27 g, 0.78 mmol, 79%). ^{1}H NMR (400 MHz, $CDCl_3$) δ 7.96 (s, 1H), 7.82 (d, *J* = 8.5 Hz, 1H), 7.47 (d, *J* = 8.3 Hz, 1H), 4.22 – 4.10 (m, 2H), 4.07 – 3.97 (m, 1H), 1.59 (m, 12H), 1.21 (t, *J* = 7.1 Hz, 3H). ^{13}C NMR (101 MHz, $CDCl_3$) δ 171.99, 164.21, 139.65, 133.70, 131.23, 130.49, 129.45, 127.94, 81.76, 61.69, 43.25, 28.17, 17.18, 14.11.

tert-Butyl 3-chloro-4-(methylthio)benzoate (**131**)

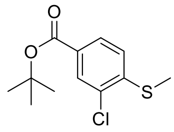

To a solution of *tert*-butyl 3-chloro-4-fluorobenzoate (0.510 g, 2.22 mmol, 1 eq.) in degassed DMF was added sodium methanethiolate (0.230 g, 3.32 mmol, 1.5 eq.) at -10 ^{o}C and the mixture was stirred overnight. The reaction progress was monitored by TLC. Once completed, the mixture was diluted with Et_2O, washed with water, dried over anhydrous $MgSO_4$ and concentrated under reduced pressure. The residue was purified using column chromatography (pentane / Et_2O, 0-10%) to yield the product (0.24 g, 0.91 mmol, 41%). ^{1}H NMR (400 MHz, $CDCl_3$) δ 7.91 (d, *J* = 1.8 Hz, 1H), 7.85 (dd, *J* = 8.3, 1.8 Hz, 1H), 7.13 (d, *J* = 8.4 Hz, 1H), 2.49 (s, 3H), 1.59 (s, 9H). ^{13}C NMR (400 MHz, $CDCl_3$) δ 164.54, 143.80, 130.83, 129.94, 129.04, 128.05, 123.94, 81.49, 28.22, 14.92.

***tert*-Butyl 4-((2-ethoxy-2-oxoethyl)sulfinyl)-3-fluorobenzoate** (**132**)

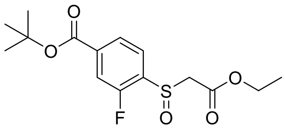

The title compound was synthesized using *tert*-butyl 4-((2-ethoxy-2-oxoethyl)thio)-3-fluorobenzoate (40 mg, 0.13 mmol, 1.00 eq.) according to general procedure F in a yield of 38 mg (0.12 mmol, 90%). ^{1}H NMR (400 MHz, $CDCl_3$) δ 8.01 (dd, *J* = 8.1, 1.4 Hz, 1H), 7.91 (dd, *J* = 8.1, 6.6 Hz, 1H), 7.74 (dd, *J* = 10.1, 1.4 Hz, 1H), 4.20 (qd, *J* = 7.2, 1.2 Hz, 2H), 3.97 (d, *J* = 13.7 Hz, 1H), 3.78 (d, *J* = 13.7 Hz, 1H), 1.61 (s, 9H), 1.25 (t, *J* = 7.1 Hz, 3H). ^{13}C NMR (101 MHz, $CDCl_3$) δ 164.23, 163.55, 157.35 (d, *J* = 247.8 Hz), 137.41 (d, *J* = 6.9 Hz), 134.60 (d, *J* = 17.2 Hz), 126.37, 126.32 (d, *J* = 4.1 Hz), 116.73 (d, *J* = 22.0 Hz), 82.65, 62.44, 58.64, 28.18, 14.17.

***tert*-Butyl 3-chloro-4-((2-ethoxy-2-oxoethyl)sulfinyl)benzoate** (**133**)

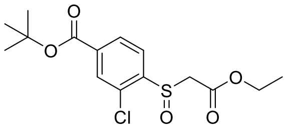

The title compound was synthesized using *tert*-butyl 3-chloro-4-((2-ethoxy-2-oxoethyl)thio)benzoate (140 mg, 0.42 mmol, 1.00 eq.) according to general procedure F in a yield of 147 mg (0.42 mmol, quant.). ^{1}H NMR (400 MHz, $CDCl_3$) δ 8.12 (d, *J* = 8.1 Hz, 1H), 8.06 – 7.92 (m, 2H), 4.29 – 4.18 (m, 2H), 4.03 (d, *J* = 13.1 Hz, 1H), 3.69 (d, *J* = 13.8 Hz, 1H), 1.61 (s, 9H), 1.26 (t, *J* = 7.0 Hz, 3H). ^{13}C NMR (126 MHz, $CDCl_3$) δ = 164.5, 163.6, 145.2, 136.5, 130.8, 130.1, 128.8, 126.5, 82.7, 62.4, 58.1, 28.2, 14.2.

***tert*-Butyl 3-bromo-4-((2-ethoxy-2-oxoethyl)sulfinyl)benzoate** (**134**)

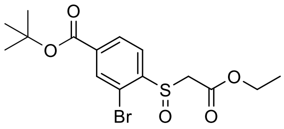

The title compound was synthesized *tert*-butyl 3-bromo-4-((2-ethoxy-2-oxoethyl)thio)benzoate (110 mg, 0.29 mmol, 1.00 eq.) according to general procedure F in a yield of 92 mg (0.23 mmol, 81%). ^{1}H NMR (300 MHz, $CDCl_3$) δ 8.16 (dd, *J* = 7.4, 1.2 Hz, 2H), 7.98 (d, *J* = 8.6 Hz, 1H), 4.22 (m, 2H), 4.07 (d, *J* = 13.8 Hz, 1H), 3.69 (d, *J* = 13.8 Hz, 1H), 1.61 (s, 9H), 1.27 (t, *J* = 7.1 Hz, 3H). ^{13}C NMR (75 MHz, $CDCl_3$) δ 164.45, 163.31, 146.81, 136.39, 133.80, 129.24, 126.75, 118.32, 82.58, 62.32, 58.24, 28.07, 14.11.

***tert*-Butyl 3-chloro-4-((1-ethoxy-1-oxopropan-2-yl)sulfinyl)benzoate** (**135**)

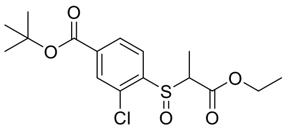

The title compound was synthesized using *tert*-butyl 3-chloro-4-((1-ethoxy-1-oxopropan-2-yl) thiol)benzoate (0.13 g, 0.38 mmol, 1 eq.) according to procedure F. This yielded the product (0.13 g, 0.35 mmol, 92%). ^{1}H NMR (400 MHz, $CDCl_3$) δ 8.11 (dd, *J* = 8.2, 1.5 Hz, 1H), 8.00 (d, *J* = 1.6 Hz, 1H), 7.90 (d, *J* = 8.1 Hz, 1H), 4.31 (q, *J* = 8 Hz, 2H), 3.91 (q, *J* = 8 Hz, 1H), 1.61 (s, 9H), 1.35 (t, *J* = 7.1 Hz, 3H), 1.25 (d, *J* = 7.2 Hz, 3H). ^{13}C NMR (101 MHz, $CDCl_3$) δ 168.58, 163.61, 144.19, 136.33, 130.88, 130.32, 128.49, 127.58, 82.73, 77.48, 77.16, 76.85, 62.57, 60.49, 28.21, 14.26, 6.62.

***tert*-Butyl 3-chloro-4-(methylsulfinyl)benzoate** (**136**)

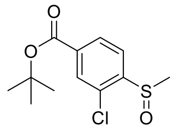

The title compound was synthesized using *tert*-butyl 3-chloro-4-(methylthio)benzoate (0.24 g, 0.91 mmol, 1 eq.) according to procedure F. This yielded the product (0.290 g, 1.17 mmol, quant.). ^{1}H NMR (500 MHz, $CDCl_3$) δ 8.13 (dd, *J* = 8.0, 1.6 Hz, 1H), 8.03 – 7.98 (m, 2H), 2.86 (s, 3H), 1.62 (s, 9H). ^{13}C NMR (125 MHz, $CDCl_3$) δ 163.34, 147.97, 135.70, 130.48, 129.52, 128.73, 125.20, 82.24, 41.37, 27.94.

(+)-*tert*-Butyl 3-chloro-4-(methylsulfinyl)benzoate ((+)-136)

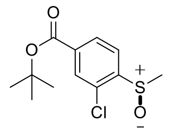

A solution of **(*S*)-177** (2.0 mg, 4.0 µmol, 1 mol%) and $Mn(OTf)_2$ (1.4 mg, 4.2 µmol, 1 mol%) in DCM (4.2 mL) was stirred for 3 h. The solution was cooled to 0 °C before *tert*-butyl 3-chloro-4-(methylthio)benzoate (109 mg, 0.42 mmol, 1 eq.) was added. After 5 minutes, acetic acid (126 mg, 2.10 mmol, 5 eq.) was added and another 5 minutes later H_2O_2 (36% aq., 0.70 mL, 0.84 mmol, 2 eq.). The contents were stirred at 0 °C for 1 h. The organic layer was separated, dried ($MgSO_4$), filtered and evaporated in vacuo. The crude was then purified by column chromatography (0-25% EtOAc in pentane) to afford the title product as a yellow oil (66 mg, 0.24 mmol, 57%). $[\alpha]^{20}_{D}$ = +127.8; *ee*= 70% (as determined by chiral HPLC using 2:98 iPrOH/heptane, Chiralcell OD). ^{1}H NMR (500 MHz, $CDCl_3$) δ 8.08 (dd, *J* = 8.1, 1.5 Hz, 1H), 7.97 (d, *J* = 8.2 Hz, 1H), 7.95 (d, *J* = 1.5 Hz, 1H), 2.80 (s, 3H), 1.57 (s, 9H). ^{13}C NMR (126 MHz, $CDCl_3$) δ 163.61, 148.10, 135.91, 130.70, 129.73, 128.93, 125.40, 82.50, 41.55, 28.14.

(-)-*tert*-Butyl 3-chloro-4-(methylsulfinyl)benzoate ((-)-136)

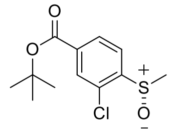

A solution of **(*R*)-177** (2.0 mg, 4.0 µmol, 1 mol%) and $Mn(Otf)_2$ (1.4 mg, 4.2 µmol, 1 mol%) in DCM (4.2 mL) was stirred for 3 h. The solution was cooled to 0 °C before *tert*-butyl 3-chloro-4-(methylthio)benzoate (109 mg, 0.422 mmol, 1 eq.) was added. After 5 minutes, acetic acid (126 mg, 2.10 mmol, 5 eq.) was added and another 5 minutes later H_2O_2 (36% aq., 0.70 mL, 0.84 mmol, 2 eq.). The contents were stirred at 0 °C for 1 h. The organic layer was separated, dried ($MgSO_4$), filtered and evaporated in vacuo. The crude was then purified by column chromatography (0-25% EtOAc in pentane) to afford the title as a yellow oil (93 mg, 0.338 mmol, 81%). $[\alpha]^{20}_{D}$ = -150.0°. *ee*= 81% (as determined by chiral HPLC using 2:98 iPrOH/heptane, Chiralcell OD). ^{1}H NMR (400 MHz, $CDCl_3$) δ 8.07 (dd, *J* = 8.1, 1.5 Hz, 1H), 7.97 (d, *J* = 8.2 Hz, 1H), 7.95 (d, *J* = 1.5 Hz, 1H), 2.80 (s, 3H), 1.56 (s, 9H). ^{13}C NMR (101 MHz, $CDCl_3$) δ 163.59, 148.09, 135.90, 130.69, 129.72, 128.92, 125.38, 82.49, 41.54, 28.13.

tert-Butyl (S)-3-methyl-4-(m-tolyl)piperazine-1-carboxylate ((S)-137)

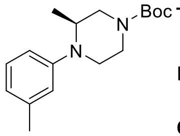

The title compound was synthesized using 1-bromo-3-methylbenzene (100 mg, 0.59 mmol, 1 eq.), *tert*-butyl (*S*)-3-methylpiperazine-1-carboxylate (176 mg, 0.88 mmol, 1.5 eq.), Cs_2CO_3 (286 mg, 0.88 mmol, 1.5 eq.), rac-BINAP (23.25 mg, 0.04 mmol, 0.06 eq.) and palladium diacetate (5.25 mg, 0.02 mmol, 0.04 eq.) according to general procedure A in a yield of 130 mg (0.45 mmol, 77%). ^{1}H NMR (400 MHz, $CDCl_3$) δ 7.14 (t, *J* = 7.7 Hz, 1H), 6.71 – 6.67 (m, 3H), 4.04 – 3.66 (m, 3H), 3.44 – 2.99 (m, 4H), 2.30 (s, 3H), 1.48 (s, 9H), 0.98 (d, *J* = 6.5 Hz, 3H). ^{13}C NMR (101 MHz, $CDCl_3$) δ 155.13, 150.22, 138.86, 129.07, 120.93, 118.05, 114.33, 79.70, 51.65, 49.45, 48.25, 44.30, 28.50, 21.85, 12.25.

tert-Butyl (R)-3-methyl-4-(m-tolyl)piperazine-1-carboxylate ((R)-137)

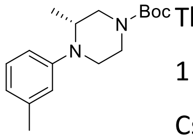

The title compound was synthesized using 1-bromo-3-methylbenzene (1 g, 5.85 mmol, 1 eq.), *tert*-butyl (*R*)-3-methylpiperazine-1-carboxylate (1.17 g, 5.85 mmol, 1 eq.), Cs_2CO_3 (2.86 g, 8.77 mmol, 1.5 eq.), rac-BINAP (233 mg, 0.35 mmol, 0.06 eq.) and palladium diacetate (52.53 mg, 0.23 mmol, 0.04 eq.) according to general procedure A in a yield of 1.05 g (3.62 mmol, 62%). The crude product was used in the next step without further purification.

tert-Butyl 4-(m-tolyl)piperazine-1-carboxylate (138)

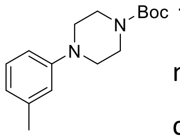

The title compound was synthesized using 1-bromo-3-methylbenzene (0.50 g, 2.92 mmol, 1 eq.), *tert*-butyl piperazine-1-carboxylate (0.82 g, 4.39 mmol, 1.5 eq.), cesium carbonate (1.43 g, 4.39 mmol, 1.5 eq.), rac-BINAP (116 mg, 0.18 mmol, 0.06 eq.) and palladium diacetate (26.3 mg, 0.12 mmol, 0.04 eq.) according to general procedure A in a yield of 0.75 g (2.70 mmol, 92%). ^{1}H NMR (400 MHz, $CDCl_3$) δ 7.13 (t, *J* = 7.8 Hz, 1H), 6.75 – 6.64 (m, 3H), 3.54 (t, *J* = 5.2 Hz, 4H), 3.06 (t, *J* = 5.2 Hz, 4H), 2.30 (s, 3H), 1.48 (s, 9H). ^{13}C NMR (101 MHz, $CDCl_3$) δ 154.51, 151.22, 138.59, 128.86, 121.01, 117.33, 113.63, 79.57, 49.34, 28.32, 21.63.

tert-Butyl 4-phenylpiperazine-1-carboxylate (139)

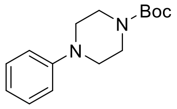

The title compound was synthesized using bromobenzene (160 mg, 1.00 mmol, 1 eq.), *tert*-butyl piperazine-1-carboxylate (190 mg, 1.00 mmol, 1 eq.), sodium *tert*-butoxide (240 mg, 2.50 mmol, 2.5 eq.), *rac*-BINAP (60 mg, 0.10 mmol, 0.10 eq.) and palladium diacetate (3 mg, 0.015 mmol, 0.015 eq.) according to general procedure A in a yield of 252 mg (0.10 mmol, 97%). ^{1}H NMR (400 MHz, $CDCl_3$) δ 7.31 – 7.25 (m, 2H), 6.97 – 6.91 (m, 2H), 6.91 – 6.86 (m, 1H), 3.58 (t, *J* = 5.2 Hz, 4H), 3.13 (t, *J* = 5.2 Hz, 4H), 1.48 (s, 9H). ^{13}C NMR (101 MHz, $CDCl_3$) δ 154.82, 151.37, 129.28, 120.37, 116.72, 79.95, 49.51, 28.53.

tert-Butyl 4-(3-fluorophenyl)piperazine-1-carboxylate (**140**)

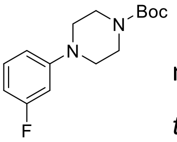

The title compound was synthesized using 1-bromo-3-fluorobenzene (100 mg, 0.57 mmol, 1 eq.), *tert*-butyl piperazine-1-carboxylate (128 mg, 0.69 mmol, 1.2 eq.), sodium *tert*-butoxide (82 mg, 0.86 mmol, 1.5 eq.), rac-BINAP (21 mg, 0.034 mmol, 0.06 eq.) and palladium diacetate (5 mg, 0.023 mmol, 0.04 eq.) according to general procedure A in a yield of 145 mg (0.52 mmol, 91%). The crude product was used in the next step without further purification.

tert-Butyl 4-(3-chlorophenyl)piperazine-1-carboxylate (**141**)

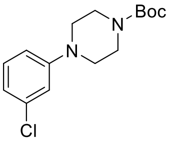

The title compound was synthesized using 1-bromo-3-chlorobenzene (191 mg, 1.00 mmol, 1 eq.), *tert*-butyl piperazine-1-carboxylate (186 mg, 1.00 mmol, 1.00 eq.), sodium *tert*-butoxide (144 mg, 1.50 mmol, 1.5 eq.), rac-BINAP (37.4 mg, 0.06 mmol, 0.06 eq.) and palladium diacetate (9 mg, 0.04 mmol, 0.04 eq.) according to general procedure A in a yield of 190 mg (0.64 mmol, 64 %). ^{1}H NMR (400 MHz, $CDCl_3$) δ 7.16 (t, *J* = 8.1 Hz, 1H), 6.87 (t, *J* = 2.2 Hz, 1H), 6.82 (ddd, *J* = 7.9, 2.0, 0.9 Hz, 1H), 6.77 (ddd, *J* = 8.4, 2.4, 0.9 Hz, 1H), 3.97 – 3.35 (m, 4H), 3.12 (t, *J* = 5.2 Hz, 4H), 1.48 (s, 9H). ^{13}C NMR (101 MHz, $CDCl_3$) δ 154.67, 152.31, 134.99, 130.15, 119.83, 116.30, 114.44, 80.01, 48.89, 28.45.

tert-Butyl 4-(4-chlorophenyl)piperazine-1-carboxylate (**142**)

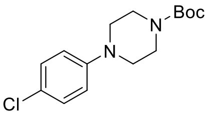

The title compound was synthesized using 1-bromo-4-chlorobenzene (191 mg, 1.00 mmol, 1 eq.), *tert*-butyl piperazine-1-carboxylate (186 mg, 1 mmol, 1.00 eq.), sodium *tert*-butoxide (144 mg, 1.50 mmol, 1.5 eq.), rac-BINAP (37.4 mg, 0.06 mmol, 0.06 eq.) and palladium diacetate (9 mg, 0.04 mmol, 0.04 eq.) according to general procedure A in a yield of 264 mg (0.89 mmol, 89 %). ^{1}H NMR (400 MHz, $CDCl_3$) δ 7.24 – 7.16 (m, 2H), 6.86 – 6.76 (m, 2H), 3.62 – 3.49 (m, 4H), 3.08 (t, *J* = 5.2 Hz, 4H), 1.48 (s, 9H). ^{13}C NMR (101 MHz, $CDCl_3$) δ 154.73, 149.97, 129.11, 125.18, 117.89, 80.05, 49.48, 28.50.

tert-Butyl 4-(2-chlorophenyl)piperazine-1-carboxylate (**143**)

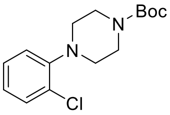

The title compound was synthesized using 1-bromo-2-chlorobenzene (191 mg, 1.00 mmol, 1 eq.), *tert*-butyl piperazine-1-carboxylate (186 mg, 1 mmol, 1.00 eq.), sodium *tert*-butoxide (144 mg, 1.50 mmol, 1.5 eq.), rac-BINAP (37.4 mg, 0.06 mmol, 0.06 eq.) and palladium diacetate (9 mg, 0.04 mmol, 0.04 eq.) according to general procedure A in a yield of 241 mg (0.81 mmol, 81%). ^{1}H NMR (400 MHz, $CDCl_3$) δ 7.35 (dt, *J* = 7.9, 1.6 Hz, 1H), 7.28 – 7.11 (m, 1H), 7.05 – 6.89 (m, 2H), 3.68 – 3.49 (m, 4H), 3.09 – 2.83 (m, 4H), 1.49 (s, 9H). ^{13}C NMR (101 MHz, $CDCl_3$) δ 154.74, 149.05, 130.62, 128.87, 127.58, 123.95, 120.45, 79.66, 51.18, 44.08, 28.42.

***tert*-Butyl 4-(3-bromophenyl)piperazine-1-carboxylate** (**144**)

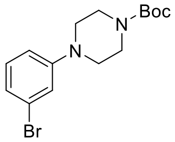

The title compound was synthesized using 1,3-dibromobenzene (236 mg, 1.00 mmol, 1 eq.), *tert*-butyl piperazine-1-carboxylate (186 mg, 1 mmol, 1.00 eq.), sodium *tert*-butoxide (144 mg, 1.50 mmol, 1.5 eq.), rac-BINAP (37.4 mg, 0.06 mmol, 0.06 eq.) and palladium diacetate (9 mg, 0.04 mmol, 0.04 eq.) according to general procedure A in a yield of 259 mg (0.76 mmol, 76%). ^{1}H NMR (400 MHz, $CDCl_3$) δ 7.10 (t, *J* = 8.1 Hz, 1H), 7.02 (t, *J* = 2.1 Hz, 1H), 6.97 (ddd, *J* = 7.9, 1.8, 0.9 Hz, 1H), 6.81 (ddd, *J* = 8.3, 2.5, 0.9 Hz, 1H), 3.62 – 3.46 (m, 4H), 3.11 (t, *J* = 5.1 Hz, 4H), 1.48 (s, 9H). ^{13}C NMR (101 MHz, $CDCl_3$) δ 154.66, 152.45, 130.44, 123.26, 122.79, 119.24, 114.94, 80.02, 48.91, 43.69, 28.46.

***tert*-Butyl 4-(4-bromophenyl)piperazine-1-carboxylate** (**145**)

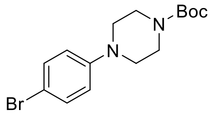

The title compound was synthesized using 1,4-dibromobenzene (236 mg, 1.00 mmol, 1 eq.), *tert*-butyl piperazine-1-carboxylate (186 mg, 1 mmol, 1.00 eq.), sodium *tert*-butoxide (144 mg, 1.50 mmol, 1.5 eq.), rac-BINAP (37.4 mg, 0.06 mmol, 0.06 eq.) and palladium diacetate (9 mg, 0.04 mmol, 0.04 eq.) according to general procedure A in a yield of 256 mg (0.75 mmol, 75%). ^{1}H NMR (400 MHz, $CDCl_3$) δ 7.38 – 7.31 (m, 2H), 6.82 – 6.73 (m, 2H), 3.68 – 3.41 (m, 4H), 3.09 (t, *J* = 5.2 Hz, 4H), 1.48 (s, 9H). ^{13}C NMR (101 MHz, $CDCl_3$) δ 154.71, 150.33, 132.02, 118.25, 112.49, 80.06, 49.29, 43.74, 28.50.

***tert*-Butyl 4-(3-(trifluoromethyl)phenyl)piperazine-1-carboxylate** (**146**)

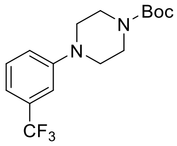

The title compound was synthesized using 1-bromo-3-(trifluoromethyl)benzene (225 mg, 1.00 mmol, 1 eq.), *tert*-butyl piperazine-1-carboxylate (186 mg, 1 mmol, 1.00 eq.), sodium *tert*-butoxide (144 mg, 1.50 mmol, 1.5 eq.), rac-BINAP (37.4 mg, 0.06 mmol, 0.06 eq.) and palladium diacetate (9 mg, 0.04 mmol, 0.04 eq.) according to general procedure A in a yield of 289 mg (0.88 mmol, 88%). ^{1}H NMR (400 MHz, $CDCl_3$) δ 8.36 (ddt, *J* = 8.2, 7.2, 0.9 Hz, 1H), 8.14 – 8.05 (m, 3H), 4.65 – 4.55 (m, 4H), 4.19 (t, *J* = 5.2 Hz, 4H), 2.49 (s, 9H). ^{13}C NMR (101 MHz, $CDCl_3$) δ 154.78, 151.41, 131.65 (q, *J* = 31.7 Hz), 129.79, 124.35 (q, *J* = 272.70 Hz), 119.51, 116.65 (q, *J* = 3.6 Hz), 112.89 (q, *J* = 3.8 Hz), 80.22, 49.10, 28.54..

***tert*-Butyl 4-(4-(trifluoromethyl)phenyl)piperazine-1-carboxylate** (**147**)

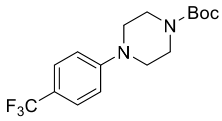

The title compound was synthesized using 1-bromo-4-(trifluoromethyl)benzene (225 mg, 1.00 mmol, 1 eq.), *tert*-butyl piperazine-1-carboxylate (186 mg, 1 mmol, 1.00 eq.), sodium *tert*-butoxide (144 mg, 1.50 mmol, 1.5 eq.), rac-BINAP (37.4 mg, 0.06 mmol, 0.06 eq.) and palladium diacetate (9 mg, 0.04 mmol, 0.04 eq.) according to general

procedure A in a yield of 218 mg (0.66 mmol, 66%). ^{1}H NMR (400 MHz, $CDCl_3$) δ 7.55 – 7.44 (m, 2H), 6.95 (d, *J* = 8.7 Hz, 2H), 3.65 – 3.53 (m, 4H), 3.25 (t, *J* = 5.2 Hz, 4H). 1.49 (s, 9 H).

***tert*-Butyl 4-(3-methoxyphenyl)piperazine-1-carboxylate** (**148**)

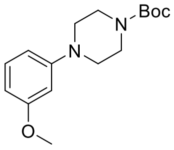

The title compound was synthesized using 1-bromo-3-methoxybenzene (374 mg, 2.00 mmol, 1 eq.), *tert*-butyl piperazine-1-carboxylate (373 mg, 2 mmol, 1.00 eq.), sodium *tert*-butoxide (288 mg, 3.00 mmol, 1.5 eq.), rac-BINAP (75 mg, 0.12 mmol, 0.06 eq.) and palladium diacetate (18 mg, 0.08 mmol, 0.04 eq.) according to general procedure A in a yield of 433 mg (1.48 mmol, 74%). ^{1}H NMR (400 MHz, $CDCl_3$) δ 7.16 (t, *J* = 8.1 Hz, 1H), 6.52 (dd, *J* = 8.3, 2.5 Hz, 1H), 6.47 – 6.41 (m, 2H), 3.77 (s, 3H), 3.56 (t, *J* = 5.0 Hz, 4H), 3.21 – 2.99 (m, 4H), 1.48 (s, 9H). ^{13}C NMR (101 MHz, $CDCl_3$) δ 160.57, 154.63, 152.61, 129.81, 109.25, 104.93, 102.95, 79.77, 55.10, 49.25, 28.41.

***tert*-Butyl 4-(4-methoxyphenyl)piperazine-1-carboxylate** (**149**)

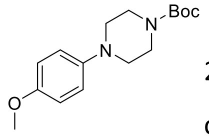

The title compound was synthesized using 1-bromo-4-methoxybenzene (500 mg, 2.67 mmol, 1 eq.), *tert*-butyl piperazine-1-carboxylate (597 mg, 3.21 mmol, 1.2 eq.), cesium carbonate (1307 mg, 4.01 mmol, 1.5 eq.), rac-BINAP (106 mg, 0.16 mmol, 0.06 eq.) and palladium diacetate (24 mg, 0.11 mmol, 0.04 eq.) according to general procedure A in a yield of 438 mg (1.50 mmol, 56%). ^{1}H NMR (400 MHz, $CDCl_3$) δ 6.91 – 6.72 (m, 4H), 3.70 (s, 3H), 3.60 – 3.46 (m, 4H), 3.01 – 2.87 (m, 4H), 1.45 (s, 9H). ^{13}C NMR (101 MHz, $CDCl_3$) δ 154.52, 154.07, 145.48, 118.68, 114.30, 79.57, 55.31, 50.76, 28.32.

***tert*-Butyl 4-(3-nitrophenyl)piperazine-1-carboxylate** (**150**)

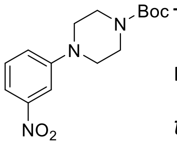

The title compound was synthesized using 1-bromo-3-nitrobenzene (202 mg, 1.00 mmol, 1 eq.), *tert*-butyl piperazine-1-carboxylate (186 mg, 1 mmol, 1.00 eq.), sodium *tert*-butoxide (144 mg, 1.50 mmol, 1.5 eq.), rac-BINAP (37 mg, 0.06 mmol, 0.06 eq.) and palladium diacetate (9 mg, 0.04 mmol, 0.04 eq.) according to general procedure A in a yield of 150 mg (0.49 mmol, 49%). ^{1}H NMR (400 MHz, $CDCl_3$) δ 7.80 – 7.55 (m, 2H), 7.39 (t, *J* = 8.2 Hz, 1H), 7.20 (dd, *J* = 8.4, 2.5 Hz, 1H), 4.03 – 3.44 (t, *J* = 5.2 Hz, 4H), 3.25 (t, *J* = 5.2 Hz, 4H), 1.50 (s, 9H). ^{13}C NMR (101 MHz, $CDCl_3$) δ 154.57, 151.71, 149.17, 129.79, 121.61, 114.61, 110.02, 80.13, 48.44, 28.38.

***tert*-Butyl 4-([1,1'-biphenyl]-3-yl)piperazine-1-carboxylate** (**151**)

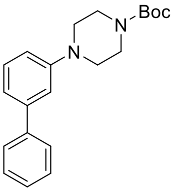

To a mixture of phenylboronic acid (107 mg, 0.88 mmol, 1.5 eq.) and *tert*-butyl 4-(3-bromophenyl)piperazine-1-carboxylate (200 mg, 0.59 mmol, 1 eq.) in 1.4-dioxane (5 mL) was added cesium carbonate (573 mg, 1.76 mmol, 3 eq.) and tetrakis(triphenylphosphine)palladium (14 mg, 0.012 mmol, 0.02 eq.). Then the reaction mixture was degassed with N_2 for 30 min. The mixture was heated to 80 ^oC for 5h. The reaction progress was monitored by TLC analysis. Upon full conversion of the starting materials, the mixture was diluted with DCM and washed with water, dried ($MgSO_4$), filtered and concentrated under reduced pressure. The residue was purified by silica gel column chromatography (Et_2O / pentane, 0 – 10 %) to yielded the product (181 mg, 0.53 mmol, 91%). 1H NMR (400 MHz, $CDCl_3$) δ 7.55 (dt, *J* = 6.3, 1.2 Hz, 2H), 7.39 (dd, *J* = 8.4, 6.8 Hz, 2H), 7.30 (td, *J* = 7.6, 2.6 Hz, 2H), 7.14 – 7.05 (m, 2H), 6.88 (dd, *J* = 8.1, 2.5 Hz, 1H), 3.57 (t, *J* = 5.2 Hz, 4H), 3.15 (t, *J* = 5.1 Hz, 4H), 1.48 (s, 9H). 13C NMR (101 MHz, $CDCl_3$) δ 154.66, 151.64, 142.31, 141.56, 129.51, 128.67, 127.26, 127.19, 119.31, 115.59, 115.54, 79.81, 49.44, 28.42.

***tert*-Butyl 4-(3,5-dichlorophenyl)piperazine-1-carboxylate** (**152**)

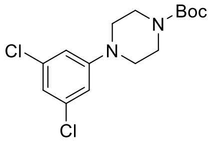

The title compound was synthesized using 1-bromo-3,5-dichlorobenzene (226 mg, 1.00 mmol, 1 eq.), *tert*-butyl piperazine-1-carboxylate (186 mg, 1.00 mmol, 1 eq.), sodium *tert*-butoxide (144 mg, 1.50 mmol, 1.5 eq.), rac-BINAP (37.4 mg, 0.06 mmol, 0.06 eq.) and palladium diacetate (8.96 mg, 0.04 mmol, 0.04 eq.) according to general procedure A in a yield of 192 mg (0.58 mmol, 58%). ^1H NMR (400 MHz, $CDCl_3$) δ 6.82 (t, *J* = 1.7 Hz, 1H), 6.74 (d, *J* = 1.7 Hz, 2H), 3.62 – 3.48 (m, 4H), 3.15 (t, *J* = 5.2 Hz, 4H), 1.48 (s, 9H). ^{13}C NMR (101 MHz, $CDCl_3$) δ 154.71, 152.65, 135.61, 119.44, 114.35, 80.28, 48.48, 28.52.

***tert*-Butyl 4-(3,4-dichlorophenyl)piperazine-1-carboxylate** (**153**)

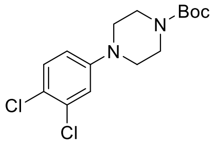

The title compound was synthesized using 4-bromo-1,2-dichlorobenzene (226 mg, 1.00 mmol, 1 eq.), *tert*-butyl piperazine-1-carboxylate (186 mg, 1.00 mmol, 1 eq.), sodium *tert*-butoxide (144 mg, 1.50 mmol, 1.5 eq.), rac-BINAP (37.4 mg, 0.06 mmol, 0.06 eq.) and palladium diacetate (8.96 mg, 0.04 mmol, 0.04 eq.) according to general procedure A in a yield of 209 mg (0.63 mmol, 63%). ^1H NMR (400 MHz, $CDCl_3$) δ 7.37 (d, *J* = 2.4 Hz, 1H), 7.19 (dd, *J* = 8.6, 2.4 Hz, 1H), 6.93 (d, *J* = 8.6 Hz, 1H), 3.65 – 3.54 (m, 4H), 2.95 (t, *J* = 4.9 Hz, 4H), 1.49 (s, 9H). ^{13}C NMR (101 MHz, $CDCl_3$) δ 154.86, 147.93, 130.45, 129.71, 128.67, 127.76, 121.35, 79.97, 51.29, 28.52.

***tert*-Butyl 4-(2,4-dichlorophenyl)piperazine-1-carboxylate (154)**

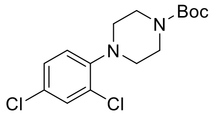

The title compound was synthesized using 1-bromo-2,4-dichlorobenzene (226 mg, 1.00 mmol, 1 eq.), *tert*-butyl piperazine-1-carboxylate (186 mg, 1.00 mmol, 1 eq.), sodium *tert*-butoxide (144 mg, 1.50 mmol, 1.5 eq.), rac-BINAP (37.4 mg, 0.06 mmol, 0.06 eq.) and palladium diacetate (8.96 mg, 0.04 mmol, 0.04 eq.) according to general procedure A in a yield of 200 mg (0.60 mmol, 60%). ^{1}H NMR (400 MHz, $CDCl_3$) δ 7.37 (d, *J* = 2.5 Hz, 1H), 7.19 (dd, *J* = 8.6, 2.4 Hz, 1H), 6.93 (d, *J* = 8.6 Hz, 1H), 3.59 (t, 4H), 2.95 (t, 4H), 1.49 (s, 9H). ^{13}C NMR (101 MHz, $CDCl_3$) δ 154.87, 147.96, 130.46, 129.72, 128.66, 127.76, 121.35, 79.97, 51.30, 28.53.

***tert*-Butyl 4-(2,6-dichlorophenyl)piperazine-1-carboxylate (155)**

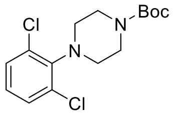

The title compound was synthesized using 2-bromo-1,3-dichlorobenzene (226 mg, 1.00 mmol, 1 eq.), *tert*-butyl piperazine-1-carboxylate (186 mg, 1.00 mmol, 1 eq.), sodium *tert*-butoxide (144 mg, 1.50 mmol, 1.5 eq.), rac-BINAP (37.4 mg, 0.06 mmol, 0.06 eq.) and palladium diacetate (8.96 mg, 0.04 mmol, 0.04 eq.) according to general procedure A in a yield of 35 mg (0.11 mmol, 11%). ^{1}H NMR (400 MHz, $CDCl_3$) δ 7.27 (d, *J* = 8.0 Hz, 2H), 6.99 (t, *J* = 8.3 Hz, 1H), 3.58 – 3.54 (m, 4H), 3.19 – 3.15 (m, 4H), 1.49 (s, 9H). ^{13}C NMR (101 MHz, $CDCl_3$) δ 155.08, 135.18, 129.25, 126.32, 79.76, 49.56, 28.59.

***tert*-Butyl (*S*)-4-(3-fluorophenyl)-3-methylpiperazine-1-carboxylate (156)**

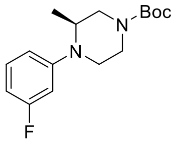

The title compound was synthesized using 1-bromo-3-fluorobenzene (175 mg, 1.00 mmol, 1 eq.), *tert*-butyl (*S*)-3-methylpiperazine-1-carboxylate (200 mg, 1.00 mmol, 1 eq.), sodium *tert*-butoxide (144 mg, 1.50 mmol, 1.5 eq.), rac-BINAP (37.4 mg, 0.06 mmol, 0.06 eq.) and palladium diacetate (8.96 mg, 0.04 mmol, 0.04 eq.) according to general procedure A in a yield of 177 mg (0.60 mmol, 60%). ^{1}H NMR (400 MHz, $CDCl_3$) δ 7.18 (td, *J* = 8.2, 7.0 Hz, 1H), 6.62 (dd, *J* = 8.3, 2.4 Hz, 1H), 6.58 – 6.44 (m, 2H), 4.36 – 3.70 (m, 3H), 3.40 – 2.96 (m, 4H), 1.49 (s, 9H), 1.02 (d, *J* = 6.5 Hz, 3H). ^{13}C NMR (101 MHz, $CDCl_3$) δ 164.00 (d, *J* = 243.2 Hz), 155.09, 151.72 (d, *J* = 9.8 Hz), 130.26 (d, *J* = 10.0 Hz), 111.50, 105.71 (d, *J* = 21.4 Hz), 103.05 (d, *J* = 25.3 Hz), 79.86, 51.12, 48.96, 47.69, 42.36, 28.43, 12.05.

***tert*-Butyl (*R*)-4-(3-chlorophenyl)-3-methylpiperazine-1-carboxylate ((*R*)-157)**

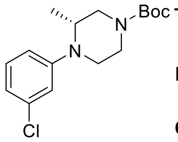

The title compound was synthesized using 1-bromo-3-chlorobenzene (191 mg, 1.00 mmol, 1 eq.), *tert*-butyl (*R*)-3-methylpiperazine-1-carboxylate (200 mg, 1.00 mmol, 1 eq.), sodium *tert*-butoxide (144 mg, 1.50 mmol, 1.5 eq.), rac-BINAP (37.4 mg, 0.06 mmol, 0.06 eq.) and palladium diacetate (8.96 mg, 0.04 mmol, 0.04 eq.) according to general procedure A in

a yield of 233 mg (0.75 mmol, 75%). ^{1}H NMR (400 MHz, $CDCl_3$) δ 7.15 (t, *J* = 8.1 Hz, 1H), 6.95 – 6.63 (m, 3H), 4.27 – 3.70 (m, 3H), 3.40 – 2.89 (m, 4H), 1.48 (s, 9H), 1.01 (d, *J* = 6.5 Hz, 3H). ^{13}C NMR (101 MHz, $CDCl_3$) δ 155.07, 151.17, 135.09, 130.19, 119.25, 116.31, 114.40, 79.87, 51.22, 49.11, 47.81, 42.84, 28.46, 12.16.

tert*-Butyl (*S*)-4-(3-chlorophenyl)-3-methylpiperazine-1-carboxylate** ((S***)-**157**)

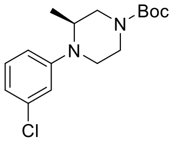

The title compound was synthesized using 1-bromo-3-chlorobenzene (191 mg, 1.00 mmol, 1 eq.), *tert*-butyl (*S*)-3-methylpiperazine-1-carboxylate (200 mg, 1.00 mmol, 1 eq.), sodium *tert*-butoxide (144 mg, 1.50 mmol, 1.5 eq.), rac-BINAP (37.4 mg, 0.06 mmol, 0.06 eq.) and palladium diacetate (8.96 mg, 0.04 mmol, 0.04 eq.) according to general procedure A in a yield of 117 mg (0.38 mmol, 38%). ^{1}H NMR (400 MHz, $CDCl_3$) δ 7.16 (t, *J* = 8.1 Hz, 1H), 6.93 – 6.44 (m, 3H), 4.23 – 3.71 (m, 3H), 3.41 – 2.92 (m, 4H), 1.48 (s, 9H), 1.02 (d, *J* = 6.5 Hz, 3H). ^{13}C NMR (101 MHz, $CDCl_3$) δ 155.12, 151.18, 135.12, 130.20, 119.31, 116.38, 114.51, 79.93, 51.25, 49.15, 42.92, 28.48, 12.19.

tert*-Butyl (*S*)-4-(3-bromophenyl)-3-methylpiperazine-1-carboxylate** ((S***)-**158**)

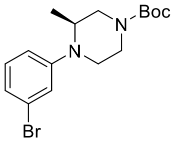

The title compound was synthesized using 1,3-dibromobenzene (236 mg, 1.00 mmol, 1 eq.), *tert*-butyl (*S*)-3-methylpiperazine-1-carboxylate (200 mg, 1.00 mmol, 1 eq.), sodium *tert*-butoxide (144 mg, 1.50 mmol, 1.5 eq.), rac-BINAP (37.4 mg, 0.06 mmol, 0.06 eq.) and palladium diacetate (8.96 mg, 0.04 mmol, 0.04 eq.) according to general procedure A in a yield of 154 mg (0.43 mmol, 43%). ^{1}H NMR (400 MHz, $CDCl_3$) δ 7.09 (t, *J* = 8.1 Hz, 1H), 7.02 – 6.89 (m, 2H), 6.78 (dd, *J* = 8.3, 2.4 Hz, 1H), 4.03 – 3.72 (m, 2H), 3.36 – 2.99 (m, 5H), 1.48 (s, 9H), 1.01 (d, *J* = 6.5 Hz, 3H). ^{13}C NMR (101 MHz, $CDCl_3$) δ 155.05, 151.31, 130.46, 123.37, 122.19, 119.26, 114.87, 79.87, 51.23, 49.12, 47.69, 42.78, 28.44, 12.18.

tert*-Butyl (*R*)-4-(3-bromophenyl)-3-methylpiperazine-1-carboxylate** ((R***)-**158**)

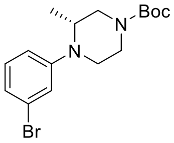

The title compound was synthesized using 1,3-dibromobenzene (236 mg, 1.00 mmol, 1 eq.), *tert*-butyl (*R*)-3-methylpiperazine-1-carboxylate (200 mg, 1.00 mmol, 1 eq.), sodium *tert*-butoxide (144 mg, 1.50 mmol, 1.5 eq.), rac-BINAP (37.4 mg, 0.06 mmol, 0.06 eq.) and palladium diacetate (8.96 mg, 0.04 mmol, 0.04 eq.) according to general procedure A in a yield of 257 mg (0.72 mmol, 72%). ^{1}H NMR (400 MHz, $CDCl_3$) δ 7.10 (t, *J* = 8.1 Hz, 1H), 7.03 – 6.90 (m, 2H), 6.78 (dd, *J* = 8.6, 2.4 Hz, 1H), 4.26 – 3.70 (m, 3H), 3.44 – 2.93 (m, 4H), 1.48 (s, 9H), 1.01 (d, *J* = 6.5 Hz, 3H). ^{13}C NMR (101 MHz, $CDCl_3$) δ 155.10, 151.35, 130.50, 123.41, 122.25, 119.30, 114.92, 79.93, 51.28, 49.20, 47.76, 42.95, 28.48, 12.22.

tert*-Butyl (*R*)-3-methyl-4-(3-(trifluoromethyl)phenyl)piperazine-1-carboxylate** ((R*)-159**)

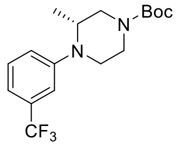

The title compound was synthesized using 1-bromo-3-(trifluoromethyl)benzene (225 mg, 1.00 mmol, 1 eq.), *tert*-butyl (*R*)-3-methylpiperazine-1-carboxylate (200 mg, 1.00 mmol, 1 eq.), sodium *tert*-butoxide (144 mg, 1.50 mmol, 1.5 eq.), rac-BINAP (37.4 mg, 0.06 mmol, 0.06 eq.) and palladium diacetate (8.96 mg, 0.04 mmol, 0.04 eq.) according to general procedure A in a yield of 176 mg (0.51 mmol, 51%). ^{1}H NMR (400 MHz, $CDCl_3$) δ 7.40 – 7.29 (m, 1H), 7.18 – 6.91 (m, 3H), 4.28 – 3.70 (m, 3H), 3.47 – 3.00 (m, 4H), 1.49 (s, 9H), 1.03 (d, *J* = 6.5 Hz, 3H). ^{13}C NMR (101 MHz, $CDCl_3$) δ 155.10, 150.24, 131.58 (q, *J* = 31.6 Hz), 129.73, 124.38 (q, *J* = 272.4 Hz), 119.26, 115.79, 112.70, 79.94, 51.23, 49.14, 47.83, 42.84, 28.42, 12.15.

tert*-Butyl (*S*)-3-methyl-4-(3-(trifluoromethyl)phenyl)piperazine-1-carboxylate** ((S*)-159**)

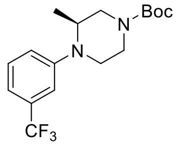

The title compound was synthesized using 1-bromo-3-(trifluoromethyl)benzene (225 mg, 1.00 mmol, 1 eq.), *tert*-butyl (*S*)-3-methylpiperazine-1-carboxylate (200 mg, 1.00 mmol, 1 eq.), sodium *tert*-butoxide (144 mg, 1.50 mmol, 1.5 eq.), rac-BINAP (37.4 mg, 0.06 mmol, 0.06 eq.) and palladium diacetate (8.96 mg, 0.04 mmol, 0.04 eq.) according to general procedure A in a yield of 176 mg (0.51 mmol, 51%). ^{1}H NMR (400 MHz, $CDCl_3$) δ 7.34 (t, *J* = 7.8 Hz, 1H), 7.15 – 6.90 (m, 3H), 4.03 – 3.77 (m, 3H), 3.41 – 2.98 (m, 4H), 1.49 (s, 9H), 1.03 (d, *J* = 6.5 Hz, 3H). ^{13}C NMR (101 MHz, $CDCl_3$) δ 155.06, 150.22, 131.55 (q, *J* = 31.7 Hz), 129.70, 124.36 (q, *J* = 272.4 Hz), 119.27, 115.75, 112.67, 79.90, 51.21, 49.09, 47.81, 42.81, 28.38, 12.09.

***tert*-Butyl 4-(3-chlorophenyl)-3,5-dimethylpiperazine-1-carboxylate** (**160**)

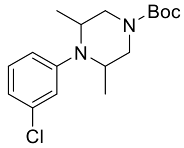

To a solution of 1-bromo-3-chlorobenzene (191 mg, 1 mmol, 1 eq.) and *tert*-butyl 3,5-dimethylpiperazine-1-carboxylate (257 mg, 1.2 mmol, 1.2 eq.) in dry 1,4-dioxane (2mL) was added KHMDS (239 mg, 1,2 mmol, 1.2 eq.) and the reaction mixture was heated to 100 °C and stirred overnight. The reaction progress was monitored by TLC analysis. Upon full conversion of the starting materials, the mixture was cool to room temperature, diluted with EtOAc, washed with water, dried ($MgSO_4$), filtered and concentrated under reduced pressure. The residue was purified by silica gel column chromatography (diethyl ether/pentane, 5%→20%) to afford the product (70 mg, 0.22 mmol, 22%).^{1}H NMR (400 MHz, $CDCl_3$) δ 7.22 (td, *J* = 8.2, 1.4 Hz, 1H), 7.06 (dt, *J* = 8.1, 1.3 Hz, 2H), 6.94 (dq, *J* = 8.0, 1.4 Hz, 1H), 3.80 (br, 2H), 3.38 – 2.81 (m, 4H), 1.50 (s, 9H), 0.84 (dd, *J* = 6.3, 1.3 Hz, 6H). ^{13}C NMR (101 MHz, $CDCl_3$) δ 154.75, 150.43, 134.70, 129.99, 124.18, 124.06, 122.50, 54.31, 28.54, 18.12.

***tert*-Butyl 4-(3-chlorophenyl)-2-methylpiperazine-1-carboxylate** (**161**)

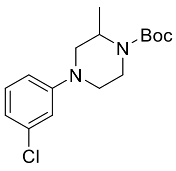

The title compound was synthesized using 1-bromo-3-chlorobenzene (96 mg, 0.50 mmol, 1 eq.), *tert*-butyl 2-methylpiperazine-1-carboxylate (100 mg, 0.50 mmol, 1 eq.), sodium *tert*-butoxide (72 mg, 0.75 mmol, 1.5 eq.), rac-BINAP (19 mg, 0.03 mmol, 0.06 eq.) and palladium diacetate (4.45 mg, 0.02 mmol, 0.04 eq.) according to general procedure A in a yield of 121 mg (0.39 mmol, 78%). The crude product was used in the next step without further purification.

***tert*-Butyl 4-(3-chlorophenyl)-3,3-dimethylpiperazine-1-carboxylate** (**162**)

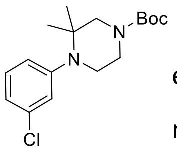

To a solution of *tert*-butyl 3,3-dimethylpiperazine-1-carboxylate (0.20 g, 0.93 mmol, 1 eq.) in anhydrous dioxane (3 mL) were added 1-bromo-2-chlorobenzene (179 mg, 0.93 mmol, 1 eq.) and KHMDS solution (1M in THF, 1.1 ml, 1.1 mmol, 1.2 eq.). The reaction mixture was stirred at RT for 2h. The reaction progress was monitored by TLC analysis. Upon full conversion of the starting materials, the mixture was diluted with DCM, washed with water, dried ($MgSO_4$), filtered and concentrated under reduced pressure. The crude product was purified using column chromatography (diethyl ether/Pentane, 5%-15%) to yield the product (86 mg, 0.27 mmol, 28%). ^{1}H NMR (400 MHz, $CDCl_3$) δ 7.18 (t, *J* = 8.1 Hz, 1H), 7.09 (d, *J* = 7.5 Hz, 2H), 6.98 (d, *J* = 8.0 Hz, 1H), 3.55 (t, *J* = 7.0 Hz, 2H), 3.32 (s, 2H), 3.05 (t, *J* = 5.2 Hz, 2H), 1.48 (s, 9H), 1.03 (s, 6H). ^{13}C NMR (101 MHz, $CDCl_3$) δ 154.88, 150.41, 133.71, 129.06, 127.58, 125.80, 124.78, 79.69, 56.45, 55.07, 46.91, 43.94, 28.49, 21.75.

(±) *tert*-Butyl 4-(4-chloropyridin-2-yl)-trans-2,3-dimethylpiperazine-1-carboxylate (**±163**)

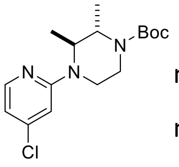

The title compound was synthesized using 2-bromo-4-chloropyridine (45 mg, 0.23 mmol, 1 eq.), (±) *tert*-butyl trans-2,3-dimethylpiperazine-1-carboxylate (50 mg, 0.23 mmol, 1 eq.) according to procedure A. This yielded the product (14.5 mg, 0.045 mmol, 19%). ^{1}H NMR (400 MHz, $CDCl_3$) δ 8.05 (d, *J* = 5.3 Hz, 1H), 6.59 (dd, *J* = 5.4, 1.6 Hz, 1H), 6.55 (s, 1H), 4.65 – 3.63 (m, 4H), 3.31 – 2.88 (m, 2H), 1.48 (s, 9H), 1.23 (d, *J* = 6.8 Hz, 3H), 1.17 (d, *J* = 6.7 Hz, 3H). ^{13}C NMR (101 MHz, $CDCl_3$) δ 160.09, 149.01, 145.17, 128.48, 113.23, 106.50, 80.00, 52.03, 50.61, 38.93, 37.43, 28.59, 17.27, 15.20.

***tert*-Butyl 3-chloro-4-(((3-methyl-1,2,4-oxadiazol-5-yl)methyl)sulfinyl)benzoate** (**164**)

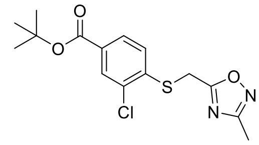

To a solution of *tert*-butyl 3-chloro-4-fluorobenzoate (200 mg, 0.867 mmol, 1 eq.), NaHS (97 mg, 1.73 mmol, 2 eq.) and 5-(chloromethyl)-3-methyl-1,2,4-oxadiazole (115 mg, 0.867 mmol, 1 eq.) were dissolved in DMF (2 ml). The mixture was

allowed to stir for 17 h. Then reaction was monitored by TLC analysis. Once complete, the mixture was diluted with DCM and washed with water and brine. The organic layer was dried over $MgSO_4$, filtered and concentrated. The residue was further purified by column chromatography (0-50% Et_2O in pentane). This yielded the product (65.5 mg, 0.192 mmol, 22%). ^{1}H NMR (400 MHz, $CDCl_3$) δ 7.98 (d, *J* = 1.8 Hz, 1H), 7.86 (dd, *J* = 8.3, 1.8 Hz, 1H), 7.45 (d, *J* = 8.3 Hz, 1H), 4.34 (s, 2H), 2.39 (s, 3H), 1.60 (s, 9H). ^{13}C NMR (101 MHz, $CDCl_3$) δ 175.18, 167.59, 164.03, 138.61, 133.12, 131.46, 130.56, 128.27 (d, *J* = 9.3 Hz), 127.88, 81.87, 28.13, 26.82, 11.59.

***tert*-Butyl 4-((2-ethoxyethyl)thio)-3-fluorobenzoate** (**165**)

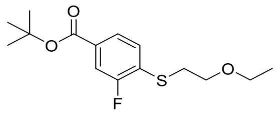

To a solution of *tert*-butyl 3,4-difluorobenzoate (191 mg, 0.89 mmol, 1eq.) in 5 ml DMF was added NaHS (50 mg, 0.89 mmol, 1 eq.), K_2CO_3 (370 mg, 0.89 mmol, 1 eq.) and 1-chloro-2-ethoxyethane (97 mg, 0.89 mmol, 1 eq.) and the mixture was stirred at 65 °C overnight under nitrogen. The reaction progress was monitored with TLC analysis. Once completed, the reaction mixture was diluted with diethyl ether, washed with water and dried over anhydrous $MgSO_4$. After filtration, the filtrate was concentrated under reduced pressure. The residue was purified by silica gel column chromatography (diethyl ether/pentane, 5-25%) to give the product (130 mg, 0.43 mmol, 49%). ^{1}H NMR (400 MHz, $CDCl_3$) δ 7.72 (dd, *J* = 8.1, 1.8 Hz, 1H), 7.61 (dd, *J* = 10.5, 1.7 Hz, 1H), 7.39 – 7.34 (m, 1H), 3.65 (t, *J* = 6.7 Hz, 2H), 3.51 (q, *J* = 7.0 Hz, 2H), 3.17 (t, *J* = 6.7 Hz, 2H), 1.59 (s, 9H), 1.19 (t, *J* = 7.0 Hz, 3H). ^{13}C NMR (101 MHz, $CDCl_3$) δ164.33, 159.91 (d, *J* = 245.1 Hz), 131.22 (d, *J* = 7.0 Hz), 129.76 (d, *J* = 17.4 Hz), 128.75, 125.37 (d, *J* = 3.3 Hz), 116.05 (d, *J* = 23.7 Hz), 81.52, 68.78, 66.55, 31.76, 28.13, 15.12.

***tert*-Butyl 3-chloro-4-(((3-methyl-1,2,4-oxadiazol-5-yl)methyl)sulfinyl)benzoate** (**166**)

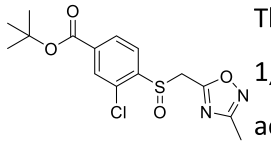

The title compound was synthesized using *tert*-butyl 3-chloro-4-(((3-methyl-1,2,4-oxadiazol-5-yl)methyl)sulfinyl) benzoate (65.0 mg, 0.191 mmol, 1eq.) according to procedure F. This yielded the product (71.1 mg, 0.199 mmol, quantitative). ^{1}H NMR (400 MHz, $CDCl_3$) δ 8.04 (dd, *J* = 8.1, 1.5 Hz, 1H), 8.01 (d, *J* = 1.3 Hz, 1H), 7.75 (d, *J* = 8.1 Hz, 1H), 4.47 (dd, *J* = 66.0, 13.8 Hz, 2H), 2.34 (s, 3H), 1.59 (s, 9H). ^{13}C NMR (101 MHz, $CDCl_3$) δ 169.65, 167.81, 163.34, 144.02, 136.82, 130.89, 130.19, 128.82, 126.40, 82.78, 49.80, 28.15, 11.61.

***tert*-Butyl 4-((2-ethoxyethyl)sulfinyl)-3-fluorobenzoate** (**167**)

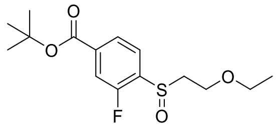

The title compound was synthesized using tert-butyl 4-((2-ethoxyethyl)thio)-3-fluorobenzoate (130 mg, 0.43 mmol, 1 eq.) according to procedure F. This yielded the product (97 mg, 0.31 mmol, 71%). ^{1}H NMR (400 MHz, $CDCl_3$) δ 7.99 (dd, *J* = 8.1, 1.5 Hz, 1H), 7.90 (dd, *J* = 8.1, 6.6 Hz, 1H), 7.71 (dd, *J* = 10.1, 1.4 Hz, 1H), 3.92 (dddd, *J* = 10.7, 8.6, 4.2, 0.8 Hz, 1H), 3.79 (dt, *J* = 10.4, 5.0 Hz, 1H), 3.50 (qq, *J* = 9.3, 7.0 Hz, 2H), 3.35 – 3.23 (m, 1H), 3.03 (dt, *J* = 13.5, 4.6 Hz, 1H), 1.61 (s, 9H), 1.15 (t, *J* = 7.0 Hz, 3H). ^{13}C NMR (101 MHz, $CDCl_3$) δ 163.61, 158.42, 155.96, 136.55, 135.96, 125.83, 116.57, 82.31, 66.66, 62.18, 55.10, 28.07, 14.93.

***tert*-Butyl 3-chloro-4-((2-ethoxy-2-oxoethyl)sulfonyl)benzoate** (**168**)

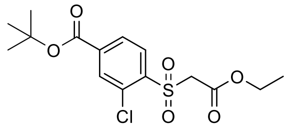

To a cooled (0 °C) solution of *tert*-butyl-3-chloro-4-((2-ethyoxy-2-oxoethyl)sulfinyl)benzoate (70 mg, 0.21 mmol, 1 eq.) in MeOH (7 mL) was added a solution of Oxone (390 mg, 0.64 mmol, 3 eq.) in H_2O (3.5 mL). The reaction mixture was stirred at RT overnight. The reaction progress was monitored by TLC analysis. Upon full conversion of the starting materials, the reaction mixture was extracted with DCM, dried ($MgSO_4$), filtered and concentrated under reduced pressure. The residue was purified by silica gel column chromatography (EtOAc / pentane, 0-15%) to yield the product (65 mg, 0.18 mmol, 85%). ^{1}H NMR (400 MHz, $CDCl_3$) δ 8.18 (d, *J* = 8.2 Hz, 1H), 8.14 (d, *J* = 1.5 Hz, 1H), 8.05 (dd, *J* = 8.3, 1.6 Hz, 1H), 4.47 (s, 2H), 4.12 (q, *J* = 7.1 Hz, 2H), 1.61 (s, 9H), 1.17 (t, *J* = 7.2 Hz, 3H). ^{13}C NMR (400 MHz, $CDCl_3$) δ 162.97, 162.11, 139.35, 138.42, 132.84, 132.72, 132.25, 128.11, 83.23, 62.70, 58.74, 28.17, 13.96.

3-Chloro-4-(((3-methyl-1,2,4-oxadiazol-5-yl)methyl)sulfinyl)benzoic acid (**169**)

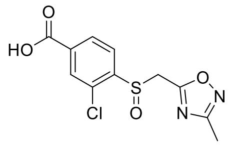

The title compound was synthesized using *tert*-butyl 3-chloro-4-(((3-methyl-1,2,4-oxadiazol-5-yl)methyl)sulfinyl) benzoate (68 mg, 0.191 mmol, 1eq.) according to procedure G. This yielded the product (47 mg, 0.156 mmol, 82%). ^{1}H NMR (400 MHz, Methanol-*d*4) δ 8.16 – 8.04 (dd, *J* = 10.2, 2.1 Hz, 2H), 7.69 (d, *J* = 8.1 Hz, 1H), 4.73 (dd, *J* = 69.2, 14.2 Hz, 2H), 2.28 (s, 3H). ^{13}C NMR (101 MHz, Methanol-*d*4) δ 171.62, 168.89, 167.08, 145.37, 137.13, 132.00, 131.67, 130.03, 127.52, 50.26, 11.19.

4-((2-Ethoxyethyl)sulfinyl)-3-fluorobenzoic acid (**170**)

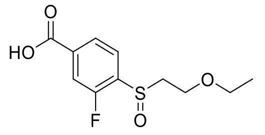

The title compound was synthesized using *tert*-butyl 4-((2-ethoxyethyl)sulfinyl)-3-fluorobenzoate (97 mg, 0.31 mmol, 1eq.) according to procedure G. This yielded the product (74 mg, 0.28 mmol, 93%). ^{1}H NMR (400

MHz, $CDCl_3$) δ 10.96 (br, 1H), 8.12 (dd, *J* = 8.1, 1.4 Hz, 1H), 7.99 (dd, *J* = 8.1, 6.6 Hz, 1H), 7.83 (dd, *J* = 9.8, 1.5 Hz, 1H), 4.15 – 3.74 (m, 2H), 3.66 – 3.33 (m, 3H), 3.19 (ddd, *J* = 13.5, 4.9, 3.8 Hz, 1H), 1.13 (t, *J* = 7.0 Hz, 3H). ^{13}C NMR (101 MHz, $CDCl_3$) δ 168.19, 157.39 (d, *J* = 248.4 Hz), 136.16, 134.75, 126.86 (d, *J* = 3.2 Hz), 126.40 (d, *J* = 2.2 Hz), 117.18 (d, *J* = 22.2 Hz), 66.83, 62.12, 54.80, 14.92.

(±) **2-Chloro-4-(4-(3-chlorophenyl)-*trans*-2,3-dimethylpiperazine-1-carbonyl)benzaldehyde (171)**

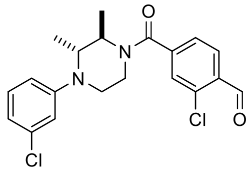

The title compound was synthesized using 3-chloro-4-formylbenzoic acid (65.0 mg, 35.0 mmol, 1 eq.), (±) 1-(3-chlorophenyl)-*trans*-2,3-dimethylpiperazine (79.0 mg, 0.35 mmol, 1 eq.), DiPEA (137 mg, 1.06 mmol, 3 eq.) and HATU (161 mg, 0.42 mmol, 1.2 eq.) according to general procedure H in a yield of 69.0 mg (0.18 mmol, 50%). NMR shows a mixture of rotamers. ^1H NMR (400 MHz, $CDCl_3$) δ 10.49 – 10.45 (m, 1H), 7.97 (d, *J* = 7.9 Hz, 1H), 7.49 (dd, *J* = 11.0, 1.5 Hz, 1H), 7.41 – 7.35 (m, 1H), 7.18 – 7.12 (m, 1H), 6.82 – 6.76 (m, 3H), 4.48 – 4.78 (m, 1H), 4.04 – 3.74 (m, 1H), 3.72 – 3.01 (m, 4H), 1.48 (d, *J* = 6.8 Hz, 1H), 1.45 (d, *J* = 6.8 Hz, 2H), 1.11 (d, *J* = 6.6 Hz, 1H), 1.00 (d, *J* = 6.6 Hz, 2H). ^{13}C NMR (101 MHz, $CDCl_3$) δ 188.9, 168.6, 168.2, 151.3, 142.7, 142.6, 138.4, 135.2, 132.9, 130.3, 129.9, 129.8, 129.1, 128.7, 125.6, 125.2, 119.4, 116.2, 114.2, 114.1, 60.4, 56.1, 55.4, 49.5, 42.2, 41.2, 40.4, 36.4, 17.7, 16.7, 14.2, 12.8, 12.5.

(±) 2-Chloro-4-(4-(3-chlorophenyl)-trans-2,3-dimethylpiperazine-1-carbonyl)benzoic acid (172)

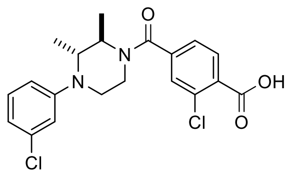

To a solution of (±) 2-chloro-4-(4-(3-chlorophenyl)-*trans*-2,3-dimethylpiperazine-1-carbonyl)benzaldehyde (69.0 mg, 176 µmol, 1 eq.) in DMF (1.0 ml) was added Oxone (108 mg, 176 µmol, 1 eq.) and the mixture was stirred at rt overnight. The reaction progress was monitored by TLC analysis. Upon full conversion of the starting materials, the reaction mixture was extracted with DCM, dried ($MgSO_4$), filtered and concentrated under reduced pressure. The residue was purified by silica gel column chromatography (MeOH/DCM, 2% - 5%) to yield the product (68.0 mg, 0.15 mmol, 50%). NMR shows a mixture of rotamers. ^1H NMR (500 MHz, $CDCl_3$) δ 8.03 (s, 1H), 7.74 (dd, *J* = 8.0, 3.4 Hz, 1H), 7.39 (dd, *J* = 10.4, 1.5 Hz, 1H), 7.28 – 7.22 (m, 1H), 7.17 – 7.10 (m, 1H), 6.80 – 6.74 (m, 2H), 6.71 – 6.65 (m, 1H), 4.75 (q, *J* = 6.9 Hz, 1H), 4.61 – 4.53 (m, 1H), 3.87 – 3.79 (m, 1H), 3.55 – 2.99 (m, 3H), 1.44 (d, *J* = 6.8 Hz, 1H), 1.44 (m, 2H), 1.08 (d, *J* = 6.7 Hz, 1H), 0.95 (d, *J* = 6.7 Hz, 2H). ^{13}C NMR (126 MHz, $CDCl_3$) δ 171.2, 169.6, 169.1, 151.4, 138.6, 135.7, 132.7, 130.9, 130.3, 128.8, 128.4, 125.2, 124.5, 119.3, 116.1, 114.2, 69.7, 56.1.

5,6-Dimethyl-2,3-dihydropyrazine (**173**)

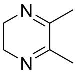
To a cooled (0 °C) solution of ethylenediamine (6.6 mL, 100 mmol) in Et_2O (250 mL) was dropwise added a solution of 2,3-butanedione (8.8 mL, 100 mmol) in Et_2O (250 mL) and the suspension was allowed to stir for 16 h. The resulting clear liquid was dried using potassium hydroxide for 30 min. After filtration, the mixture was concentrated and the residue was purified by short-neck distillation which yielded the product (9.4 g, 85 mmol, 85%). ^{1}H NMR (400 MHz, $CDCl_3$) δ 3.36 (s, 4H), 2.15 (s, 6H). ^{13}C NMR (101 MHz, $CDCl_3$) δ 159.5, 44.9, 23.4.

***cis*-2,3-Dimethylpiperazine** (**174**)

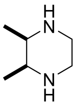
A solution of 5,6-dimethyl-2,3-dihydropyrazine (3.49 g, 31.7 mmol, 1 eq.) in EtOH (100mL) was degassed for 30 min by bubbling argon through the solution. Palladium loaded on carbon (10%, 3.2 g, 30.1 mmol) was added under constant bubbling. The solution was flushed three times with hydrogen after which the pressure was increased to 40 bar. The reaction mixture was stirred for 72 h on 40 bar. The suspension was filtered over celite and rinsed three times with EtOH (3x 50 mL). After evaporating the volatiles, the residue was purified by column chromatography (Et_2O:MeOH:NH_4OH, 10:4:1) to obtain the product (197 mg, 1.73 mmol, 5%). ^{1}H NMR (500 MHz, $CDCl_3$) δ 5.00 (s, 2H), 3.29-3.21 (m, 2H), 3.09-2.94 (m, 4H), 1.18 (d, J = 6.7 Hz, 6H). ^{13}C NMR (126 MHz, $CDCl_3$) δ 51.5, 40.9, 13.7.

(±) *trans*-2,3-Dimethylpiperazine (**±175**)

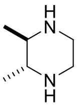
To a solution of 5,6-dimethyl-2,3-dihydropyrazine (9.36 g, 85 mmol, 1 eq.) in absolute ethanol (300 mL) was portion wise added sodium metal (23 g, 1 mol, 11.8 eq.) over six hours, after which the solution was refluxed for an additional 16 h. The slurry was neutralized by addition of acetic acid (50 mL) at 0 °C. The suspension was diluted with DCM, after stirring for 30 mins the precipitated sodium acetate was filtered off. The filtrate was concentrated under reduced pressure and the residue was purified by silica gel column chromatography (Et_2O : MeOH : NH_4OH, 10:4:1) to afford the product (3.71 g, 32.5 mmol, 38%). ^{1}H NMR (500 MHz, $CDCl_3$) δ 3.90 (s, 1H), 2.98 (m, 4H), 2.53 (m, 2H), 1.12-1.09 (m, 6H). ^{13}C NMR (126 MHz, $CDCl_3$) δ 57.2, 45.8, 18.5.

(±) *tert* Butyl trans-2,3-dimethylpiperazine-1-carboxylate (**±176**)

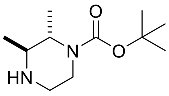
(±) *trans*-2,3-Dimethylpiperazine (262 mg, 2.29 mmol, 1 eq.) was dissolved in DCM (100 ml). Boc_2O (500 mg, 2.29 mmol, 1 eq.) dissolved in DCM (10 ml) was added slowly via a syringe pump over 45h. After adding, the reaction mixture was concentrated and the residue was purified by column chromatography (0-10% MeOH in DCM). This yielded the product as a yellow oil (282 mg, 1.31 mmol, 57%). ^{1}H NMR (400 MHz, $CDCl_3$) δ 5.19 (br, 1H), 3.92 (dd, J = 5.5, 1.6 Hz, 1H), 3.80

(d, J = 10.5 Hz, 1H), 3.12 – 2.71 (m, 4H), 1.38 (s, 9H), 1.24 (d, J = 5.2 Hz, 3H), 1.23 (d, J = 5.1 Hz, 3H). ^{13}C NMR (101 MHz, $CDCl_3$) δ 155.12, 79.87, 51.40, 50.70, 38.46, 37.41, 28.39, 17.21, 16.19.

2,2'-(1,2-Phenylenebis(azanediyl))bis(*N*-((*S*)-1-hydroxy-3-methylbutan-2-yl)benzamide) ((*S*)-177)

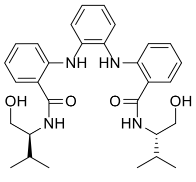

The title compound was synthesised from 2,2'-(1,2-phenylenebis(azanediyl))dibenzoic acid (530 mg, 1.52 mmol, 1 eq.) and (*S*)-2-amino-3-methylbutan-1-ol (345 mg, 3.35 mmol, 2.2 eq.) according to general procedure H. The crude product was purified by silica gel column chromatography (5% MeOH in DCM) to afford the title compound as pale yellow solid (470 mg, 0.906 mmol, 60%). ^{1}H NMR (500 MHz, $CDCl_3$) δ 8.47 (s, 2H), 7.37 (dd, J = 7.8, 1.6 Hz, 2H), 7.33 – 7.29 (m, 2H), 7.15 – 7.10 (m, 2H), 7.06 – 7.02 (m, 2H), 6.99 (d, J = 8.4 Hz, 2H), 6.66 (t, J = 7.5 Hz, 2H), 6.61 (d, J = 8.9 Hz, 2H), 4.06 (t, J = 5.8 Hz, 2H), 3.80 – 3.71 (m, 2H), 3.67 – 3.60 (m, 2H), 3.61 – 3.55 (m, 2H), 1.89 (hept, J = 6.6 Hz, 2H), 0.92 (t, J = 6.6 Hz, 12H).

2,2'-(1,2-Phenylenebis(azanediyl))bis(*N*-((*R*)-1-hydroxy-3-methylbutan-2-yl)benzamide) ((*R*)-177)

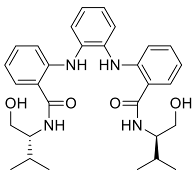

The title product was synthesised from 2,2'-(1,2-phenylenebis(azanediyl))dibenzoic acid (475 mg, 1.36 mmol, 1 eq.), and (*R*)-2-amino-3-methylbutan-1-ol (309 mg, 3.00 mmol, 2.2 eq.) according to general procedure H. The crude product was purified by silica gel column chromatography (5% MeOH in DCM) to afford the title compound as pale yellow solid (383 mg, 0.738 mmol, 54%). ^{1}H NMR (400 MHz, $CDCl_3$) δ 8.48 (s, 2H), 7.38 (dd, J = 7.8, 1.6 Hz, 2H), 7.31 (dd, J = 5.9, 3.6 Hz, 2H), 7.12 (td, J = 7.8, 7.2, 1.5 Hz, 2H), 7.07 – 6.97 (m, 4H), 6.73 (d, J = 8.9 Hz, 2H), 6.64 (t, J = 7.5 Hz, 2H), 4.19 (s, 2H), 3.80 – 3.69 (m, 2H), 3.66 – 3.51 (m, 4H), 1.86 (hept, J = 6.9 Hz, 2H), 0.90 (dd, J = 6.9, 4.6 Hz, 12H). ^{13}C NMR (101 MHz, $CDCl_3$) δ 170.15, 144.58, 134.50, 131.78, 127.75, 123.74, 122.53, 120.15, 118.22, 115.21, 62.84, 57.12, 29.05, 19.55, 18.96.

N^1,N^2-Bis(2-((*S*)-4-isopropyl-4,5-dihydrooxazol-2-yl)phenyl)benzene-1,2-diamine ((*S*)-178)

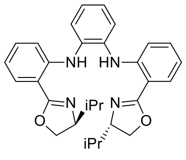

A solution of **(*S*)-178** (470 mg, 0.906 mmol, 1 eq.), triphenylphosphine (951 mg, 3.62 mmol, 4 eq.), Et_3N (0.50 mL, 3.6 mmol, 4 eq.), perchloromethane (0.35 mL, 3.6 mmol, 4 eq.) in ACN (9 mL) was stirred for 16 h. Thereafter, the mixture was concentrated and the residue was dissolved in DCM, washed with water, dried ($MgSO_4$), filtered and concentrated under reduced pressure. The crude product was purified by silica gel column chromatography (30% EtOAc in pentane) to afford the title product as an off-white solid (257 mg, 0.533 mmol, 59%). ^{1}H NMR (400 MHz, $CDCl_3$) δ 10.47 (s, 2H), 7.77 (d, J = 8.3 Hz, 2H), 7.57 – 7.49 (m, 2H), 7.29 – 7.18 (m, 4H), 7.09

(dd, J = 6.1, 3.5 Hz, 2H), 6.80 – 6.69 (m, 2H), 4.35 – 4.24 (m, 2H), 3.98 – 3.86 (m, 4H), 1.59 – 1.47 (m, 2H), 0.75 (t, J = 6.4 Hz, 12H). ^{13}C NMR (101 MHz, $CDCl_3$) δ 163.33, 146.04, 135.10, 131.75, 129.75, 123.48, 122.88, 116.72, 113.33, 110.85, 73.02, 69.08, 33.22, 18.70, 18.63.

N^1,N^2-Bis(2-((*R*)-4-isopropyl-4,5-dihydrooxazol-2-yl)phenyl)benzene-1,2-diamine ((*R*)-178)

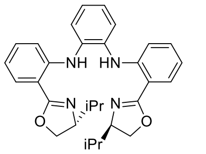

A solution of **(*R*)-178** (380 mg, 0.733 mmol, 1 eq.), triphenylphosphine (769 mg, 2.93 mmol, 4 eq.), Et_3N (0.40 mL, 2.9 mmol, 4 eq.), perchloromethane (0.28 mL, 2.9 mmol, 4 eq.) in ACN (7 mL) was stirred for 16 h. Thereafter, the mixture was concentrated and the residue was dissolved in DCM, washed with water, dried ($MgSO_4$), filtered and concentrated under reduced pressure. The crude product was purified by silica gel column chromatography (30% EtOAc in pentane) to afford the title product as an off-white solid (262 mg, 0.543 mmol, 74%). ^{1}H NMR (500 MHz, $CDCl_3$) δ 10.49 (s, 2H), 7.80 (dd, J = 7.7, 1.4 Hz, 2H), 7.55 (dd, J = 5.9, 3.6 Hz, 2H), 7.29 – 7.23 (m, 4H), 7.11 (dd, J = 6.0, 3.5 Hz, 2H), 6.76 (ddd, J = 8.1, 6.6, 1.7 Hz, 2H), 4.35 – 4.27 (m, 2H), 3.99 – 3.89 (m, 4H), 1.62 – 1.49 (m, 2H), 0.77 (dd, J = 6.8, 5.1 Hz, 12H). ^{13}C NMR (126 MHz, $CDCl_3$) δ 163.32, 146.03, 135.09, 131.74, 129.74, 123.47, 122.86, 116.71, 113.31, 110.84, 73.00, 69.06, 33.20, 18.69, 18.61.

Ethyl 2-((2-chloro-4-formylphenyl)thio)acetate (179)

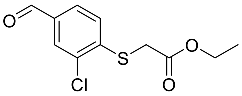

To a solution of 3-chloro-4-fluorobenzaldehyde (300 mg, 1.89 mmol, 1eq.) in DMF (5mL) were added K_2CO_3 (523 mg, 3.78 mmol, 2 eq.) and ethyl 2-mercaptoacetate (227 mg, 1.89 mmol, 2 eq.) and stirred at rt overnight. The reaction progress was monitored by TLC analysis. Upon full conversion of the starting materials, the reaction mixture was extracted with DCM, dried ($MgSO_4$), filtered and concentrated under reduced pressure. The residue was purified by silica gel column chromatography (Et_2O /pentane, 20-35%) to yield the product (446 mg, 1.72 mmol, 91%,). ^{1}H NMR (400 MHz, $CDCl_3$) δ 9.89 (s, 1H), 7.84 (d, J = 1.8 Hz, 1H), 7.72 (dd, J = 8.3, 1.8 Hz, 1H), 7.42 (d, J = 8.3 Hz, 1H), 4.22 (q, J = 7.1 Hz, 2H), 3.78 (s, 2H), 1.27 (t, J = 7.1 Hz, 3H). ^{13}C NMR (126 MHz, $CDCl_3$) δ 190.1, 168.5, 143.9, 134.6, 132.6, 130.1, 128.4, 126.3, 62.3, 34.2, 14.2.

(±) Ethyl 2-((2-chloro-4-(4-(3-chlorophenyl)-trans-2,3-dimethylpiperazin-1-yl)methyl)phenyl)thio)acetate (±180)

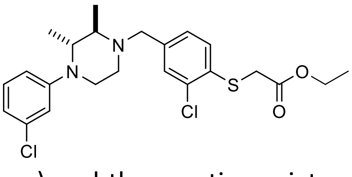

To a solution of ethyl-2-((2-chloro-4-formylphenyl)thio)acetate (31.8 mg, 0.12 mmol, 1.2 eq.) in 1,2-dichloroethane (1.0 mL) was added (±)1-(3-chlorophenyl)- trans-2,3-dimethylpiperazine (23.0 mg, 0.10 mmol, 1 eq.) and the reaction mixture was stirred at rt for 30min. Then sodium triacetoxyborohydride (65.1

mg, 0.31 mmol, 3 eq.) was added and the reaction mixture was stirred at rt overnight. The reaction progress was monitored by TLC analysis. Upon full conversion of the starting materials, the reaction mixture was extracted with DCM, dried ($MgSO_4$), filtered and concentrated under reduced pressure. The residue was purified by silica gel column chromatography (EtOAc/pentane, 20%) to yield the product (19 mg, 0.04 mmol, 40 %). ^{1}H NMR (400 MHz, $CDCl_3$) δ 7.46 (s, 1H), 7.39 (d, *J* = 8.0 Hz, 1H), 7.30 – 7.27 (m, 1H), 7.15 (t, *J* = 8.0 Hz, 1H), 6.84 – 6.79 (m, 1H), 6.73 (t, *J* = 8.0 Hz, 2H), 4.20 (q, *J* = 7.2 Hz, 2H), 3.75 – 3.67 (m, 3H), 3.57 (q, *J* = 13.9 Hz, 2H), 3.27 – 3.09 (m, 2H), 2.91 – 2.81 (m, 1H), 2.75 (td, *J* = 11.4 Hz, 1H), 2.52 (d, *J* = 11.5 Hz, 1H), 1.27 (d, *J* = 7.2 Hz, 3H), 1.24 – 1.19 (m, 3H), 1.15 (d, *J* = 6.3 Hz, 3H). ^{13}C NMR (101 MHz, $CDCl_3$) δ 169.4, 152.1, 140.2, 135.1, 134.6, 132.3, 130.5, 130.1, 129.9, 127.6, 118.0, 115.5, 113.6, 61.8, 58.0, 56.7, 56.6, 44.9, 42.0, 35.6, 14.2, 13.0, 9.5.

^{1}H NMR ±73 (LEI-515)

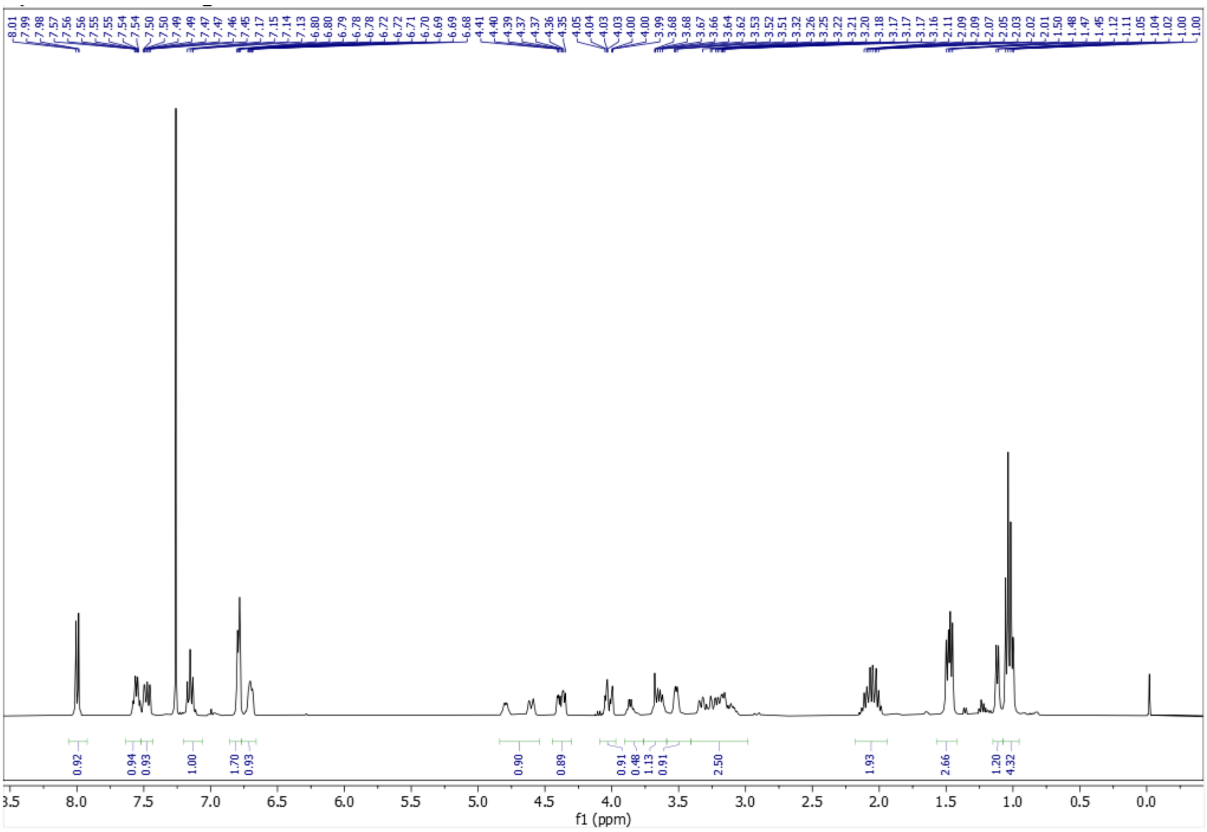

^{13}C NMR ±73 (LEI-515)

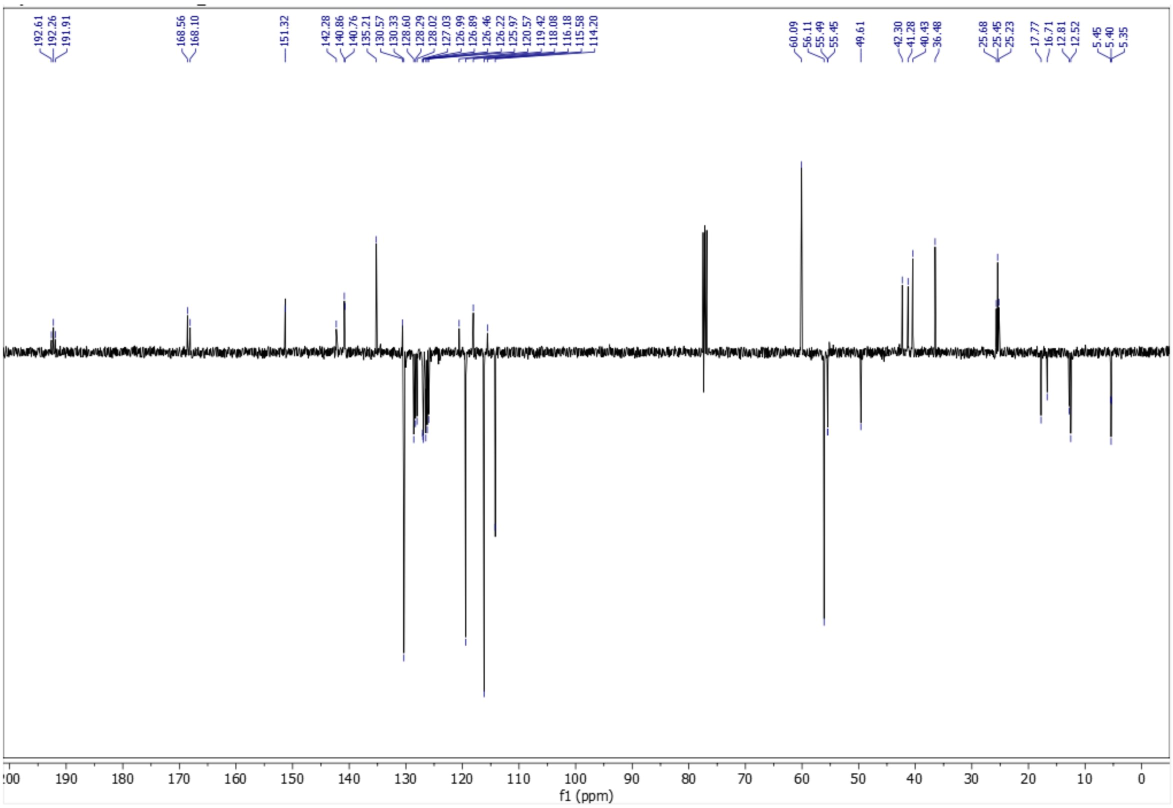

HPLC trace 10

10-90% (ACN in H_2O + 0.1% TFA), RT = 7.46 (m/z = 580.00), purity 99%.

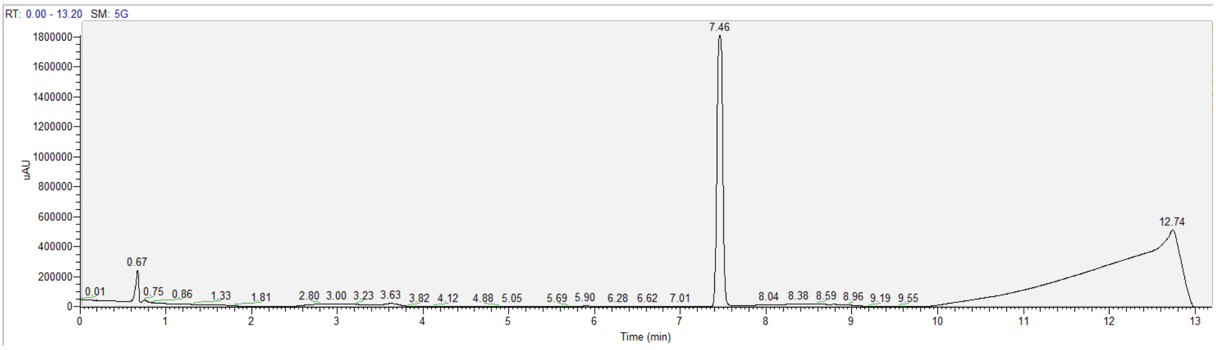

HPLC trace 14

10-90% (ACN in H_2O + 0.1% TFA), RT = 7.65 (m/z = 514.07), purity 98%.

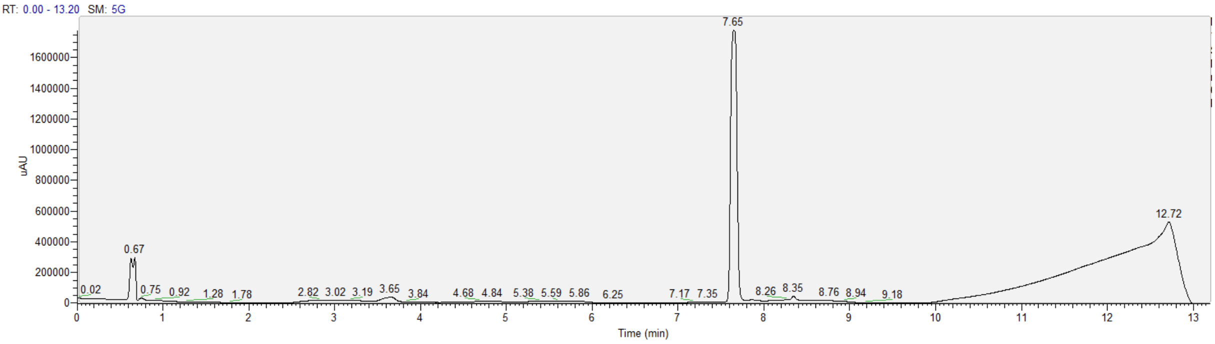

HPLC trace (R)-25

10-90% (ACN in H_2O + 0.1% TFA), RT = 7.47 (m/z = 538.07), purity 97%.

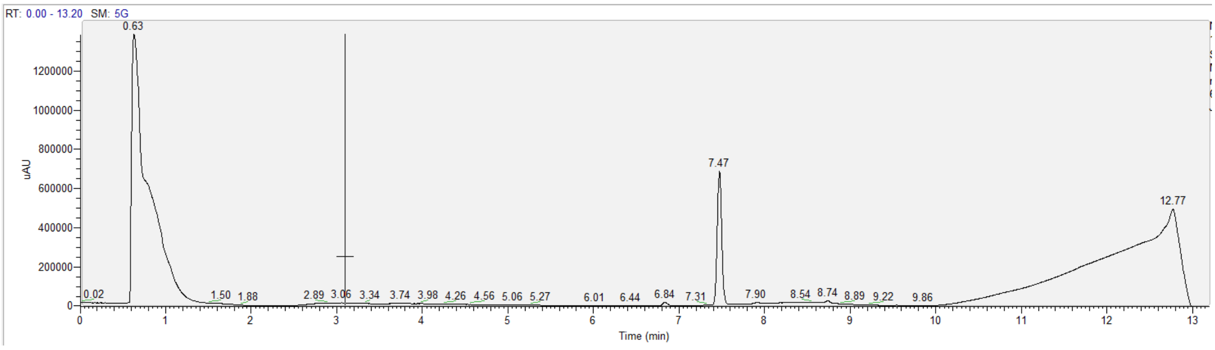

HPLC trace 27

10-90% (ACN in H_2O + 0.1% TFA), RT = 7.03 (m/z = 480.07), purity 97%.

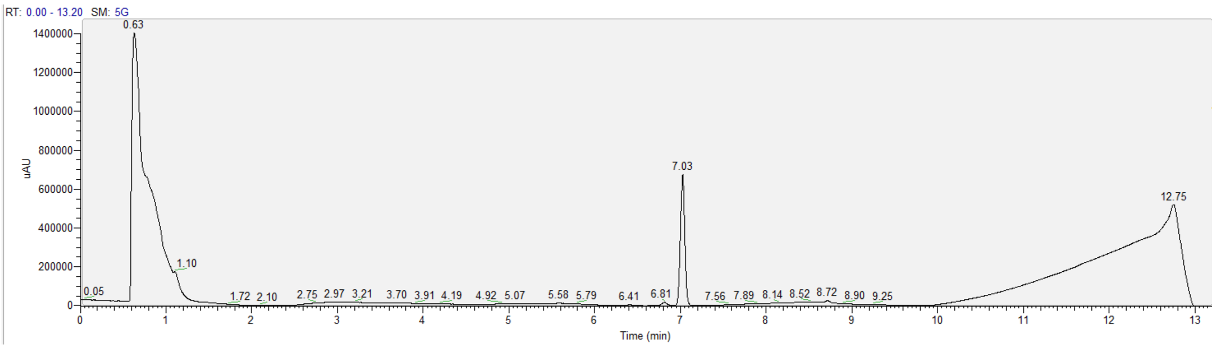

HPLC trace 34

10-90% (ACN in H_2O + 0.1% TFA), RT = 8.16 (m/z = 536.00), purity 95%.

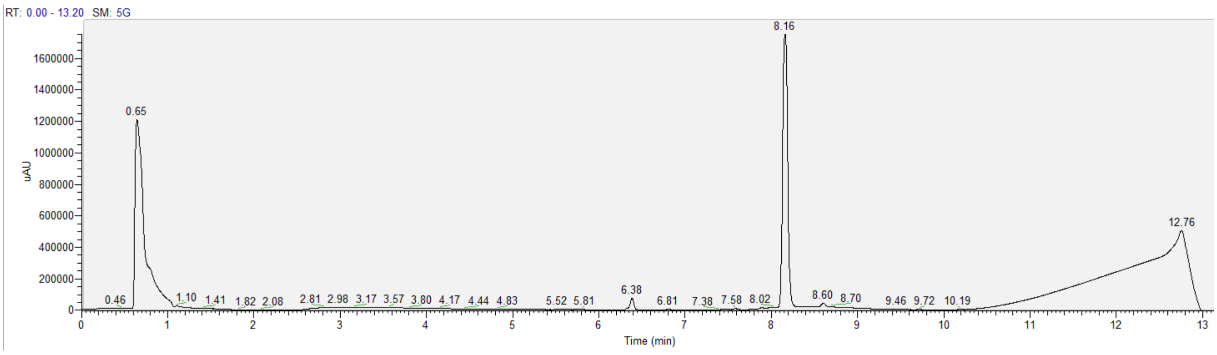

HPLC trace ±43

10-90% (ACN in H_2O + 0.1% TFA), RT = 8.21 (m/z = 497.07), purity 95%.

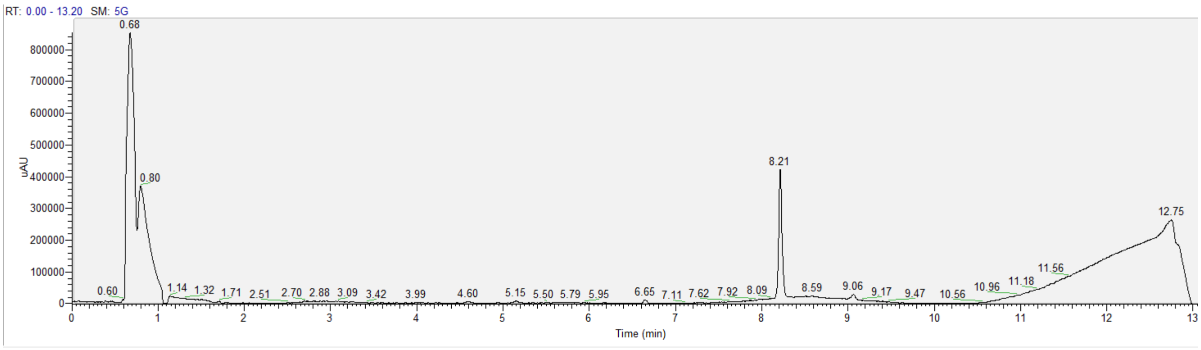

HPLC trace ±55

10-90% (ACN in H_2O + 0.1% TFA), RT = 6.87 (m/z = 543.07), purity 98%.

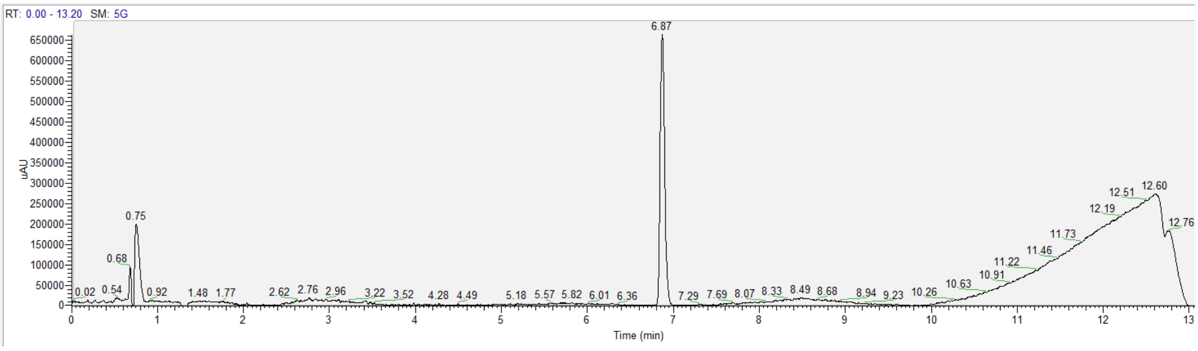

HPLC trace ±73 (LEI-515)

10-90% (ACN in H_2O + 0.1% TFA), RT = 7.40 hydrate (m/z = 549.33); 8.25 = ketone (m/z = 531.08), purity 99%.

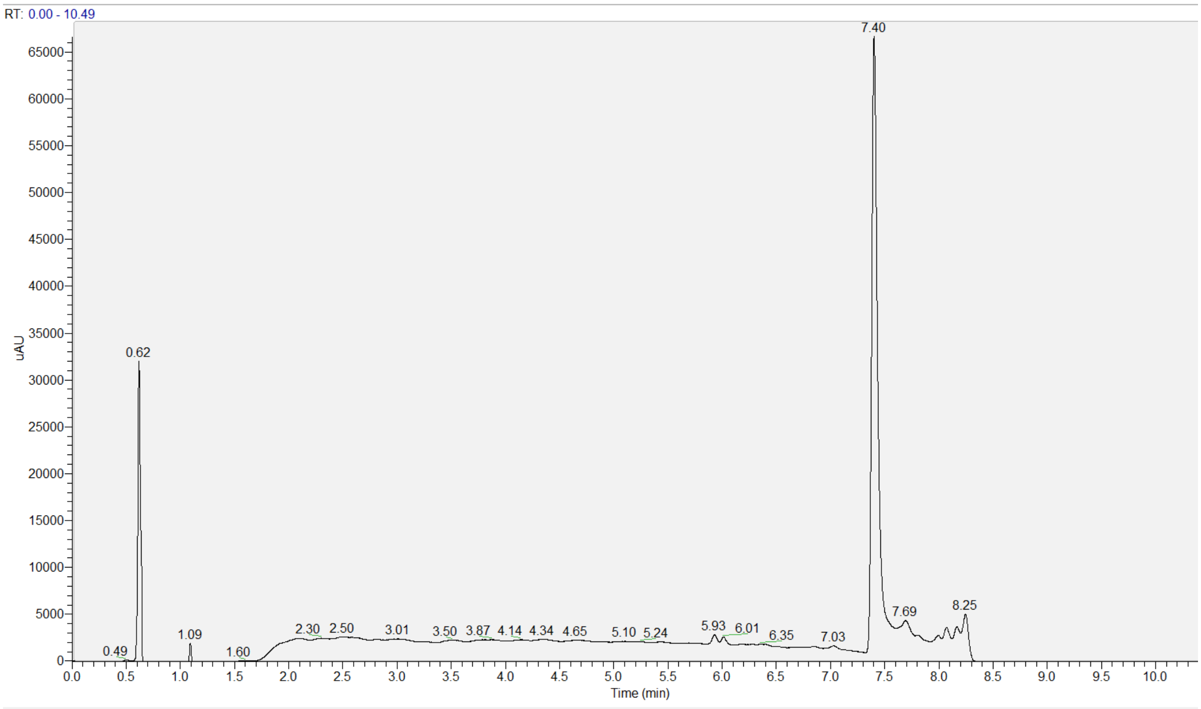

Chiral HPLC spectra of enantiomerically enriched sulfoxide intermediates ((+)-135 and (-)-135 respectively)

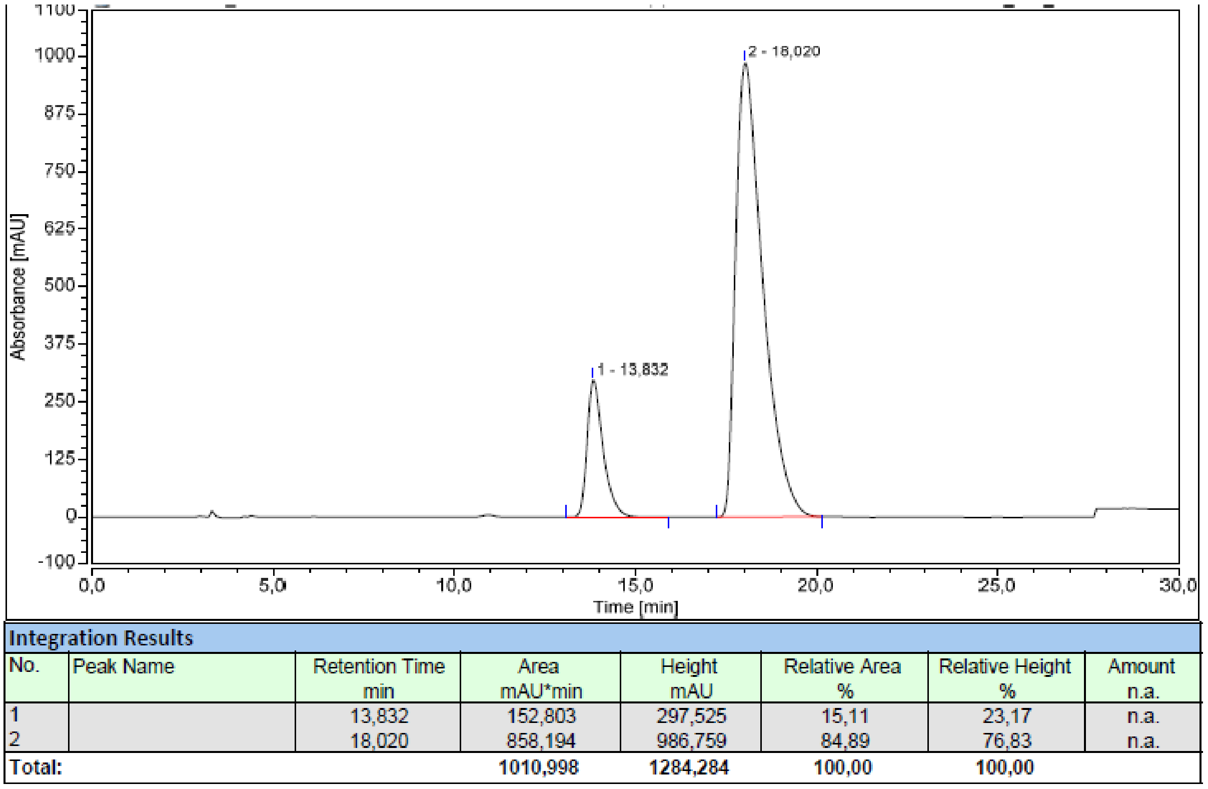

Integration Results							
No.	Peak Name	Retention Time min	Area mAU*min	Height mAU	Relative Area %	Relative Height %	Amount n.a.
1		13,832	152,803	297,525	15,11	23,17	n.a.
2		18,020	858,194	986,759	84,89	76,83	n.a.
**Total:**			**1010,998**	**1284,284**	**100,00**	**100,00**	

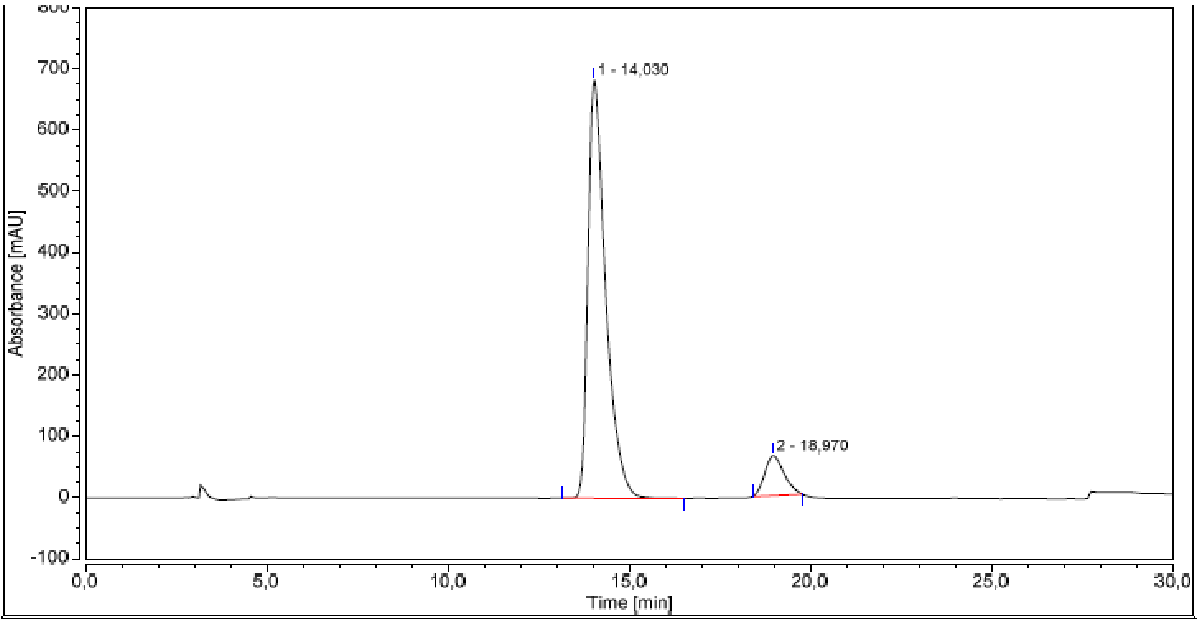

Integration Results							
No.	Peak Name	Retention Time min	Area mAU*min	Height mAU	Relative Area %	Relative Height %	Amount n.a.
1		14,030	385,379	682,527	90,56	91,35	n.a.
2		18,970	40,158	64,594	9,44	8,65	n.a.
**Total:**			**425,537**	**747,121**	**100,00**	**100,00**	
